# Determinants of synapse diversity revealed by super-resolution quantal transmission and active zone imaging

**DOI:** 10.1038/s41467-021-27815-2

**Published:** 2022-01-11

**Authors:** Zachary L. Newman, Dariya Bakshinskaya, Ryan Schultz, Samuel J. Kenny, Seonah Moon, Krisha Aghi, Cherise Stanley, Nadia Marnani, Rachel Li, Julia Bleier, Ke Xu, Ehud Y. Isacoff

**Affiliations:** 1 Department of Molecular and Cell Biology, University of California Berkeley, Berkeley, CA 94720 USA; 2 Helen Wills Neuroscience Institute, University of California Berkeley, Berkeley, CA 94720 USA; 3 Department of Chemistry, University of California, Berkeley, CA 94720 USA; 4 Molecular Biophysics and Integrated BioImaging Division, Lawrence Berkeley National Laboratory, Berkeley, CA 94720 USA; 5 Weill Neurohub, University of California Berkeley, Berkeley, CA 94720 USA

**Keywords:** Molecular neuroscience, Synaptic transmission, Super-resolution microscopy, Fluorescence imaging

## Abstract

Neural circuit function depends on the pattern of synaptic connections between neurons and the strength of those connections. Synaptic strength is determined by both postsynaptic sensitivity to neurotransmitter and the presynaptic probability of action potential evoked transmitter release (*P*_*r*_). Whereas morphology and neurotransmitter receptor number indicate postsynaptic sensitivity, presynaptic indicators and the mechanism that sets *P*_*r*_ remain to be defined. To address this, we developed QuaSOR, a super-resolution method for determining *P*_*r*_ from quantal synaptic transmission imaging at hundreds of glutamatergic synapses at a time. We mapped the *P*_*r*_ onto super-resolution 3D molecular reconstructions of the presynaptic active zones (AZs) of the same synapses at the *Drosophila* larval neuromuscular junction (NMJ). We find that *P*_*r*_ varies greatly between synapses made by a single axon, quantify the contribution of key AZ proteins to *P*_*r*_ diversity and find that one of these, Complexin, suppresses spontaneous and evoked transmission differentially, thereby generating a spatial and quantitative mismatch between release modes. Transmission is thus regulated by the balance and nanoscale distribution of release-enhancing and suppressing presynaptic proteins to generate high signal-to-noise evoked transmission.

## Abstract (web summary)

Super-resolution quantal imaging relates transmission at excitatory synapses to presynaptic molecular composition. The authors find that evoked transmission varies greatly between synapses and is uncorrelated and physically separate from spontaneous transmission, and identify responsible presynaptic proteins.

## Introduction

The operation of neural circuits depends on the synaptic connections between neurons. To understand how neural circuits process and store information, one needs to understand the molecular mechanisms that govern the synaptic transmission and distribute synaptic weights across large numbers of connections. While determinants of postsynaptic strength (e.g. dendritic spine size, postsynaptic scaffold size, number of postsynaptic receptors) are well characterized^1–4^, the presynaptic determinants are not as clear. The relationship between synapse morphology and presynaptic action potential (AP)-evoked neurotransmitter release probability (*P*_*r*_) is weak^5–10^ as is the dependence of *P*_*r*_ on specific elements of the transmitter release apparatus, the active zone (AZ)^11–18^.

To understand how presynaptic machinery governs quantal transmission, one needs to measure *P*_*r*_ at identified synapses whose molecular constituents and organization can be analyzed directly. Three approaches have been used to measure transmission at multiple identified synapses. Postsynaptic quantal (i.e. single synaptic vesicle resolution) imaging with Ca^2+^ indicators detects flux through ionotropic receptors as a proxy for the excitatory postsynaptic response^19–34^, biosensors detect released neurotransmitters^35^, and presynaptic synaptopHluorins detect vesicle fusion^36–38^. However, the diffraction-limited nature of these imaging paradigms makes it difficult to assign transmission events to particular synapses when AZs are densely arrayed.

To overcome these limitations, we developed a combination of super-resolution imaging modalities to precisely relate quantal transmission to synaptic architecture at the glutamatergic model synapse of the *Drosophila* NMJ. We used the logic of stochastic single-molecule super-resolution localization microscopy to develop Quantal Synaptic Optical Reconstruction (“QuaSOR”), analogous to recent super-resolution imaging of transmission in neuronal culture with synaptopHluorin and iGluSnFR^37,39,40^. QuaSOR resolved both action potential evoked and spontaneous quantal transmission events to individual synapses, even in regions where the synapses are crowded. QuaSOR allowed us to map locations of quantal transmission, quantify *P*_*r*_ using failure analysis and measure the frequency of spontaneous transmission (*F*_*s*_) at hundreds of synapses simultaneously throughout the NMJ, under physiological conditions. QuaSOR analysis was followed by super-resolution molecular imaging of presynaptic AZ proteins, enabling spatial averaging of protein and transmission localizations that revealed new aspects of synaptic release mechanisms.

We found that *P*_*r*_ has a high power dependence on the quantity of the presynaptic voltage-gated Ca^2+^ channel Cacophony (Cac)^41^, consistent with the power dependence of quantal content on Ca^2+^ ^42–45^. *P*_*r*_ also had a strong dependence on the scaffolding protein Bruchpilot (Brp), which organizes the AZ and anchors synaptic vesicles near the site of release^46–48^. However, Cac and Brp together accounted for only a minor fraction of the variance in *P*_*r*_, indicating that other important factors control and diversify AP-evoked release. A clue about one additional contributor came from an observation that evoked and spontaneous transmission modes are mismatched spatially and quantitatively. This led us to investigate Complexin (Cpx), whose *Drosophila* homolog is a powerful inhibitor of spontaneous transmitter release^49^ and which contains subdomains that both facilitate and inhibit evoked release^50^. As the Cpx/Brp ratio increased, *P*_*r*_ declined. When Cpx was knocked down, the mismatch between spontaneous and evoked transmission disappeared. Additionally, *P*_*r*_ was higher compared to control synapses with the same Brp content. We conclude that the interplay between release-promoting Cac and Brp and release-suppressing Cpx sets presynaptic transmission strength, generates synapse-to-synapse diversity, and enhances quantal signal-to-noise by suppressing spontaneous release at the site of maximal evoked release. The results demonstrate how super-resolution structure/function imaging can reveal the mechanisms of regulation of synaptic function.

## Results

### Super-resolution mapping of synaptic transmission sites and quantification of presynaptic strength

**Fig. 1:**
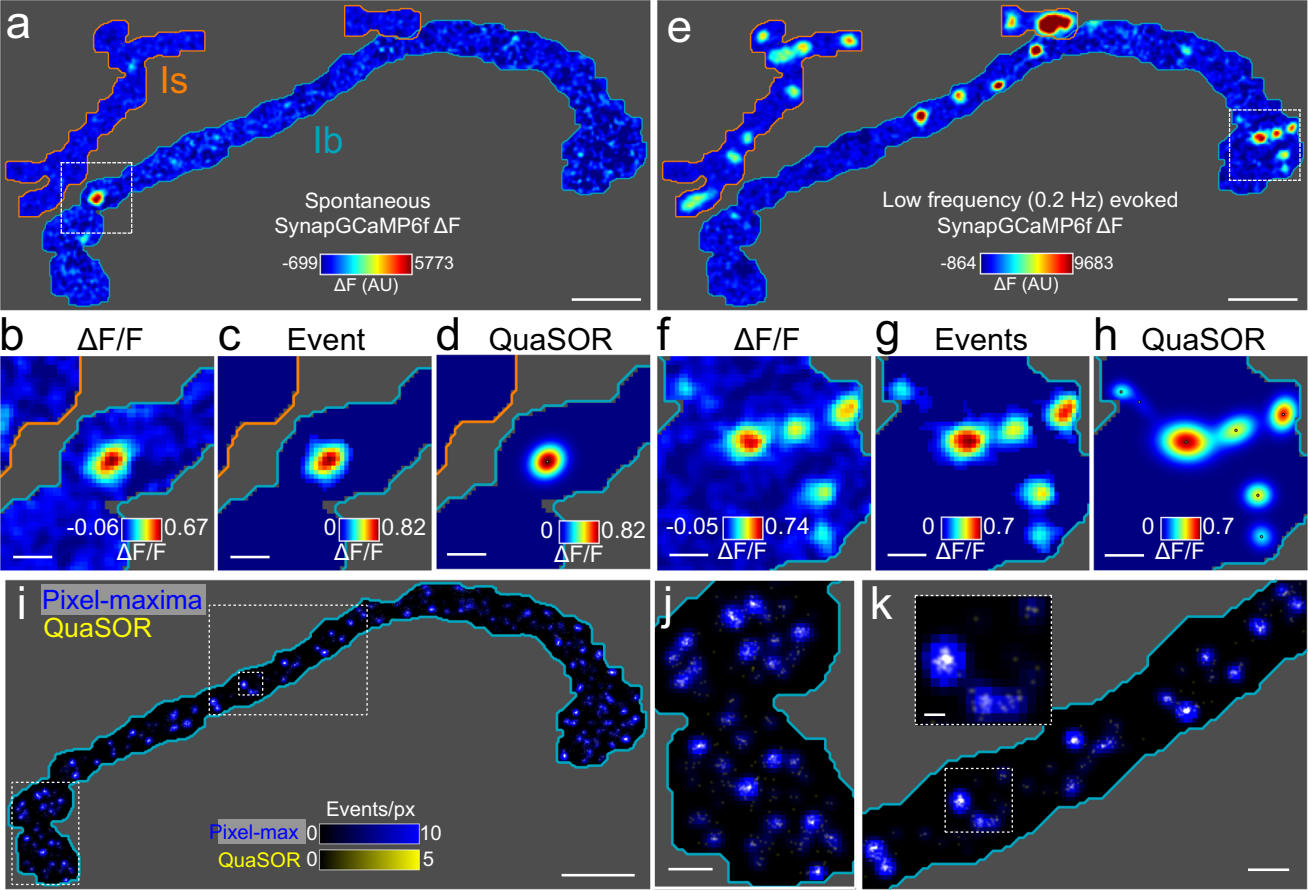
QuaSOR super-resolution mapping of spontaneous and evoked transmission. **a** Example spontaneous quantal event showing a full WT NMJ ∆F frame. Gray regions indicate areas of the muscle that were not associated with either Ib or Is NMJ and therefore not analyzed. **b** ∆F/F corresponding to boxed region in **a**. **c** Same event as in **a**, **b** showing isolated ∆F/F (only pixels associated with event). **d** QuaSOR-determined 2D Gaussian model for event in **a**–**c**. Black dot indicates maxima coordinate. **e** Single AP-evoked ∆F response frame. **f** ∆F/F frame corresponding to boxed region in **e**. **g** Same AP-evoked events as in **e**, **f** showing isolated ∆F/F. **h** QuaSOR-determined 2D Gaussian mixture model for 7 events in **g**. Black dots indicate maxima coordinates for each event component. **i**–**k** Comparison of AP-evoked pixel-maxima and QuaSOR mapping methods for the example Ib NMJ in **a**–**g** (pixel-maxima method blue, 211.6 nm/px, *σ* = 211.6 nm; QuaSOR yellow, 21.2 nm/px, *σ* = 42.3 nm; overlap white). Scale bars: 10 µm (**a**, **e**, **i**), 2 µm (**b**–**d**, **f**–**h**, **j**, **k**), and 500 nm (**k** inset).

Postsynaptic receptors at the *Drosophila* NMJ are Ca^2+^ permeable^51^, enabling detection of quantal, single synaptic vesicle transmission with the postsynaptically targeted genetically-encoded Ca^2+^ indicator SynapGCaMP6f^28^. However, diffusion of Ca^2+^ in the postsynaptic cytoplasm (Suppl. Fig. 1a–f) makes it challenging to separate quantal events arising at nearby synapses. Our earlier optical quantal analysis assigned transmission events to maximal fluorescence pixels and did not anchor these measurements to molecular maps of synapse location with sufficient resolution to resolve all synapses^28,33,34^. This resulted in events from neighboring synapses sometimes becoming conflated. We overcame this by developing QuaSOR, an analysis method that combines the fitting logic of single-molecule localization microscopy^52^, with the naturally low probability and stochastic nature of vesicle fusion at the NMJ to enhance spatial resolution. Similar strategies have been applied in neuronal culture for super-resolution synaptopHluorin and iGluSnFR imaging at single synapses^37,39,40^. We fitted two-dimensional (2D) asymmetric Gaussian functions to the Ca^2+^ signal for spontaneous and AP-evoked events (Fig. 1a–h and Suppl. Fig. 1g–j). With AP-evoked transmission, fitting was more challenging because events sometimes occurred synchronously at neighboring synapses (Fig. 1e, f). However, these responses were separated and resolved with 2D Gaussian mixture models (Fig. 1f–h and Suppl. Fig. 1i, j).

We measured synaptic release probability (*P*_*r*_) as the number of postsynaptic Ca^2+^ events evoked at each synapse by a train of motor nerve stimuli (evoked events/number of stimuli). This is the same as classical failure analysis but performed at individual synapses (rather than groups) and at physiological Ca^2+^ (i.e. at normal quantal content, rather than artificially lowered levels), where each stimulus evoked an event or failed to evoke an event. Comparing QuaSOR to our previous method of pixel-maxima mapping (Fig. 1i–k; Suppl. Fig. 1k–p), QuaSOR resolved evoked events with 3.8-fold higher spatial resolution (average half max cluster area for pixel-maxima = 0.171 ± 0.006 µm^2^ and for QuaSOR = 0.045 ± 0.002 µm^2^; *n* = 45 NMJs; see Methods section).

Most body wall muscles of *Drosophila* larvae are innervated by two glutamatergic motor axons, Type Ib and Is, which perform distinct functions during locomotion^28^. By overlaying QuaSOR maps of AP-evoked and spontaneous quantal transmission locations, we found a spatial mismatch between sites of spontaneous and evoked transmission (Suppl. Fig. 2a–c). QuaSOR analysis confirmed that Type Is synapses have an ~2.5-fold higher *P*_*r*_ than Ib synapses (Suppl. Fig. 2d, e)^28^.

### 3D-STORM of AZ molecular nanostructure

**Fig. 2:**
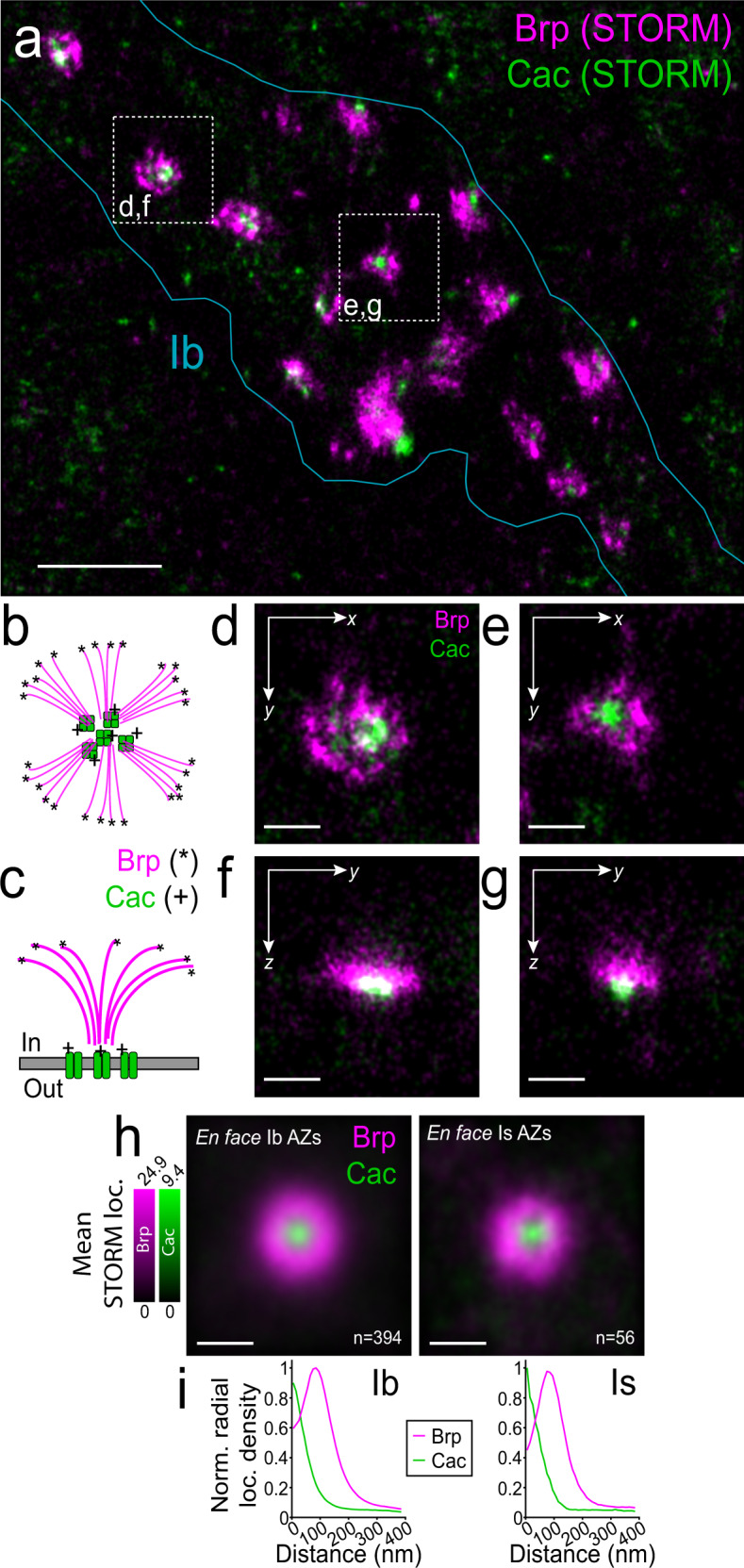
3D-STORM reconstructs Brp and Cac across synapses and orientations. **a** WT 3D-STORM *z*-projection of Brp (magenta) and Cac (green) localizations for a representative Ib bouton. **b**, **c** Schematics of the Brp and Cac molecule arrangements and their antibody epitopes (* Brp; +Cac) for *en face* AZs in **b** and side-on AZs in **c**. **d**–**g** 3D-STORM projection images of Brp (magenta) and Cac (green) for two* en face* AZs from the bouton in **a**. Shows *x-y* images with a *z*-projection in **c**, **d** and *y-z* images with an *x*-projection in **f**, **g**. **h** Pooled Ib and Is *en face* AZ alignments (all Ib AZs shown in Suppl. Video 2). Brp (magenta) and Cac (green) *z*-projection mean density images, aligned and averaged, for Ib AZs (left; *n* = 394 AZs from 9 NMJs) and Is AZs (right; *n* = 56 AZs from 9 NMJs). **i** STORM density radial profiles for Ib (left) and Is (right) *en face*-aligned AZ averages in **h**, normalized to the maximum radial density between the two axon types. Scale bars: 1 µm (**a**), 200 nm (**d**–**h**).

Next, in order to anchor sites of transmission to molecular maps of synapse locations, we followed QuaSOR with two-color 3D-STORM^53–56^. We initially focused on two key components of the presynaptic AZ: Brp, the CASK/ELKS-type scaffolding protein^46,47^, and Cac, the voltage-gated Ca^2+^ channel of the presynaptic release site^41,57^. To avoid potential mislocalization due to over-expression and/or fluorescent tags, we utilized antibodies to the native proteins (Suppl. Fig. 3). As shown previously with single-color 2D-STORM^58^, simultaneous two-color, 3D-STORM resolved AZs throughout the depth of the NMJ (Fig. 2a), providing a complete AZ map for multiple boutons, each containing many synapses (Suppl. Video 1 and Suppl. Fig. 6a–d). For a large number of AZs, Brp molecules were found to be arrayed in small clusters forming rings (Fig. 2a)^58^, whereas Cac in AZs was concentrated in small puncta^59^, often near Brp clusters, and embedded within Brp rings (Fig. 2a).

The relative positions of Brp and Cac within the AZ and 3D location of the AZ within the bouton, combined with the fact that the epitope for the Brp antibody lies at the Brp C-terminal, opposite from the plasma membrane end^48^, enabled us to identify *en face* oriented AZs (Fig. 2b–g), which aligns the maximal spatial *x*-*y* resolution of 3D-STORM imaging with the plane of the membrane. Following a strategy of STORM particle averaging^60–62^, we generated AZ-aligned localization density averages and radial density profiles for *en face* AZs (Fig. 2h and Suppl. Video 2). Consistent with earlier STED observations^59,63^, Brp was distributed as an annulus around Cac, with a maximum density ~100 nm from the Cac center (Fig. 2i). In order to validate the quantitative output of our STORM imaging, we first compared AZ architecture between Is and Ib inputs to the same muscle. Type Is AZs were slightly smaller than Ib AZs (Suppl. Fig. 4a, b), as observed previously^58^. Is AZs, had similar numbers of Cac localizations (Suppl. Fig. 4c, d) and very slightly fewer Brp localizations (Suppl. Fig. 4e, f). Because Is AZs were generally more compact, their Cac density was higher (Suppl. Fig. 4g, h), although their Brp density did not differ (Suppl. Fig. 4i, j). This suggests that the density of Ca^2+^ influx within the AZ may contribute to the higher *P*_*r*_ of Is synapses, as described in the calyx of Held^12^.

### Transmission diversity

**Fig. 3:**
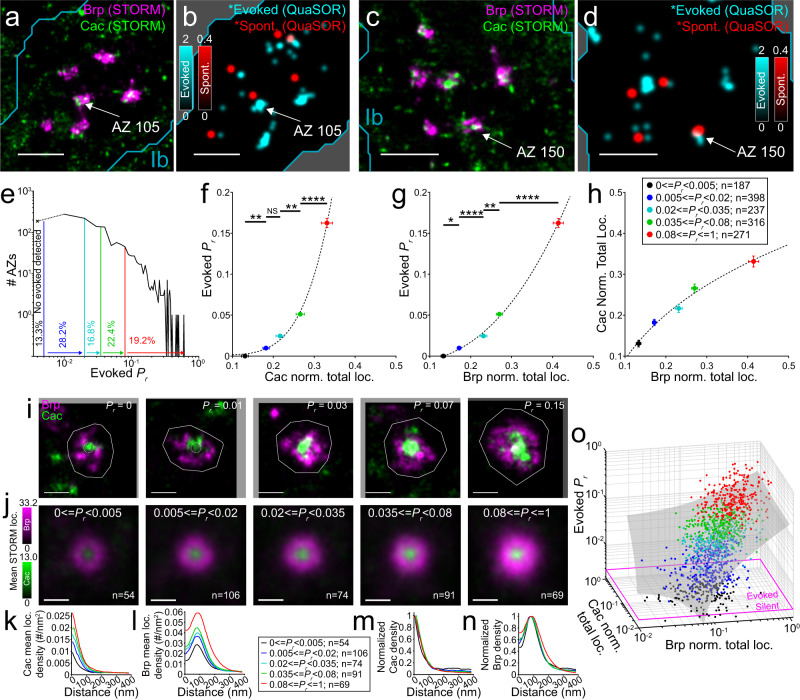
QuaSOR-STORM: Cac and Brp promote *P*_*r*_ but account for only a fraction of *P*_*r*_ variance. **a**–**d** QuaSOR-STORM alignment examples. WT Ib boutons showing Brp and Cac 3D-STORM *z*-projections (**a**, **c**) alongside STORM-aligned QuaSOR map (σ = 42.3 nm) for evoked (*; cyan) and spontaneous transmission (*; red) (**b**, **d**). **e** WT Ib AZ evoked *P*_*r*_ distribution with bin edges (see **f**–**h**) and percentages (*n* = 1409 QuaSOR-STORM matched Ib AZs, 9 NMJs; 200 stimuli at 0.2 Hz episodic protocol). (×) indicates fraction of sites with no detected evoked transmission. **f**, **g** *P*_*r*_ dependence on Cac (**f**) and Brp (**g**). *P*_*r*_-binned, mean NMJ-normalized total STORM localizations per AZ versus mean evoked *P*_*r*_ for all AZ orientations. (**f**, *R*^2^ = 0.998; *y* = 42*x*^**5.0**^ + 0.001; One-way ANOVA *p* = 5.8 × 10^−41^; Tukey-Kramer *post hoc* test; bin 1 vs bin 2 *p* = 0.0056; bin 2 vs bin 3 *p* = 0.097; bin 3 vs bin 4 *p* = 0.0063; bin 4 vs bin 5 *p* = 3.0 × 10^−5^; **g**, *R*^2^ = 0.998; *y* = 1.7*x*^**2.6**^ −0.01; One-way ANOVA *p* = 1.2 × 10^−119^; Tukey–Kramer post hoc test; bin 1 vs. bin 2 *p* = 0.012; bin 2 vs bin 3 *p* = 7.3 × 10^−9^; bin 3 vs bin 4 *p* = 0.0071; bin 4 vs bin 5 *p* = 9.9 × 10^−9^; mean ± SEM). **h** Non-linear Brp-Cac relationship. *P*_*r*_-binned, mean NMJ-normalized total STORM localizations per AZ for Brp versus Cac (*R*^2^ = 0.987; *y* = 1.2*x*^**0.2**^ − 0.65). **i** Representative *en face* AZs (from same NMJ) showing 3D-STORM overlay *z*-projections for Brp (magenta) and Cac (green) for each *P*_*r*_-binned group in **e**. AZ borders (solid white lines). Core 40 nm radial region (white dotted circles). **j** Average Brp and Cac densities across *P*_*r*_. Aligned, spatially averaged *en face* projections for Brp (magenta) and Cac (green) from bins in **e** (9 NMJs). **k**, **l** Radial mean STORM localization density profiles for *en face* AZs showing Cac (**k**) and Brp (**l**). **m**, **n** Relative Cac (**m**) and Brp (**n**) radial profile dimensions across *P*_*r*_ showing bin-normalized mean radial density profiles for corresponding *en face* AZ profiles (**k**, **l**). **o** Pooled NMJ-normalized total Brp versus Cac total STORM localizations *P*_*r*_ for all AZ orientations. *P*_*r*_ detection threshold (magenta line). 3D fit (gray plane) is linear polynomial surface (*R*^2^ = 0.312 *P*_*r*_ = −0.014 + 0.21*Brp* + 0.053*Cac*; *n* = 1409 AZs, 9 NMJs). Scale bars: 1 µm (**a**–**d**); 200 nm (**i**, **j**).

Having established QuaSOR for mapping transmission site localizations and measuring *P*_*r*_ and *F*_*s*_, along with two-color 3D-STORM reconstruction of AZs, we could now relate transmission at identified synapses to the molecules of their release apparatus (Fig. 3a–d and Suppl. Fig. 6). Low frequency (0.2 Hz) motor nerve stimulation evoked transmission at only ~5% of synapses per stimulus (Suppl. Fig. 7a, b). *P*_*r*_ varied between synapses over a remarkable range of ~100-fold (*P*_*r*_ = 0.005–0.610) and half of the synapses (49.8 ± 1.8%; 2233 AZs from 16 NMJs) had a very low *P*_*r*_ (≤0.02) (Fig. 3e). Spontaneous transmission occurred at low rates (Suppl. Fig. 7c) with a maximal local spontaneous release frequency (*F*_*s*_) of < 0.07 Hz, so that only ~0.5% of Ib synapses had a spontaneous event each second (Suppl. Fig. 7d). This extremely low spontaneous transmission frequency is consistent with findings in the intact, restrained animal^28^. We confirmed the large representation of low *F*_*s*_ and low *P*_*r*_ synapses in the second set of experiments, where we employed an alternative live imaging protocol (intermingling 100 stimuli to assess *P*_*r*_ with a total of 10 min of continuous imaging of spontaneous transmission) and replaced STORM with 3D Airyscan imaging^64^ to also exceed diffraction-limited resolution and also image larger fields, thereby capturing larger numbers of synapses (Suppl. Fig. 8). Again, approximately half of the synapses had a *P*_*r*_ < 0.02 (56.4 ± 5.0%). The longer period of continuous imaging provided a better measure of spontaneous transmission, revealing a maximal *F*_*s*_ of 0.063 Hz at WT Ib synapses with an average *F*_*s*_ of 0.0042 ± 0.00011 Hz (2209 synapses from 7 NMJs).

In a previous study^33^, we observed areas of the NMJ that lacked evoked transmission but showed spontaneous transmission, but, without a corresponding high-resolution view of synapse organization, we could not distinguish if these were individual AZs, clusters of AZs or non-synaptic sites where passing synaptic vesicle fuse spontaneously. Our current matching of AZ locations to QuaSOR transmission sites solved this problem and showed that 15.2 ± 1.5% of AZs had no evoked transmission (during 200 APs; *n* = 2233 AZs from 16 Ib NMJs; QuaSOR-STORM matched; mean ± SEM). Our second experimental paradigm, which provided a much longer observation time for spontaneous transmission (100 stimuli and 10 min spontaneous; *n* = 2209 AZs from 7 Ib NMJs; QuaSOR-Airyscan matched), showed that 24.8 ± 4.2% of AZs had no spontaneous activity. The majority of synapses with no evoked activity (over 100 stimuli) had spontaneous activity (67.4 ± 4.6%), indicating the presence of functional postsynaptic glutamate receptors, and suggesting that these synapses have an extremely low *P*_*r*_.

### Dependence of AP-evoked transmission on Brp and Cac

To understand the presynaptic mechanisms that set transmission strength, we examined the relationship between AP-evoked transmission and the Brp/Cac content of the presynaptic AZ. We grouped synapses into five bins: evoked-undetected (*P*_*r*_ < 0.005) and four sets of evoked-active sites spanning the range of *P*_*r*_ (*P*_*r*_ = 0.005–0.02, *P*_*r*_ = 0.02–0.035, *P*_*r*_ = 0.035–0.08, and *P*_*r*_ = 0.08–0.61) (Fig. 3e). We found that *P*_*r*_ had a 5^th^ power dependence on the normalized number of Cac localizations per AZ (Fig. 3f), consistent with the power dependence of quantal content on Ca^2+^ concentration^42,43^. *P*_*r*_ also had a super-linear dependence on Brp content (Fig. 3g) and Cac was sub-linearly dependent on Brp (Fig. 3h).

Beyond differences in the quantity of proteins at the AZ, differences in their nano-distribution may be an important diversifier of synaptic strength. *En face* synapses, binned according to *P*_*r*_, were aligned to generate function-grouped spatial density averages (Fig. 3i, j). The densities of Cac and Brp increased with *P*_*r*_ (Fig. 3k, l), but the relative shape of their radial profiles remained remarkably constant (Fig. 3m, n). *P*_*r*_ had the same 5^th^ power dependence on Cac within the 40 nm radius of the AZ core (Suppl. Fig. 9a) as seen for total Cac content across all AZ orientations (Fig. 3f).

We observed a strikingly large scatter in the three-way relationship between Cac and Brp and *P*_*r*_ (Fig. 3o). Active synapses with very different *P*_*r*_ sometimes had similar levels of Cac and/or Brp, and synapses with no detected evoked transmission showed a wide range of Cac and/or Brp. Together Cac and Brp only explained ~31% of the variance in *P*_*r*_ (Fig. 3o). Thus, *P*_*r*_ depends on the quantities of Cac and Brp, but additional factors must also contribute as there remains a large scatter that is not accounted for by these two molecules.

In earlier work^28^, before we increased the resolution of quantal transmission imaging with QuaSOR, we reduced the difficulty of resolving transmission at individual synapses by using a mutant of *rab3* (*rab3*^*rup*^) that redistributes Brp into a smaller number of enlarged AZs, separated by greater distance. We now asked whether the altered Brp architecture in *rab3*^*rup*^ would influence the dependence of synaptic transmission on Brp and Cac. STORM imaging confirmed that the *rab3*^*rup*^ mutant has enlarged AZs (Suppl. Fig. 5a, b), with more Brp and more Cac at both Ib and Is AZs (Suppl. Fig. 5c, d) and a greater number of Cac clusters (Suppl. Fig. 5a, b). QuaSOR/STORM alignment between transmission sites and AZs was readily done in *rab3*^*rup*^ (Suppl. Fig. 10a, b). We found that *rab3*^*rup*^ synapses have a higher average *P*_*r*_ and a broader *P*_*r*_ range than wildtype (Suppl. Fig. 10c). As seen in wildtype, the *F*_*s*_-*P*_*r*_ relation of *rab3*^*rup*^ was shallow, with little difference in spontaneous transmission frequency over a very wide range of *P*_*r*_ (Suppl. Fig. 10e). Also as in wildtype, in *rab3*^*rup*^, spontaneous transmission sites often lay between evoked sites (Suppl. Fig. 10b). We found that, although it was spread over the wider *P*_*r*_ range, the relationship between *P*_*r*_ and Cac in *rab3*^*rup*^ had a similar power dependence to that seen in WT (Suppl. Fig. 10d). These observations are consistent with a mechanism that tunes *P*_*r*_ by regulating the size of the Brp scaffold and the number of Cac channels and clusters.

### Spontaneous and evoked quantal transmission is decoupled

**Fig. 4:**
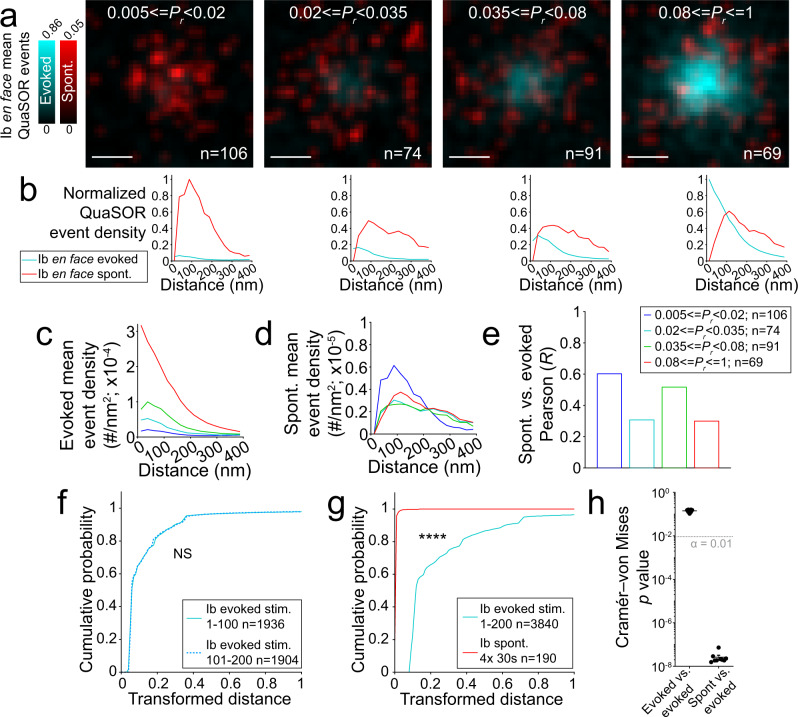
Evoked and spontaneous transmission are spatially mismatched. **a** Evoked and spontaneous average event densities across *P*_*r*_. *P*_*r*_-binned, aligned and averaged *en face* classified QuaSOR spatial patterns for evoked-active Ib AZs showing both evoked (cyan) and spontaneous (red) events (*σ* = 21.2 nm). **b** QuaSOR radial event density profiles across *P*_*r*_. *P*_*r*_-binned, normalized *en face* radial mean evoked and spontaneous density profiles for event distributions in **a**. Normalization is calculated from the maximum profile density for each event type. **c**, **d** Aligned *P*_*r*_-binned *en face*-defined radial mean event density profiles for evoked (**c**) and spontaneous (**d**) events. **e** Spatial decoupling of evoked and spontaneous radial profiles. Pearson correlation coefficients (*R*) for evoked versus spontaneous *P*_*r*_-binned *en face*-defined radial mean density profiles for each evoked-active *P*_*r*_ bin in **a**–**d**. **f** Global evoked pattern stability across stimulus trials. Representative transformed distance distributions for a single NMJ (example in Suppl. Fig. 6) comparing evoked event coordinates from stimuli 1–100 (solid; *n* = 1936 events) to evoked coordinates from stimuli 101–200 (dashed; *n* = 1904 events; Cramér–von Mises *p* = 0.14). **g** Global spontaneous and evoked pattern decoupling. Representative transformed distance distributions for the same NMJ in **f** comparing evoked event coordinates from all stimuli 1–200 (cyan; *n* = 3840 events) versus all spontaneous event coordinates (red; *n* = 190 events; two-sample two-sided Cramér–von Mises test *p* = 1.56 × 10^−7^). **h** Global decoupling of evoked and spontaneous release. Pooled two-sample two-sided Cramér–von Mises test *p* values for evoked first half versus evoked second half (**f**) and all evoked stimuli versus spontaneous (**g**) cumulative distributions for multiple WT NMJs (*n* = 9 NMJs). The dotted line indicates an *α* = 0.01. Scale bars: 200 nm (**a**).

Spontaneous and evoked transmission at individual synapses were positively related, but the relationship was extremely weak, with small changes in *F*_*s*_ over a wide range of *P*_*r*_ (Suppl. Fig. 9b). *F*_*s*_ depended on the quantity of both Cac (Suppl. Fig. 9c) and Brp (Suppl. Fig. 9d), but these relations were also shallow, in contrast to the steep power dependence of *P*_*r*_ on these proteins (Fig. 3f, g). These observations suggested differential molecular regulation of these two modes of release. To analyze this further, we compared the relative locations of spontaneous and evoked transmission events at *en face* synapses, across the *P*_*r*_ range (Fig. 4a). Radial profile analysis for evoked and spontaneous event densities showed that over an ~20-fold range in *P*_*r*_, there were modest differences in spontaneous transmission density (Fig. 4b–d). In fact, the highest density of spontaneous activity was observed at very low *P*_*r*_ synapses (Fig. 4d). Beyond this quantitative mismatch, we also observed a striking spatial mismatch between sites of evoked and spontaneous transmission. Sites of spontaneous transmission were displaced to the periphery of sites of evoked transmission, with complete suppression of spontaneous transmission at evoked transmission maxima (Fig. 4a–d) and, consequently, a weak cross-correlation between radial profiles of spontaneous and evoked transmission (Fig. 4e).

To obtain an unbiased estimate of the degree of overlap between the locations of spontaneous and evoked transmission events throughout the NMJ, across synapse orientations, we performed a global QuaSOR event location pattern analysis. Based on previous analyses of spatial point processes^65^, this provides a test for whether two sets of coordinates are statistically distinct spatially (see Methods section). It is not an analysis of individual synapses, which would require longer stimulus trains, but a gestalt analysis of regions of the NMJ, which takes an event and computes a cumulative probability of another event within multiple radii. We found that coordinates of AP-evoked transmission events differed greatly from coordinates of spontaneous transmission events (Fig. 4g, h), confirming the spatial mismatch. In contrast, release patterns did not differ significantly between the first and second halves of the evoked stimulus train (Fig. 4f, h).

Thus, so far, we were able to align super-resolution QuaSOR maps of spontaneous and evoked transmission dynamics onto super-resolution STORM maps of AZ location, nanoarchitecture, and protein content and thereby assess the possible contribution of AZ proteins to transmitter release. We observe a striking mismatch between sites of evoked and spontaneous transmission. We next sought to identify additional proteins that contribute to *P*_*r*_ diversity and spatial decoupling of transmission modes.

### Complexin nano-distribution and transmission regulation

**Fig. 5:**
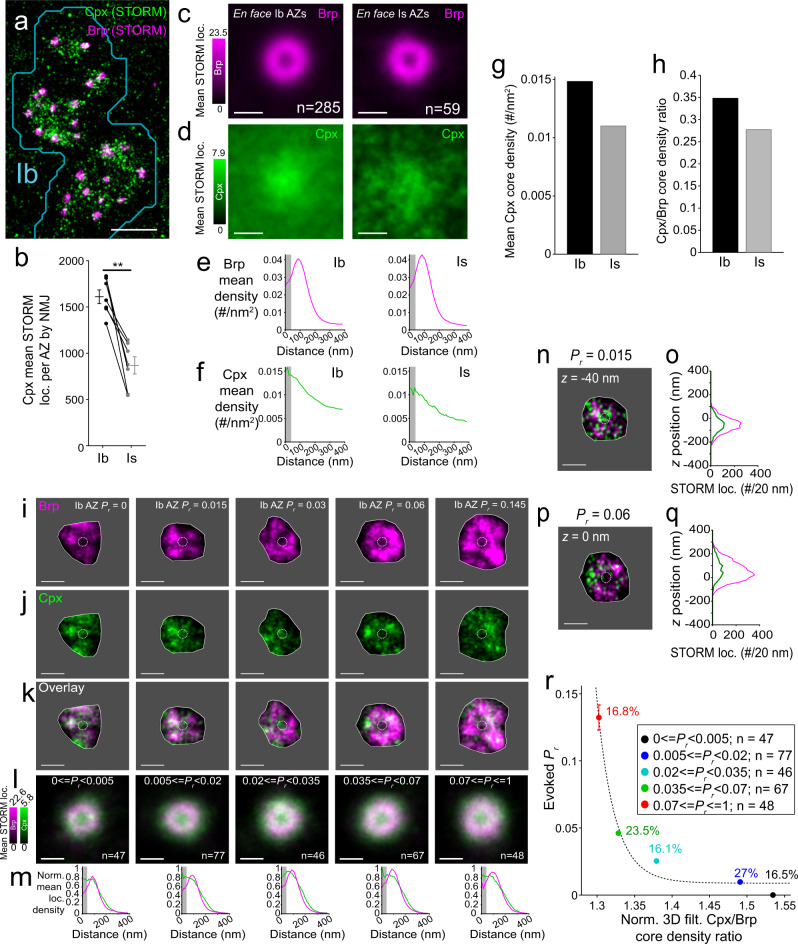
Cpx at the Brp annular AZ core suppresses evoked transmission. **a** Brp and Cpx 3D-STORM *z*-projection of the terminal boutons for a WT Ib axon. **b** Axon-specific differences in Cpx levels. Pooled mean total Cpx localizations for WT Ib and Is AZs (*n* = 7 NMJ pairs; Wilcoxon two-tailed signed-rank test *p* = 0.0156; mean ± SEM). **c**, **d** Cpx distributions in WT Ib (left; *n* = 285 AZs from 7 NMJs) and Is (right; *n* = 59 AZs from 7 NMJs) *en face*-aligned AZs showing Brp (**c**; magenta) and Cpx (**d**; green) *z*-projection aligned mean density images. **e**, **f** Radial STORM Brp (**e**) and Cpx (**f**) density profiles for WT Ib (left) and Is (right) *en face*-aligned AZs in **c**,**d** with core 40 nm radial region (gray area) indicated. **g**, **h** Cpx enrichment in Ib AZ core. Mean core radial Cpx density (**g**) and core Cpx/Brp density ratio (**h**) in WT AZs. **i**-**k**, Representative, *P*_*r*_-binned, volume filtered, *en face* Ib AZ 3D-STORM images (all from the same NMJ) for Brp (**i**; magenta), Cpx (**j**; green), and overlay (**k**). Largest *x*-*y* quantification area for each AZ (gray area) and core 40 nm radius (dotted circle) indicated. **l** *P*_*r*_-binned, aligned and averaged 3D-STORM overlays showing 3D-filtered, average density *z*-projections for WT Ib *en face* AZs. **m** Radial normalized mean 3D-STORM localization density profiles for *P*_*r*_-binned spatial averages in **l** with core 40 nm radial region (gray area) indicated. **n**–**q** AZ core Cpx levels in low *P*_*r*_ (**n**, **o**) and high *P*_*r*_ AZs (**p**, **q**) including a 3D-filtered, 20-nm-thick *z* slice image at indicated depth (**n**, **p**) and the corresponding STORM localization counts along with the *z* depth of the AZ (**o**, **q**). The core 40 nm radial region (dotted circle) is also indicated (**n**, **p**). **r** AZ core Cpx/Brp ratio inversely related to *P*_*r*_. *P*_*r*_-binned, 3D-filtered, mean core 40 nm radial Cpx/Brp density ratio (*R*^2^ = 0.98 *y* = 2.4 × 10^5^*x*^−55^ + 0.009; mean ± SEM). Scale bars: 2 µm (**a**) and 200 nm (**c**, **d**, **i**–**l**, **n**, **p**).

Our observation that Brp and Cac account for only part of *P*_*r*_ diversity suggested the involvement of other regulators of evoked release and the suppression of spontaneous transmission at the site of maximal evoked transmission suggested that one of these players could be a protein that inhibits spontaneous release. We, therefore, turned to Complexin (Cpx), which affects both modes of release across species, and strongly inhibits spontaneous release in *Drosophila*^50^. Cpx was found to be broadly distributed in both the Ib and Is axon (Suppl. Fig. 11) and enriched at the AZ, with Brp-associated Cpx levels 2–3 fold higher at Ib AZs relative to the AZs of Is axons that co-innervated the muscle (Fig. 5a, b and Suppl. Fig. 12). The widespread distribution throughout the bouton is consistent with partitioning of prenylated Cpx into endomembranes, including synaptic vesicles^66–69^, which are more abundant in Ib boutons^70^. Analysis of 3D-STORM spatially averaged *en face* AZs, showed that Cpx density was maximal at the Brp annular center (Fig. 5c–f), where Cac is concentrated (Figs. 2 and 3) and where electron tomography has shown that synaptic vesicles dock^71^.

To determine the contribution of Cpx to transmission diversity, we performed QuaSOR imaging in conjunction with Brp/Cpx 3D-STORM. We took advantage of the 3D-STORM to focus on Cpx in the AZ by counting Cpx localizations within ~80 nm of the 3D distribution of Brp. Although Cpx acts as a brake on spontaneous fusion, we found that Cpx localizations in the AZ were weakly but positively correlated with *F*_*s*_ (Suppl. Fig. 13a). This may be accounted for by the association of Cpx with synaptic vesicles^66,67^ and evidence that Cpx links vesicles to Brp^69^. Indeed, Cpx localizations were linearly related to Brp localizations (Suppl. Fig. 13b). Consistent with this observation, *P*_*r*_ increased supra-linearly with Cpx localizations (Suppl. Fig. 13c), as did *P*_*r*_ with Brp (Fig. 3g).

In an attempt to further isolate the effect of Cpx on the release machinery, we wanted to focus on the fraction of molecules that most likely interact with SNAREs. We, therefore, further analyzed Cpx molecules located within the core 40 nm radius of the Brp annulus of *en face*-oriented AZs, the location where AP-evoked fusion most likely takes place^72^. Cpx in the AZ core was ~40% higher in Ib synapses compared to Is synapses (Fig. 5g), as was the Cpx/Brp ratio (Fig. 5h), suggesting that excess Cpx in the SNARE complex may inhibit evoked release.

We next asked whether differences in Cpx content of the AZ core could account for *P*_*r*_ differences between Ib synapses of the same axon (Fig. 5i–m). As seen in our experiments on Brp and Cac, where we measured total Brp at the AZ (Fig. 3g), Brp in the AZ core increased with *P*_*r*_ (Suppl. Fig. 13d). In contrast, Cpx in the AZ core was relatively constant across the wide range of *P*_*r*_ values (Suppl. Fig. 13d). In these *en face*-oriented AZs, both in the *x*-*y* plane (Fig. 5m) and along the *z*-axis (Fig. 5n–q), the core Cpx/Brp ratio was highest in the weakest synapses (Fig. 5r).

Together, these results suggest that Cpx exerts two opposing effects on transmitter release, neither of which would be apparent with previous methodologies. On one hand, bulk Cpx appears to promote both spontaneous and evoked release, perhaps by interacting with Brp and synaptic vesicles near the release machinery. On the other hand, Cpx in the Brp annular core, where vesicles dock and fuse, appears to inhibit both spontaneous and evoked release. The spatial disconnect between spontaneous and evoked release (Fig. 4a–d) implies that the inhibition of spontaneous release is more potent, with complete suppression at the site of maximal evoked release where Cpx density is highest. We tested this notion next by knocking down Cpx.

### Complexin suppresses both spontaneous and evoked transmission

**Fig. 6:**
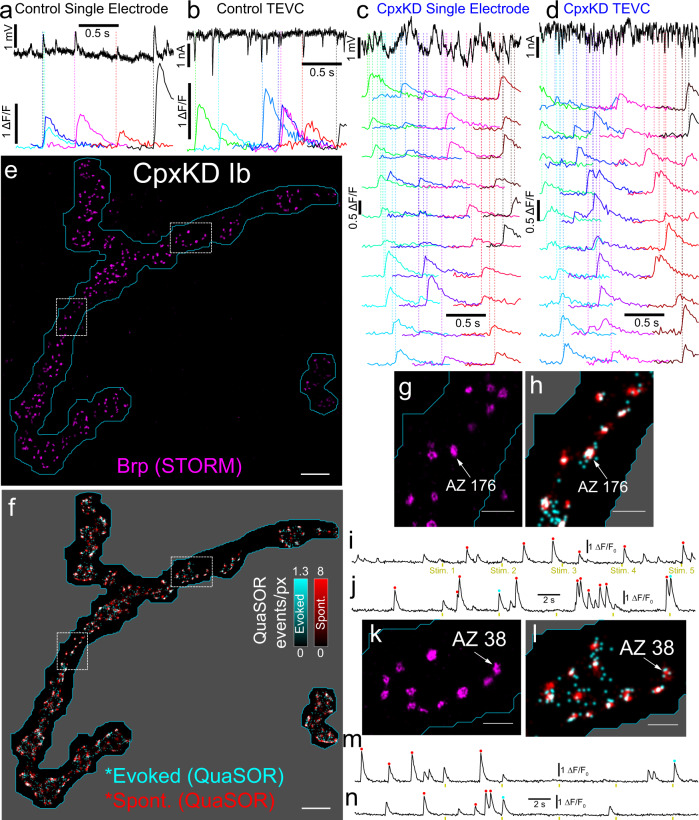
Analysis of spontaneous and evoked transmission in the knockdown of Cpx. **a**–**d** Simultaneous electrophysiological and optical analysis of transmission in Control (**a**, **b**) and CpxKD (**c**, **d**) NMJs. Spontaneous events are infrequent enough in control animals to be easily measured electrophysiologically (black traces) as mEPSPs (**a**) and mEPSCs (**b**) and to identify corresponding optical events at identified synapses (colored traces). In CpxKD animals, the frequency of spontaneous mEPSPs (**c**) and mEPSCs **(d**) (black traces) is too high to measure electrophysiological events accurately, but optical events at individual synapses occur infrequently enough to measure easily (colored traces). **e** Brp 3D-STORM *z*-projection image for a CpxKD Ib NMJ (OK6-Gal4, UAS-Cpx^RNAi^, SynapGCaMP6f). **f** CpxKD QuaSOR map including evoked (cyan) and spontaneous (red) events (*σ* = 42.3 nm) aligned to 3D-STORM area in **e**. **g**–**n** Two magnified Ib boutons from ROIs shown in dashed squares in **f**, each with a single example AZ (**g**, **h**, AZ 176 and **k**, **l**, AZ 38) and its corresponding QuaSOR traces (**i**, **j** for AZ 176 and **m**, **n** for AZ 38). The QuaSOR traces mark evoked (cyan dots) and spontaneous (red dots) transmission events. Unmarked events were generated at other neighboring sites. Stimulus times are indicated below the traces. Scale bars: 5 µm (**e**, **f**), 1 µm (**g**, **h**, **k**, **l**).

To test the model that Cpx suppresses both evoked and spontaneous release and causes their spatial mismatch, we knocked-down Cpx in the motor neurons by expression of a Cpx^RNAi^ construct (UAS-Cpx^RNAi^ driven by the motor neuron-specific OK6-Gal4; “CpxKD”) (Suppl. Fig. 11). We imaged these NMJs continuously while stimulating the motor nerve electrically at 0.2 Hz (Fig. 6 and Suppl. Fig. 16b, c, e, f). As expected, given the known role of Cpx in *Drosophila*, there was a large increase in spontaneous transmission in CpxKD NMJs (Fig. 6a–d; Suppl. Figs. 16a, d and 17c, d; and Suppl. Video 3). AP-evoked AZ participation (Suppl. Fig. 17a–b) was similar to that seen in WT Ib NMJs (Suppl. Fig. 7b). However, in the highly spontaneously active CpxKD Ib NMJs, ~7% of AZs had a spontaneous transmission event every second (Suppl. Fig. 17d), considerably above the frequency seen in control animals (Suppl. Fig. 7d). Alignment of spontaneous and evoked events with 3D-STORM (Fig. 6g–n and Suppl. Video 4) or 3D-Airyscan maps of Brp location provided a robust 1–1 mapping of synaptic properties.

**Fig. 7:**
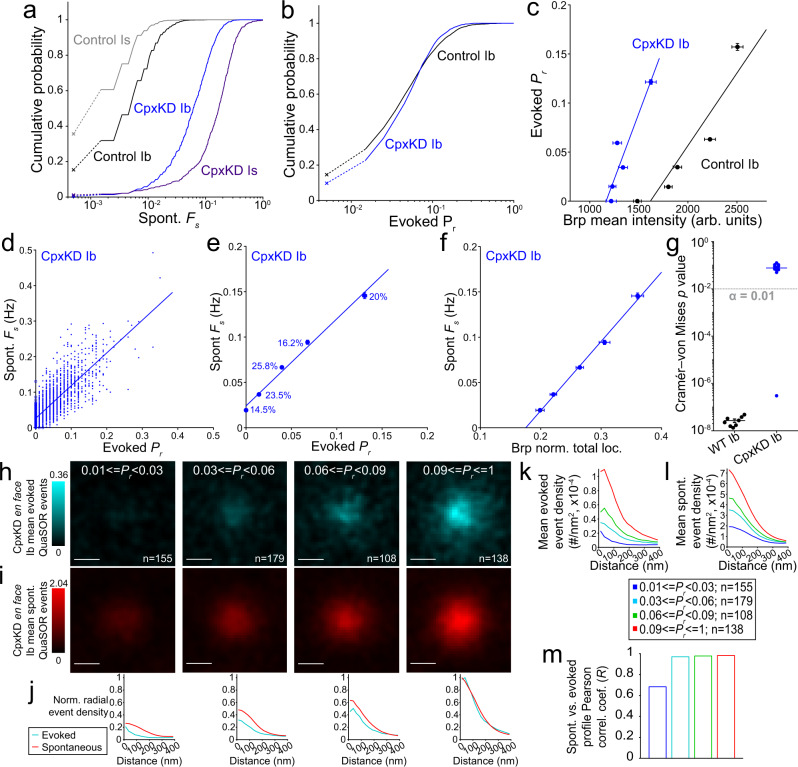
Cpx knockdown enhances dependence of evoked transmission on Brp and eliminates mismatch between spontaneous and evoked transmission. **a** CpxKD increases *F*_*s*_ in Ib and Is. *F*_*s*_ distributions for Control (OK6-Gal4, attP40^Empty^, SynapGCaMP6f) Ib (*n* = 2709 AZs, 7 NMJs), Control Is (*n* = 1114 AZs, 7 NMJs), CpxKD Ib (*n* = 1547 AZs, 5 NMJs), and CpxKD Is (*n* = 697 AZs, 5 NMJs) QuaSOR-Airyscan matched AZs (One-way ANOVA *p* = 0; Tukey–Kramer post hoc test; Control Ib vs Control Is *p* = 0.15; CpxKD Ib vs CpxKD Is *p* = 3.8 × 10^−9^; Control Ib vs CpxKD Ib *p* < 3.8 × 10^−9^; Control Is vs CpxKD Is *p* < 3.8 × 10^−9^). **b** AP-evoked release in CpxKD Ib NMJs. *P*_*r*_ distributions for QuaSOR-Airyscan matched Control and CpxKD Ib AZs (two-sample two-sided Kolmogorov–Smirnov test *p* = 0.00071). **c** *P*_*r*_ dependence on Brp shifts left in CpxKD Ib. *P*_*r*_-binned, mean Airyscan Brp voxel intensities versus mean evoked *P*_*r*_ for Control Ib (*y* = 1.48 × 10^−4^*x*−0.239; *R*^2^ = 0.873) and CpxKD Ib (*y* = 2.68 × 10^−4^*x*−0.313; *R*^2^ = 0.871) AZs. **d**, **e** *P*_*r*_ and *F*_*s*_ highly correlated in CpxKD Ib (mean ± SEM). QuaSOR-STORM matched *P*_*r*_ versus *F*_*s*_ for CpxKD Ib AZs (**d**) (*y* = 0.92*x* + 0.026; *R*^2^ = 0.62; *n* = 1774 AZs, 6 NMJs) and *P*_*r*_-binned (bin percentages indicated) mean *P*_*r*_ versus mean *F*_*s*_ for CpxKD Ib AZs in **e** (*R*^2^ = 0.991; *y* = 0.96*x* + 0.024; mean ± SEM) (**e**). **f** Dependence of *F*_*s*_ on Brp in CpxKD Ib NMJs. QuaSOR-STORM matched, *P*_*r*_-binned, mean NMJ-normalized total Brp STORM localizations versus *F*_*s*_ (*R*^2^ = 0.995; *y* = 0.76x–0.13; mean ± SEM). **g** Global spatial overlap between spontaneous and evoked events in CpxKD but not WT. Pooled two-sample two-sided Cramér–von Mises test *p* values for WT (*n* = 9) and CpxKD Ib (*n* = 11) NMJs; 100 stimuli (evoked) and 600 s without stimulation (spontaneous) (mean ± SEM). **h**, **i** Local spatial overlap between spontaneous and evoked events in CpxKD animals. *P*_*r*_-binned, aligned evoked (**h**) and spontaneous (**i**) QuaSOR spatial averages (*σ* = 42.3 nm) from *en face* evoked-active CpxKD Ib AZs (*n* = 655, 6 NMJs). **j** Normalized radial density profiles for CpxKD Ib AZs. **k**, **l** Aligned *P*_*r*_-binned, *en face* classified, evoked-active CpxKD Ib AZ radial mean QuaSOR evoked (**k**) and spontaneous (**l**) density profiles. **m** High local correlation between spontaneous and evoked radial profiles. Pearson correlation coefficients (*R*) for *P*_*r*_-binned, aligned, Ib *en face* classified, radial mean density profiles. Scale bars: 200 nm (**h**, **i**).

This analysis was made possible by our ability to detect individual events because of their spatial segregation to different synapses. Electrophysiological measurement of transmission events in Cpx mutant animals is made difficult by the high frequency of spontaneous release, which is summed for the hundreds of synapses of the NMJ, and by the blending of transmission from Ib and Is synapses (Fig. 6c, d, black traces). In contrast, quantal imaging of transmission followed by QuaSOR analysis makes it possible to study Ib synapses one by one, where individual spontaneous transmission events are well separated in time, and in isolation from events at Is synapses. We recorded mEPSPs and EPSPs (bridge mode) or mEPSCs and EPSCs (two-electrode voltage-clamp mode) simultaneously with imaging of quantal transmission, followed by QuaSOR analysis to compare WT to CpxKD. While, compared to WT, aggregate spontaneous transmission measured electrophysiologically in the CpxKD, occurred at a high frequency which made it difficult to distinguish and measure individual events (Fig. 6a–d, black traces; Suppl. Fig. 14a–d), transmission at individual sites measured optically was low enough (Fig. 6g–n) to allow individual transmission events to be readily resolved (Fig. 6c, d, color traces). We observed that average *F*_*s*_ was 10.7-fold higher at Ib synapses and 65.8-fold higher at Is synapses (Fig. 7a and Suppl. Fig. 18a). The number of synapses with no detected spontaneous events declined to almost zero in both Ib and Is axons of CpxKD animals (Suppl. Fig. 18b).

In contrast to the large increase in spontaneous transmission in the CpxKD, evoked quantal output (optical quantal density) and *P*_*r*_ at Ib synapses were similar to control (Fig. 7b and Suppl. Fig. 18c, d). We find that, compared to control, the CpxKD has a smaller amplitude EPSC (Suppl. Fig. 14e, f). We wondered if this were due to a reduction in the number of synapses, quantal size or *P*_*r*_. Our analysis revealed no effect of the CpxKD at either Ib or Is synapses on either AZ density (Suppl. Fig. 18e) or *P*_*r*_ (Fig. 7b and Suppl. Fig. 18d). However, the CpxKD had smaller amplitude spontaneous single synapse optical events at both Ib and Is synapses (Suppl. Fig. 15). Thus, reduced quantal size appears to contribute to the reduction in EPSC amplitude in the CpxKD.

Therefore, the lack of an effect on *P*_*r*_, in light of the reduction in levels of the release-promoting Brp, suggested that knockdown of Cpx had an offsetting effect, i.e. that Cpx generally suppresses evoked release. We tested this idea by examining the effect of the CpxKD on the dependence of *P*_*r*_ on Brp. Strikingly, the *P*_*r*_-Brp relation shifted to the left in the CpxKD Ib NMJ (Fig. 7c), yielding higher *P*_*r*_ for the same Brp levels. This also meant that AZs with low Brp, which had no detected evoked transmission in control animals, were active in CpxKD animals. Thus, Cpx appears to suppress evoked release and this inhibitory effect in Cpx mutants and KDs may have not been recognized earlier because of a compensatory reduction in the Brp content of AZs.

### Knockdown of Complexin eliminates the mismatch between the spontaneous and evoked transmission

We wondered whether knockdown of Cpx would eliminate the quantitative mismatch between AP-evoked and spontaneous release. We found that the very shallow relation between *F*_*s*_ and *P*_*r*_ in WT and *rab3* mutant NMJs (Suppl. Figs. 9b, 10e and 19c) was increased to near unity in slope in CpxKD animals (Fig. 7d, e and Suppl. Fig. 19c). Moreover, the very weak dependence of *F*_*s*_ on Brp in WT and control Ib synapses (Suppl. Figs. 9d and 19d) increased greatly in the CpxKD (Fig. 7f and Suppl. Fig. 19d). Together, these observations suggest that, in the absence of Cpx, the AZ scaffold functions as a common, powerful determinant of both spontaneous and evoked release.

Strikingly, we found that knock-down of Cpx eliminated the spatial mismatch between transmission modes. The suppression of spontaneous transmission at the site of maximal evoked transmission disappeared, yielding similar spatial profiles in the CpxKD across *P*_*r*_ for *en face*-oriented AZs (Fig. 7h–j) and the highly significant difference between the spatial distribution of spontaneous and evoked QuaSOR events of WT Ib NMJs (Fig. 4e) gave way to statistically indistinguishable global spatial distributions of spontaneous and evoked QuaSOR events in CpxKD Ib NMJs (Fig. 7k–m). Thus, Cpx appears to suppress both spontaneous and evoked release but to more strongly suppress spontaneous release, and this unequal suppression results in a quantitative and spatial mismatch between spontaneous and evoked transmission within the wildtype AZ.

## Discussion

To understand the mechanisms that regulate synaptic strength and generate synapse diversity, we set out to develop a new set of super-resolution imaging tools that together would allow us to relate quantal transmission to presynaptic molecular composition in an intact model synapse. Imaging of Ca^2+^ influx through ionotropic glutamate receptors, with a postsynaptically targeted reporter, provided a quantal-resolution proxy for the EPSC, and QuaSOR analysis increased spatial resolution sufficiently to resolve synapses even in dense areas of the *Drosophila* NMJ. QuaSOR makes it possible to determine *P*_*r*_ directly by failure analysis under physiological Ca^2+^, i.e. at physiological *P*_*r*_, avoiding reliance on estimation based on the ratio between evoked and spontaneous EPSC amplitudes (problematic in view of our finding that the sites of evoked and spontaneous transmission are segregated within the synapse), fits of amplitude distributions or analysis of variance^73,74^. Post-hoc super-resolution presynaptic axon reconstructions enabled us to correlate transmission to the molecular composition and nano-architecture of the presynaptic AZ for thousands of synapses.

Earlier work suggested that, despite a common history of activity and postsynaptic target, transmission varies greatly between the synapses of a single Ib motor axon^28–31,33,34^. QuaSOR assignment of transmission events to identified synapses showed this to be the case across thousands of synapses and revealed that the heterogeneity is even greater than previously thought, with *P*_*r*_ ranging over at least 100-fold, from <0.005 to 0.6. Half of the synapses are very weak (*P*_*r*_ < 0.02) and AP-evoked transmission is dominated by a small fraction of higher *P*_*r*_ synapses during low levels of activity. This large pool of low-*P*_*r*_ synapses could operate as a reserve that would be recruited to sustain transmission during long, high-frequency AP bursts, such as occur during locomotion^28,75^.

Previous studies at the NMJ demonstrated a positive relationship between *P*_*r*_ and both Cac^29,30^ and Brp^32–34^. The ability to relate quantal transmission to multi-color 3D-STORM clarifies the nature of this relationship, by showing that *P*_*r*_ increases with the ~5^th^ power of Cac, both in wildtype synapses and in synapses of a *rab3* mutant whose AZs are enlarged, consistent with the power-dependence of release on Ca^2+^ ^43,44,76,77^. Cac and Brp levels were also correlated with one another, consistent with Brp recruiting Cac to the AZ^46^. Although they are strong determinants, Cac and Brp only account for a fraction of the variance of *P*_*r*_, indicating that other factors are at play. When AZs were expanded by the *rab3* mutant to include more Brp and Cac, *P*_*r*_ increased to higher values, while maintaining the shallow *F*_*s*_-*P*_*r*_ relation, the displacement of spontaneous transmission to locations outside the sites of evoked and the high power dependence of *P*_*r*_ on Cac. These observations are consistent with a mechanism that tunes *P*_*r*_ by regulating the size of the Brp scaffold and the number of Cac channels.

In considering other potential regulators of presynaptic strength, we remarked on an almost complete lack of correspondence between evoked and spontaneous transmission in WT animals. Most startlingly was a complete suppression of spontaneous transmission at the site of maximal evoked transmission. This segregation is only possible to detect with these analysis tools and agrees with evidence from the use-dependent block that spontaneous and evoked release activate distinct populations of glutamate receptors in hippocampal neurons^78^ and the *Drosophila* NMJ^33^. Our observations reveal that this separation arises not only from synapse specialization, as proposed in earlier studies but from physical segregation of evoked and spontaneous transmission *within* the synapse. This spatial mismatch is remarkably consistent with recent iGluSnFR mapping of spontaneous and evoked transmission events in cultured hippocampal synapses^40^, suggesting that segregation of transmission modes within the synapse may be a general phenomenon.

We considered that a factor that regulates both spontaneous and evoked release could be responsible for their spatial mismatch. We turned to Cpx, which has been shown to regulate both spontaneous and evoked release in complicated and contradictory ways^50^. In vitro, Cpx interacts with the coiled-coiled domains of the SNARE complex to inhibit fusion and is displaced by Ca^2+^-bound synaptotagmin to trigger AP-evoked release^79–81^. The mammalian isoforms of Cpx contain both fusogenic and inhibitory domains^50,82^. Pan-neuronal removal of Cpx in *Drosophila* reduces postsynaptic response amplitude, suggesting that Cpx promotes evoked release^49,82–84^. In contrast, expression of *Drosophila* Cpx in mammalian neurons suppresses evoked release^82^. Cpx may also adjust the relationship between release and internal Ca^2+^ concentration through its role as an adapter that helps determine the composition of the release apparatus^16,18,50^. We find that Cpx is broadly distributed in the axon, enriched at the AZ and most densely concentrated in the Brp annular core. As the Cpx/Brp ratio within the AZ core rises, the *P*_*r*_ of Ib synapses decreases. This suggests that Cpx in the AZ core, which is positioned to interact with SNARE complexes, inhibits evoked release. Consistent with this relationship, Cpx knockdown increases the dependence of *P*_*r*_ on Brp so that at equivalent Brp levels *P*_*r*_ is higher when Cpx is knocked down and low Brp synapses with no detected transmission events become active.

Knockdown of Cpx increased *F*_*s*_ by ~11-fold at Ib synapses and ~66-fold at Is synapses, indicating that Cpx suppresses spontaneous transmission more strongly than evoked transmission. In light of this and of our findings that: (a) Cpx density is highest in the Brp annular core, where Cac is also located, and where AP-evoked vesicle fusion is therefore expected to take place, (b) spontaneous transmission is suppressed at the site of maximal evoked transmission, (c) spontaneous and evoked transmission are poorly correlated, and (d) knockdown of Cpx eliminates the spatial and quantitative mismatch between spontaneous and evoked transmission, we propose that Cpx within the AZ core partly suppresses evoked release and completely suppresses spontaneous release. This differential suppression can preserve vesicles that are docked near Ca^2+^ channels in a state that is ready for release when the AP arrives, yielding a higher signal-to-noise for AP-evoked transmission over background spontaneous transmission.

It is striking how knockdown of Cpx converts the relationship between *P*_*r*_ and *F*_*s*_ to near 1:1 and the spatial relationship of spontaneous and evoked transmission to coincident. This suggests that spontaneous and evoked release rates are, after all, governed by common factors. Brp levels were reduced in the CpxKD, possibly reflecting a compensatory mechanism that keeps the *P*_*r*_ of Ib synapses at near WT levels, as shown in recent focal extracellular recordings from Ib boutons^85^. While Cpx in the Brp annular core suppresses *P*_*r*_, we find that higher bulk Cpx around the AZ is associated with higher *P*_*r*_. This bulk Cpx likely reflects prenylated Cpx that is associated with endosomes and synaptic vesicles^66,67^, which links vesicles to Brp^69^, and so may reflect higher vesicle content.

Together, QuaSOR and super-resolution molecular imaging of AZs reveals that the balance between the quantity and nanoscale localizations of Cac, Brp, and Cpx contribute to a wide diversity in release dynamics for synapses that otherwise share common pre-post pairing and activity history. This heterogeneity could serve to maintain a deep pool of reserve synapses upon which the system can draw under diverse physiological demands.

## Methods

### *Drosophila* husbandry and genetics

Several flies were obtained from the Bloomington Drosophila Stock Center (BDSC) including; attP40^Empty^ (BDSC Line 36304), UAS-Cpx^RNAi^ (pVALIUM20 vector; inserted into attP40; BDSC Line 42017), UAS-Cpx (BDSC Line 39743), UAS-Cac^RNAi^ (pVALIUM10 vector; inserted into attP2; BDSC Line 27244), and UAS-Dcr2 (BDSC Line 24648). OK6-Gal4^86^ and SynapGCaMP6f (3rd chromosome MHC-CD8-GCaMP6f-Sh) lines were reported previously^28,33^. Flies were raised on standard corn meal and molasses media at 25 °C. Female wandering third instar larvae were used in all experiments. Only actively crawling larvae were used for experiments. When required, third instar larvae were screened using a Zeiss Axio Zoom.V16 microscope (Carl Zeiss, Inc. Oberkochen, Germany) through the use of balancers with larval markers including CyO^GFP^ (3xP3-EGFP variant) and TM6B. All larvae contained a single copy of SynapGCaMP6f on the 3rd chromosome. The following genotypes were used: WT (*w*^*1118*^; +/+; SynapGCaMP6f/+), Control (*w*^*1118*^; OK6-Gal4/attP40^Empty^; SynapGCaMP6f/+), CpxKD (*w*^*1118*^; OK6-Gal4/UAS-Cpx^RNAi^; SynapGCaMP6f/+), CpxOE (*w*^*1118*^; OK6-Gal4/+; UAS-Cpx/SynapGCaMP6f), CacKD (UAS-Dcr2/*w*^*1118*^; OK6-Gal4/+; UAS-Cac^RNAi^/SynapGCaMP6f), and *rab3*^*rup*^ (*w*^*1118*^; *rab3*^*rup*^/DF(2R)ED2076; SynapGCaMP6f/+).

### SynapGCaMP6f optical quantal imaging

Optical quantal imaging was performed similarly to our previous report^28^. Briefly, third instar larvae were dissected on PDMS (Sylgard 184, Dow Corning, Auburn, MI) pads in ice-cold HL3 solution containing, in mM: 70 NaCl, 5 KCl, 0.45 CaCl_2_ ∙ 2H_2_O, 20 MgCl_2_ ∙ 6H_2_O, 10 NaHCO_3_, 5 trehalose, 115 sucrose, 5 HEPES, and with pH adjusted to 7.2. Following removal of the brain, larval fillets were washed and imaged in room temperature HL3 containing 1.5 mM Ca^2+^ and 25 mM Mg^2+^. Fluorescence images were acquired at room temperature with a Vivo Spinning Disk Confocal microscope (3i Intelligent Imaging Innovations, Denver, CO), using a 63 × 1.0NA water immersion objective (Zeiss), 1.2X optical adapter, LaserStack 488 nm (50 mW) laser, CSU-X1 A1 spinning disk (Yokogawa Tokyo, Japan), standard GFP filter, and EMCCD camera (Photometrics Evolve512, Tucson, AZ). All live SynapGCaMP6f imaging recordings were done on ventral longitudinal abdominal muscle 4 at segments A3-A5 of third instar larvae. All imaging was performed using 50 ms exposures (20 fps) of the full camera sensor (512 × 512 px) with the exception of Suppl. Fig. 1a–f, which was acquired with 20 ms exposures (50 fps).

Nerve stimulation was performed with a suction electrode attached to a Stimulus Isolation Unit (SIU, ISO-Flex, A.M.P.I Jerusalem, Israel) with 100 μs stimulus duration. Stimulation intensity was adjusted to recruit both Ib and Is axons, as verified during the imaging. Nerve stimulation and imaging were synchronized using custom-written MATLAB scripts (MATLAB Version 2015b, MathWorks, Inc., Natick, MA) in order to control the SIU and trigger imaging episodes within SlideBook (v6.0.16, 3i Intelligent Imaging Innovations).

To gather spontaneous and evoked transmission events at each NMJ, two separate quantal imaging experimental protocols were utilized. In the sequential protocol, we collected AP-evoked responses during short, single-stimulus episodes. Here each stimulus was collected during a series of 10 images (50 ms exposures). Each episode had 3–4 baseline frames prior to nerve stimulation. We collected a minimum of 200 single-stimulus episode trials at 0.2 Hz. This was followed immediately by spontaneous event collection, imaging continuously at 20 FPS (50 ms exposures in streaming capture mode) for 2 minutes total separated into four, 30 s movies. These brief pauses between movies allowed for the manual correction of any drift in the NMJ. In the second interleaved activity imaging protocol, both spontaneous and evoked events were collected during the same image acquisition protocol. This was done by imaging continuously at 20 FPS (50 ms exposures in streaming capture mode) for 30 s while stimulating the nerve 5 times with 5 s intervals during each 30 s movie. Minor focusing adjustments were made between movies with a maximum of 10 s between movies. A minimum of 20 movies were captured per NMJ for an overall protocol of 600 s of imaging with 100 stimuli.

To ensure comparability between experiments, recordings were done on only one NMJ per larva (i.e. the number of NMJs = the number of larvae) in recordings that were performed within 30 min of the beginning of the dissection, thereby ensuring health and similar conditions. To determine whether we could pool results from segments A3, A4, and A5, we analyzed recordings from WT animals from A3 (*n* = 9), A4 (*n* = 11), and A5 (*n* = 13) and compared their properties (Suppl. Table 1), we found no significant difference between Ib synapses in the three segments in the following key parameters: (a) Quantal density, (b) Average Pr, (c) Brp STORM localizations per AZ, and (d) normalized Brp per AZ (Suppl. Fig. 20).

### SynapGCaMP6f registration and bleach correction

The initial quantal image analysis was performed using custom-written MATLAB routines similar to the previous work^28^. In the case of the episodic evoked imaging protocol, individual stimulus episodes were excluded due to out of focus NMJs, moving NMJs or failed axon recruitment. Otherwise, all movies were filtered (Gaussian low-pass filter), to reduce high-frequency noise. Image analysis areas were then separated into Ib and Is NMJ regions according to their baseline SynapGCaMP6f fluorescence. All imaging data were registered using a multi-stage approach, during which all images were registered to a common reference image, even when multiple imaging modes were applied. This was either the first image of the first episode for the episodic imaging protocol, or an average image of the first 20–30 frames of the first movie for spontaneous-only and interleaved spontaneous/evoked protocols. The latter method was required due to the inclusion of the highly spontaneously active CpxKD animals in which, due to the high rates of spontaneous release, there are often no single frames without at least one spontaneous event in the movie.

Following area selection and reference image selection, NMJs were tracked relative to this reference image using a rigid subpixel registration method to remove any large movements within the NMJ imaging area^28,87^. To correct for local bouton movements and small NMJ shape changes, we used a custom diffeomorphic implementation of a demons algorithm^88^, similar to our previous in vivo image registration^28^. Here we enhanced the contrast of the NMJ and calculated a displacement field for each image pair (each image relative to the reference image). This method can generate localized movement artifacts, so we applied a temporal filter for each pixel’s displacement vector over time, prior to applying the respective displacement field to each image. This ensured smooth tracking of each bouton over long imaging times. In the case of episodic evoked imaging protocols, only the first image of each 10 image-single stimulus movie was used to calculate the corrections for the whole episode. For all 30 s continuous imaging datasets, registration transformations were calculated on every image of the movie relative to a common reference image.

Once motion corrected, movies were bleach corrected using two methods. In the case of the episodic AP-evoked imaging protocols, we fit a double exponential bleach correction curve to the mean baseline pre- and post-stimulus fluorescence data for each trial separately. Spontaneous or interleaved spontaneous/evoked movies were bleach corrected by adjusting the images according to a moving average (across 20 frames) of the mean fluorescence values for the whole NMJ analysis region. Following bleach correction, both ∆F and ∆F/F movies were generated using the first image as the baseline fluorescence (F_0_) image for the episodic data. An average minimum intensity image across the first 30 frames of each 30 s movie was used as the baseline image (F_0_) for the other imaging protocols. This method made it possible to eliminate the contamination of the ∆F or ∆F/F signals throughout the movie by spontaneous events present during the baseline images.

### Quantal event detection

We employed a template fitting approach to identify all quantal responses for both AP-evoked and spontaneous experiments. For episodic evoked imaging data, due to the synchronous nature of the evoked responses, a single ∆F response template was utilized according to the average temporal profile for all evoked responses at that NMJ. For spontaneous-only or evoked/spontaneous interleaved protocols, we employed a multiple ∆F response template fitting approach to accommodate responses that may not align perfectly to a single ∆F response template. Similar to our approach described previously^28^, each pixel’s temporal response was analyzed independently to determine if it had a high degree of correlation with the template response(s), as determined by the degree of cross-covariance. These highly correlated pixels and frames were then flagged as active if they had ΔF/F amplitudes that were above a minimum threshold (typically between 0.04 and 0.05 ΔF/F) and at least 1.5–2 times larger than the standard deviation of the values at that pixel. When multiple templates were utilized, the best fitting template was determined according to the maximum cross-covariance found between the data and each template. We also maintained a minimum allowable time between subsequent events at each pixel (100 ms) to prevent over-fitting. Co-active pixels were then grouped together into a single response field. This produced a single, isolated, maximal temporal projection image of the response’s spatial profile. To eliminate false positives, we applied size, shape, and amplitude thresholds to these response fields. For the spontaneous-only datasets, responses were further manually validated according to their shape and temporal profile. For the simultaneous spontaneous and evoked datasets, we used a common event detection process for both classes of events together. Following detection processing, events were sorted into the appropriate category using the timing of the response relative to the stimulus timing. Only a 200 ms window, following each AP, was used to assign evoked responses.

### Quantal synaptic optical reconstruction (QuaSOR)

After event detection, isolation, and verification, we then proceeded to analyze the 2D response profile of each event’s maximum ΔF/F spatial profile using the custom QuaSOR algorithm. We took all identified responses and first isolated small ROIs containing individual or small groups of partially overlapping response fields that corresponded to individual or small groups of events. These smaller ROIs were then subjected to independent 2D Gaussian mixture model fitting of the isolated ∆F/F spatial profiles. For each smaller ROI, we generated a large number of test models using a maximum likelihood Expectation-Maximization algorithm. This allowed us to test a wide number of Gaussian mixture model components (1–8) so that we could identify multiple quantal events per ROI. Each set of Gaussian mixture model components also had multiple test fit replicates (20–200) utilizing randomized starting conditions to ensure adequate coverage of the parameter space for each ROI. Each 2D Gaussian mixture model was then scored according to a number of features. This included the number of components (too many components were penalized to prevent over fitting, especially during spontaneous recordings), the distance between the peaks of each component (below a minimum distance was penalized), and the normalized 2D cross-correlation with the input ROI ΔF/F response spatial profile. We also used a large set of manually validated spontaneous quantal event, single component, 2D Gaussian fit models to define distributions for known single quantal peak amplitudes, 2D Gaussian variances, 2D Gaussian variance differences, and 2D Gaussian covariance components. These distributions of validated Gaussian profiles were used to score each test model to ensure that the fitting within each mixture model was generating appropriately sized and shaped 2D Gaussian profiles. Finally, the scores for each fit model were tabulated and the best mixture model was determined for each response ROI. This method provides >90% fitting accuracy relative to a manual fit assessment.

Following 2D Gaussian mixture model fitting for all response ROIs, all event functions were then remapped onto a common coordinate space and merged to define a single set of 2D Gaussian functions for each quantal response. The peak positions of each 2D Gaussian component were used to define event locations in a 21.2 nm x 21.2 nm pixel coordinate space. For visualization purposes, maps were generated by applying a normalized 2D Gaussian filter to each event coordinate prior to adding each event to the overall image. In this way each pixel contains an approximation of the event density at that location. The corresponding filtering parameters, as well as colorbars that indicate event counts per pixel, are indicated in the respective figures and/or figure legends. To facilitate AZ matching we sometimes reoriented the QuaSOR data by rotating and translating all the coordinates prior to re-rendering the maps to match the STORM or Airyscan data. In all images, gray masked areas indicate where NMJs were either rotated and/or isolated for analysis and display purposes. See a summary of the QuaSOR processing steps in Suppl. Video 3.

### QuaSOR quantification

Local QuaSOR synapse alignments were performed by identifying maximum evoked coordinate density positions for synaptic ROIs within a 350 nm radius. Maintaining their relative organization to nearby events, these QuaSOR event coordinates were averaged together with other synapses to generate a mean density image for synapse groupings. Images were rendered using the parameters indicated in the figure legends. In this way, the relative position of QuaSOR events was preserved, including between evoked event and spontaneous event datasets. Average QuaSOR density radial profiles were then calculated by binning local QuaSOR event counts into 10 nm annuli surrounding the center alignment position, with total counts being normalized to the area of each annulus to provide an average QuaSOR event density per radial bin. The synaptic clustering of QuaSOR coordinates was compared to coordinates derived from the pixel-maxima method in which each event was identified by maximal ∆F/F pixel location^28^. Evoked localizations within each method were summed to construct localization density maps for the entire NMJ so that the two methods could be compared (Suppl. Fig. 1k, m). Each map was fit with a 2D Gaussian model using a non-linear least-squares regression (Suppl. Fig. 1l, n) and used to calculate the average half-maximum evoked cluster area and average evoked event cluster full width at half-maximum (FWHM) (Suppl. Fig. 1k–n). We found the AP-evoked type Ib QuaSOR cluster FWHM to be 236.8 ± 5.6 nm, whereas the pixel-maxima FWHM was 464.6 ± 7.8 nm for the same NMJs (*n* = 45 NMJs) (Suppl. Fig. 1o). This represents an estimated 3.8-fold improvement in resolution by QuaSOR (Suppl. Fig. 1p).

On its own, in absence of molecular imaging of AZ locations, synapse assignment in QuaSOR is not certain and could merge transmission events from neighboring synapses in regions of high synapse density. We, therefore, focused our study on NMJs where QuaSOR maps were related to molecular imaging maps generated in either STORM or Airy. Given the full-width half-max of 237 nm for QuaSOR coordinate clustering (Suppl. Fig. 1n), we added a safety margin and excluded areas of the NMJ where the distance between molecularly mapped AZs was <350 nm. Linear uniform global transformations in the *x* and *y* axes were applied to adjust for stretch of the tissue that occurred after the live optical quantal imaging during fixation and mounting on slides under cover glass for molecular imaging, yielding images in which QuaSOR transmission sites could be matched to STORM AZs (Suppl. Fig. 6a–f). After these global corrections, QuaSOR-STORM correspondence had an average QuaSOR-STORM offset correction of 257.0 ± 9.5 nm (Suppl. Fig. 6i), with a matching success rate of ~85% (Suppl. Fig. 6h). This offset represents the average distance discrepancies of neighboring AZs when comparing the same AZ pairs between their QuaSOR and STORM locations. Therefore, the alignment and matching steps are highly accurate in preserving the relative organization of synapses throughout the NMJ. The spatial alignment between QuaSOR and STORM is further illustrated in several example boutons from different NMJs at high magnification in Suppl. Fig. 6g. It is worth noting that, some AZs contain more than one Cac or Brp cluster (Fig. 3a, i and Suppl. Fig. 5a). Although it is not known if these constitute more than one release site in the single AZ, their close proximity within the AZ meant that they could not be resolved with QuaSOR, so that each AZ was treated as a synaptic unit.

To assess the relative co-localization between different sets of QuaSOR coordinates, we adapted a technique for the analysis of ecological point pattern processes^65^. Briefly, nearest neighbor distances were calculated between all events and two comparisons were utilized: (1) evoked event locations released during the first half of a stimulus train were compared to those released during the second half of the train or (2) all evoked coordinates were compared to all spontaneous event coordinates. Using these nearest neighbor distances, an O-ring statistic was computed. This O-ring statistic used transformed distances between points as a proxy for event location. An empirical cumulative distribution function of transformed distances between points was then calculated and a two-sample two-sided Cramér–von Mises test was employed to examine differences in transformed distances between the comparison groups. Statistically significant differences in transformed distances between conditions likely indicated a distinct underlying point process for the coordinate sets.

Single synapse AP-evoked transmission probability (*P*_*r*_) was determined by dividing evoked event counts within the single AZ domain by the number of motor nerve stimuli (*P*_*r*_ = # stimulus-coupled optical events per synapse/# stimuli; ranging from 0 to 1). Spontaneous transmission frequency (*F*_*s*_) was calculated for the total imaging time (*F*_*s*_ = # spontaneous optical events per synapse during a period with no stimulation/imaging time; in Hz).

### Antibodies and immunohistochemistry

Larvae were fixed in room temperature Bouin’s fixative (Ricca Chemical Company, Arlington, TX) for 5 min, permeabilized in PBS with 0.1% Triton X100 (PBT) and blocked in PBS with 0.1% Triton X100, 5% normal goat serum, and 0.02% sodium azide (PBN). All antibody incubations were performed in PBN and all washes were performed in PBT. Mouse anti-Brp (nc82; Developmental Studies Hybridoma Bank, Iowa City, IA) was used at 1:100 for confocal and Airyscan imaging, while it was used at 1:1000 for STORM imaging. Rabbit anti-Cpx antibody^49^ was used at 1:2000. Chicken anti-GFP (Thermo Fisher A10262; Thermo Fisher Scientific Waltham, MA) was used at 1:1000 to label SynapGCaMP6f in fixed samples. Rabbit anti-Cac antibody^57^ was used at 1:1000. Alexa Fluor 647 (Jackson 123-605-021) and Cy3-conjugated goat anti-Hrp (Jackson 123-165-021; Jackson ImmunoResearch Laboratories, West Grove, PA) antibodies were used at 1:250. Alexa Fluor 405 goat anti-mouse (Thermo Fisher A31553), Alexa Fluor 488 goat anti-chicken (Thermo Fisher A11039), Alexa Fluor 488 goat anti-rabbit (Thermo Fisher A11008), Alexa Fluor 555 goat anti-mouse (Thermo Fisher A32727), Alexa Fluor 568 goat anti-rabbit (Thermo Fisher A11036), Alexa Fluor 647 goat anti-rabbit (Thermo Fisher A32733), and Alexa Fluor 647 goat anti-mouse (Thermo Fisher A21235) secondary antibodies were all used at 1:1000. For the labeling of the anti-Cac primary antibody, we used goat, anti-rabbit Biotin F(ab’)_2_ (Jackson 111-066-144) at 1:1000, followed by Streptavidin-647 (Thermo Fisher S32357) at 1:500^57^.

CF680 goat anti-mouse secondary antibody was conjugated in-house and used at 1:1000. Briefly, CF680 NHS ester (Biotium Inc. Fremont, CA) was dissolved at a concentration of 3 mM in anhydrous DMSO. In all, 1 μL of dye solution, 80 μL of a 1.25 mg/mL suspension of unlabeled goat anti-mouse IgG1 secondary antibody (Jackson 115-005-205), and 10 μL of 1 M sodium bicarbonate solution were mixed and allowed to react for 15 min at room temperature. The reaction mixture was added to an equilibrated NAP-5 column (Sigma GE17-0853-01; Sigma-Aldrich St. Louis, MO) and flushed with PBS. The dye conjugated antibody was collected from the first colored eluent fraction and a concentration of 0.12 mg/mL was determined with a NanoDrop spectrophotometer (Thermo Fisher).

Antibodies obtained from the Developmental Studies Hybridoma Bank were developed under the auspices of the National Institute of Child Health and Human Development of the National Institutes of Health and maintained by the Department of Biological Sciences of the University of Iowa, Iowa City, IA. We confirmed the specificity of the Cac antibody by comparing staining in control animals and Cac^RNAi^ (Suppl. Fig. 3a). We confirmed the specificity of the Cpx antibody by comparing staining in control animals with both Cpx^RNAi^ (Cpx KD) and Cpx over-expressing (Cpx OE) animals (Suppl. Fig. 11). With both Cac and Cpx, the RNAi knocked down staining to a large extent, although not completely, consistent with what is often observed with other RNAis. To obtain uniformity in antibody staining, we fixed and stained the different genotypes compared within an experiment simultaneously with the same reagents.

### Confocal and Airyscan imaging and analysis

Following antibody incubations and washes, larval fillets were mounted in Vectashield (H-1000; Vector Laboratories, Burlingame, CA) or Vectashield HardSet (H-1400). Confocal and Airyscan imaging were performed on either a Zeiss LSM 880 or Zeiss LSM 980 microscope. All samples were imaged with a ×63 oil immersion objective (NA 1.4, DIC; Zeiss) using Zen software (Zeiss Zen black 2.3 SP1). All imaging data were collected using identical imaging and processing parameters for a given experiment set. Unless otherwise noted all confocal and Airyscan images are displayed as Gaussian filtered maximum intensity projections that were generated using custom-written MATLAB routines.

Confocal imaging data were acquired as *z* stacks with images of 2048 by 2048 px, at 1.2x zoom, with *x-y* pixel size of 55 nm, axial *z* spacing of 368 nm, 1 µs pixel dwell and 2x pixel averaging. Different channel data were acquired using custom wavelength cutoffs. Alexa Fluor 405 was excited with a 405 nm laser and data were acquired from 410 to 455 nm with a pinhole of 56.7 nm. Alexa Fluor 488 was excited with a 488 nm laser and data were acquired from 493 to 539 nm with a pinhole of 67.7 nm. Alexa Fluor 568 was excited with a 561 laser and data were acquired from 565–620 nm with a pinhole of 77.0 nm. Alexa Fluor 647 was excited with a 647-nm laser and data were acquired from 648 to 713 nm with a pinhole of 67.7 nm. Channels were scanned sequentially with the exception of Alexa Fluor 488 and Alexa Fluor 647 where data were acquired simultaneously. Quantification of average ROI intensities was calculated on terminal sets of 2–3 boutons using maximum intensity *z* projection images and background corrected using an ROI over the muscle area and adjacent to the boutons.

Airyscan imaging data^64,89^ were acquired using a tiling strategy, whereby smaller volumes of each NMJ were acquired sequentially and stitched together. Brp reconstructions for matching to QuaSOR data were acquired on the LSM 880 system. Briefly, each imaging volume was acquired with an additional magnification of 12x with a 5 AU pinhole, 2 µs pixel dwell times, line averaging of 2, an *x-y* dimension of 1024 by 1024 px (processed to 1000 by 1000 px) at 11 nm/px, and axial *z* spacing of 159 nm. Each of the three-channel volumes (anti-Brp/Alexa 405, anti-GFP/Alexa 647, and anti-Hrp/Cy3) were scanned sequentially. Alexa 405 was excited with a 405-nm laser and data were acquired with a BP420-480-LP605 filter. Alexa 488 was excited with a 488-nm laser and data were acquired with a BP495-550 + LP570 filter. Cy3 was excited with a 561 laser and data were acquired with a BP495-550 + LP570 filter. Additional Brp intensity quantification data were acquired on an LSM 980 system with 4x magnification, a 5 AU pinhole, 0.66 µs pixel dwell times, an *x-y* dimension of 792 by 792 px (processed to 768 by 768 px) at 43 nm/px and axial *z* spacing of 160 nm. Here anti-GFP/Alexa 488 and anti-Brp/Alexa 555 were excited with 488 nm and 561 nm lasers respectively and data were acquired with an SP615 filter.

Airyscan processing of all channels and *z* slices was performed in Zen (Zeiss Zen Black v2.3 SP1) using super-resolution settings. We then sequentially stitched each 4D volume together in Fiji (NIH ImageJ Version 2.0.0-rc-43/1.52n) using pairwise stitching with linear blending^90^. Alignments were performed to maximum intensity pixels of Brp puncta in overlapping volumes. Regions outside of the tile borders are always indicated by gray coloring in the corresponding images.

AZ locations in stitched Airyscan datasets were calculated by masking the volumetric Brp data. Sites were initially identified using a local 3D Brp intensity maxima with a minimum distance of 150 nm from neighboring maxima. AZ identifications were manually validated and corrected when neighboring sites were misidentified. AZ-specific Brp voxel intensities were calculated by identifying 3D-connected voxels to each AZ maxima for isolated AZs. To avoid artifacts of stitching and bleaching of overlapping regions, pixel quantifications were collected using the original image pixel intensity information, prior to stitching, with intensity values calculated from 3D voxel AZ masks.

### STORM imaging and analysis

STORM imaging was performed on a homebuilt setup based on a modified Nikon Eclipse Ti-E inverted fluorescence microscope using a Nikon CFI Plan Apo λ ×100 oil immersion objective (NA 1.45), as previously described^91^. Briefly, dye-labeled samples were mounted with imaging buffer [5% (w/v) glucose, 140 mM cysteamine, 0.8 mg/mL glucose oxidase, and 40 µg/mL catalase in 1 M Tris-HCl, pH 7.5]^53,54^ and sealed with Cytoseal 60. Conventional epifluorescence imaging of 560 nm (anti-Hrp Cy3) and 488 nm (anti-GFP Alexa Fluor 488) dyes was performed immediately prior to STORM imaging using the appropriate laser and filter set and served as reference for alignment with QuaSOR data. Alexa Fluor 647 and CF680 dye molecules were photoswitched to the dark state and imaged using a 647-nm laser (MPB Communications, Montreal, CAN). All lasers were introduced through an optical fiber into the back focal plane of the microscope and onto the sample at intensities of ~2 kWcm^−2^. These lasers reached the sample at incident angles slightly smaller than the critical angle, thus illuminating a few micrometers into the sample. Weak (~0.1 Wcm^−2^) 405 nm laser illumination was used to further assist photoswitching of single molecules. The resultant single-molecule fluorescence was recorded with an EM-CCD (Andor iXon Ultra 897; Oxford Instruments Abingdon, United Kingdom) at 110 frames per second, for a total of ∼70,000 frames per image. A cylindrical lens (*f* = 1 m) was inserted into the imaging path to encode the depth (*z*) information into the single-molecule image shape^54^. The raw STORM data were analyzed according to previously described methods^53,54^.

Single-color 3D-STORM was performed using only Alexa Fluor 647 (Brp), while two-color 3D-STORM imaging was performed with Alexa Fluor 647 (Cac or Cpx) and CF680 (Brp in two-color mode). For two-color imaging with 647 nm excitation, a ratiometric detection scheme^55,56,92^ was employed to concurrently collect the emission of Alexa Fluor 647 and CF680 single molecules. Emission of these dyes was split into two light paths using a long pass dichroic mirror (T685lpxr; Chroma Technology Bellow Falls, VT), each of which was projected onto one half of the EM-CCD camera. Dye assignment was performed by comparing the intensity of each single molecule in the two channels. Proper localization assignments in this tissue were validated using *en face* AZs with anti-Cac/Alexa Fluor 647 and anti-Brp/CF680 localizations, ensuring robust label separation. STORM images were generated similarly to the QuaSOR images described above. Each STORM localization was rendered using a normalized 2D Gaussian intensity profile, with each image being a sum of filtered localizations providing density approximations. All STORM images are *z* projections unless otherwise noted, with similar localization Gaussian *σ* values (9–15 nm) with colors indicating approximate localization densities per pixel.

To quantify the degree of uniformity of excitation in 3D STORM over the field of view (FOV), we plotted total Brp and Cac localizations per AZ against radial distance from the center of the FOV. As shown in Supplementary Fig. 21, both Brp and Cac localization numbers varied widely and uniformly (~5% trend in opposite directions for Brp and Cac) over the range of radial distances from the center of the FOV, indicating uniform excitation.

For AZ-specific STORM quantifications, we first identified all AZ locations at a relatively low resolution (32 nm/px). Each AZ ROI was then rendered at a substantially higher resolution (1.6 nm/px), whereby individual AZ areas could be clearly identified and isolated, typically using a *z* projection of Brp localizations (Suppl. Video 1). These areas were used to identify isolated ROIs corresponding to individual AZs, with the ROI being used to calculate all AZ statistics. To account for variations in the STORM imaging conditions for each NMJ, we normalized the values within each NMJ to the largest AZ, unless otherwise noted. The orientation of each AZ was classified according to the relative Brp-defined AZ shape, AZ position within the bouton, the conventional Hrp image (which provided the relative location of membrane) and the relative locations of Brp and Cac in those datasets. Volumetric masking was accomplished by rendering STORM localizations in 3D at 20 nm increments in the *z* dimension. These 3D STORM images were used to generate a volume mask that collected STORM localizations within ~80 nm of Brp localization densities above a threshold density of ~1 localization per nm^2^ for each 20 nm *z* slice throughout the AZ.

Following the determination of the alignment parameters, each *en face* AZ image was translated to match a common center location. Localization data were then added and the subsequent pixel values were divided by the number of AZs included in the alignment to calculate the average localization density (Suppl. Video 2). Because construction of all STORM images used either unfiltered localization coordinates or normalized 2D Gaussians to represent each localization, the resulting images provide a measure of average localization density per pixel. For *en face* AZ, localization density radial profile calculations, localizations were binned into 10 nm annuli surrounding the center alignment position, with total counts being normalized to the area of each annulus.

### Structure-function matching

QuaSOR data were matched to the corresponding Airyscan or STORM data through a multi-stage approach (Suppl. Video 4). First, the two datasets were roughly aligned by rotating either the QuaSOR or Airyscan data to match the orientation of the corresponding map. In the case of the STORM imaging, before acquiring STORM data, we obtained conventional images of postsynaptic SynapGCaMP6f labeled with anti-GFP antibody and motor neuron axon membrane labeled with anti-Hrp antibody (Suppl. Fig. 6a). These were used to align the SynapGCaMP6f baseline fluorescence images obtained during quantal imaging in the living preparation (Suppl. Fig. 6b). Once roughly aligned, sites were matched to one another in a pairwise fashion taking care to match each STORM or Airyscan AZ to a corresponding location on the QuaSOR map. We used low-density areas to match all unambiguous AZ pairs with clear relative orientations and positions. Following these areas, we used the relative positioning of AZs around these pairs to match the denser regions or match AZs with no corresponding QuaSOR activity to an empty region of the QuaSOR map. In the case of the STORM matching, Brp STORM-defined AZ areas were matched to clusters of QuaSOR event localizations for 100–200 synapses per NMJ. In the Airyscan-matched data, we sorted all QuaSOR event coordinates to their nearest QuaSOR AZ centroid location. Both methods produced very similar results. To further ensure unbiased matching, a minimum of two people validated the site pairings prior to quantification. All pairs had to minimize the local alignment variance within each bouton as determined by the alignment vector between sites.

The quality of the AZ matching was further confirmed by using the paired coordinate positions to generate vectors to transform the QuaSOR coordinates into the matching STORM or Airyscan pixel-space. This was done by first converting the relative QuaSOR coordinates into a matched pixel-space image and then using a 2D, locally weighted, mean transformation method^93^, with groupings of 8–14 AZs being used as control points in the local weighting. Successful site pairing generated accurate remapping of QuaSOR events onto the appropriate AZ for either structural imaging technique. In all figures, these transformed images are denoted by “*Evoked” or “*Spontaneous”. However, all quantifications were performed on un-translated data in order to eliminate artifacts of transformations.

Additionally, we used an automated alignment, clustering, and matching process to confirm general trends within the data. This involved aligning the STORM and QuaSOR coordinate sets with an Affine registration and then taking advantage of a density-based spatial clustering of applications with noise (DBSCAN) to identify AZ clusters within both STORM and QuaSOR datasets. Corresponding AZ clusters were then matched, with interpolation to account for QuaSOR sites with no detected evoked responses. While this generated similar results, we found better performance with manual corrections of ROIs, as it allowed for proper separation of neighboring AZs in dense regions.

### Electrophysiology

Female third instar larvae were dissected as described above and all recordings were performed on muscle 4 from segment A3 only. Recording electrodes contained 3 M KCl and had resistances in the range 15–25 MΩ. For two-electrode voltage-clamp experiments, the membrane was held at −70 mV. All data were recorded at 5 kHz, with an AxoClamp-2A amplifier and Digidata 1322 A interface and Clampex 8.0 software (Molecular Devices, Sunnyvale, CA). For simultaneous electrophysiology measurements with imaging, the image acquisition was triggered by the Digidata so that the imaging was time-locked to the start of the electrophysiology recording. All electrophysiological recordings were analyzed with custom-written MATLAB routines. Briefly, all data were moving average low-pass filtered with a span of 2 ms. EPSPs and EPSCs were calculated as the difference between the peak response to the average baseline immediately prior to stimulation. At least 20 evoked responses were acquired at 0.1 Hz and then averaged to calculate the mean response with both recording types. For simultaneous mEPSP/mEPSC and optical recordings verified optical events and mEPSPs/mEPSCs were paired manually according to their relative timing and amplitudes. Evoked responses were also used to more accurately align the two data types.

### Statistics and reproducibility

Wilcoxon two-tailed signed-rank test was used to compare paired Ib-Is quantifications. Two-sample two-sided Kolmogorov–Smirnov tests or two-sample two-sided Cramér–von Mises tests were used to compare pooled cumulative distributions. One-way ANOVAs with Tukey-Kramer post hoc tests were used to compare mean NMJ Ib-Is properties for different genotypes, pooled data cumulative frequency distributions for different genotypes and distributions of data between multiple bins. One-dimensional Pearson’s correlation coefficients (*R*) were used to compare the radial density profiles for different QuaSOR event types (i.e. spontaneous versus evoked). 2D fitting of the binned data utilized linear regressions or non-linear regressions, while three-dimensional fits were calculated using a linear polynomial surface. All *R*^2^ values and fit equations are provided in the figure legends. Unless otherwise noted, reported values are mean ± SEM. The specific statistical tests as well as the number of replicates including numbers of animals, activity bouts, NMJs or AZs are all provided in the corresponding figures and/or figure legends. For all figures, significance markers are *p < 0.05, **p < 0.01, ***p < 0.001, ****p < 0.0001 or NS not significant for the comparisons indicated in the figure or figure legend.

Representative QuaSOR, STORM, and Airyscan images presented in figures were all reproduced in multiple animals. To assess reproducibility, recordings were done on only one NMJ per larva (i.e. the number of NMJs = the number of larvae) in recordings that were performed within 30 min of the beginning of the dissection, thereby ensuring health and similar conditions. We studied muscle 4 in three segments: A3-A5. To determine whether we could pool the results from these three segments, we analyzed recordings from WT animals from A3 (*n* = 9), A4 (*n* = 11), and A5 (*n* = 13) and compared their properties. We found no significant difference between Ib synapses in the three segments in the following key parameters: (a) Quantal density, (b) Average *P*_*r*_, (c) Brp STORM localizations per AZ, and (d) normalized Brp per AZ. This analysis indicates that muscle 4 behaves the same way in A3, A4, and A5. Based on this, we pooled results from these segments for the rest of the analysis. The number of NMJs for each experiment ranged from 7 to 33, as shown in Suppl. Table 1.

### Reporting summary

Further information on research design is available in the Nature Research Reporting Summary linked to this article.

## Supplementary information

Supplementary Information

Description of Additional Supplementary Files

Supplementary Video 1

Supplementary Video 2

Supplementary Video 3

Supplementary Video 4

Reporting Summary

## Data Availability

Source data are provided as a Source Data file and have been deposited in the Figshare repository under the filename 10.6084/m9.figshare.17041718.v1. Source data are provided with this paper.

## Source data

Source Data

## Code availability

The custom code written for this study will be available in the GitHub repository under the filename https://github.com/newmanza/Newman_QuaSOR_2021; 10.5281/zenodo.5711302.
