# Supplementary material for: Determinants of synapse diversity revealed by super-resolution quantal transmission and active zone imaging
Source: Nat Commun. 2022 Jan 11;13:229. doi: 10.1038/s41467-021-27815-2 (PMC8752601; doi:10.1038/s41467-021-27815-2)
Supplement: Supplementary file 1 — Supplementary Information [file 41467_2021_27815_MOESM1_ESM.pdf]

Supplementary Information

Determinants of synapse diversity revealed by super-resolution quantal transmission and active zone imaging

Zachary L. Newman[1], Dariya Bakshinskaya[2], Ryan Schultz[2], Samuel J. Kenny[4], Seonah Moon[4], Krisha Aghi[2], Cherise Stanley[1], Nadia Marnani[1], Rachel Li[1], Julia Bleier[2], Ke Xu[2,4,5] and Ehud Y. Isacoff[1,2,3,5,*]

Supplementary Figure Legends

Supplementary Figure 1: QuaSOR fitting of evoked and spontaneous transmission events; related to Figure 1.

a-f, Rapid spatial spread of quantal SynapGCaMP6f responses. Two example spontaneous events (imaged at 50 FPS) showing mean SynapGCaMP6f ΔF/F traces (**a,d**), sequential ΔF/F frames (**b,e**), and the isolated response following event detection (**c,f**). **g-h**, Same event in **Fig. 1a-d** with the isolated ΔF/F response and contours lines showing the QuaSOR 2D Gaussian fit model in **g** and a 3D representation of the fit model in **h**. **i-j**, Same AP-evoked events in **Fig. 1e-h** with the isolated ΔF/F response and contours lines showing the corresponding merged QuaSOR 2D Gaussian mixture model in **i** and a 3D representation of the mixture model in **j**. **k-n**, Aligned localizations (**k,m**) and 2D Gaussian fits (**l, n**) to evoked transmission events from same data set (10,363 AZs from 44 NMJs) using either the pixel maxima method (**k,l**) or QuaSOR (**m,n**). Images above, spatial profiles below (in all panels data with error bands are presented as mean ± SD). **o-p**, Evoked event cluster full width at half max (FWHM) (**o**) and evoked event cluster half-maximal area (**p**) for the 44 NMJs showing significantly better resolution for QuaSOR (**o** Wilcoxon two-tailed signed-rank *p* = p = 7.59×10^{-9} ;**p** Wilcoxon two-tailed signed-rank *p* = p = 7.62×10^{-9}). Scale bars: 1 µm (**b,e,g,i**) and 200 nm (**k-n**).

Supplementary Figure 2: QuaSOR analysis of evoked and spontaneous transmission; related to Figure 1.

a-c, Evoked and spontaneous events do not colocalize in WT NMJs. **a,** Evoked (cyan) and spontaneous (red) QuaSOR transmission event locations (σ = 42.3 nm) in Ib and Is NMJs onto a common muscle 4, with one terminal Ib bouton ROI indicated (white square). **Inset,** Baseline SynapGCaMP6f fluorescence. **b**, Terminal bouton from (**a**) magnified. **c**, QuaSOR transmission clusters from (**b**) further magnified. **d**, QuaSOR-defined active site density, including evoked and spontaneous QuaSOR event clusters (n = 48 NMJ pairs; Wilcoxon two-tailed signed-rank test p = 0.018). **e**, Mean P_r. of QuaSOR-defined transmission sites (Wilcoxon two-tailed signed-rank test $p = 1.6*10^{-9}$). Scale bars: 10 µm (**a**), 2 µm (**b**) and 200 nm (**c**).

Supplementary Figure 3: Cac labeling in control and CacKD animals; related to Figure 2.

a,b, Cac antibody staining of Ib bouton labels puncta that overlap with Brp puncta in a control NMJ (**a**) but is almost eliminated in the CacKD (**b**), indicating that the antibody is specific for Cac. Scale bars: 2 µm.

Supplementary Figure 4: Brp and Cac in Ib and Is axons; related to Figure 2.

a-b, Is AZs slightly smaller than Ib AZs. Mean 3D-STORM Brp AZ area (nm^2) for Ib-Is axon pairs innervating the same muscle 4 (**a**; n = 9 NMJ Ib-Is pairs; Wilcoxon two-tailed signed-rank test p = 0.031) and pooled cumulative frequency distributions (**b**; Ib n = 1707 AZs, n = 9 NMJs; Is n = 608 AZs, n = 9 NMJs; two-sample two-sided Kolmogorov–Smirnov test $p = 2.2*10^{-7}$). **c-d**, Similar Cac levels in Ib and Is AZs. Mean total Cac 3D-STORM AZ localizations for Ib-Is axon pairs (**c**; n = 9 NMJ Ib-Is pairs; Wilcoxon two-tailed signed-rank test p = 0.69) and pooled cumulative frequency distributions (**d**; Ib n = 1707 AZs, n = 9 NMJs; Is n = 608 AZs, n = 9 NMJs; two-sample two-sided Kolmogorov–Smirnov test p = 0.38)**. e-f**, Similar Brp levels in Ib and Is AZs. Mean total Brp 3D-STORM AZ localizations for Ib-Is axon pairs (**e**; n = 9 NMJ Ib-Is pairs; Wilcoxon two-tailed signed-rank test p = 0.16) and pooled cumulative frequency distributions (**f**; Ib n = 1707 AZs, n = 9 NMJs; Is n = 608 AZs, n = 9 NMJs; two-sample two-sided Kolmogorov–Smirnov test p = 0.033) **g-h**, Cac density is higher in Is than in Ib AZs. Mean Cac 3D-STORM AZ localization density for Ib-Is axon pairs (**g**; n = 9 NMJ Ib-Is pairs; Wilcoxon two-tailed signed-rank test p = 0.047) and pooled cumulative frequency distributions (**h**; Ib n = 1707 AZs, n = 9 NMJs; Is n = 608 AZs, n = 9 NMJs; two-sample two-sided Kolmogorov–Smirnov test p = 0.0017). **i-j**, Similar Brp densities in Ib and Is AZs. Mean Brp 3D-STORM AZ localization density for Ib-Is axon pairs (**i**; n = 9 NMJ Ib-Is pairs; Wilcoxon two-tailed signed-rank test p = 0.30) and pooled cumulative frequency distributions (**j**; Ib n = 881 AZs, n = 9 NMJs; Is n = 369 AZs, n = 9 NMJs; Kolmogorov–Smirnov test p = 0.89). All data Mean ± SEM.

Supplementary Figure 5: Brp and Cac STORM AZs in WT and *rab3rup* NMJs; related to Figure 2.
a-d, STORM images of Ib AZs from WT (**a**) and *rab3rup* (**b**) show that *rab3rup* AZs are larger and contain more Cac puncta, with more Brp localizations per AZ in both Ib and Is axons (**c,** One-way ANOVA $p = 1.8*10^{-7}$; Tukey-Kramer *post hoc* test: WT Ib vs *rab3rup* Ib $p = 5.5*10^{-7}$; WT Is vs *rab3rup* Is $p = 0.00073$; data are presented as mean ± SEM) and more Cac localizations per AZ in both Ib and Is axons (**d,** One-way ANOVA $p = 0.00015$; Tukey-Kramer *post hoc* test: WT Ib vs *rab3rup* Ib $p = 0.36$ WT Is vs *rab3rup* Is $p = 0.00068$; data are presented as mean ± SEM).

Supplementary Figure 6: QuaSOR matching to 3D-STORM AZ maps; related to Figure 3.
a, SynapGCaMP6f (green) and motor neuron membrane (Hrp, red) reference image overlay collected immediately prior to STORM imaging for the example found in **Fig. 3** with Ib (cyan border) and Is (orange border) NMJ regions indicated. **b**, Live baseline SynapGCaMP6f fluorescence image rotated to the corresponding orientation of the STORM data in **a**. **c**, Brp *z*-position depth-colored STORM image with **Insets** showing small ROIs also found in **Fig. 3a-d**. **d-f**, QuaSOR-STORM AZ matching and alignment in an example Ib NMJ. **d**, Brp (magenta) and Cac (green) 3D-STORM *z*-projection image (**d, left**) and QuaSOR Ib NMJ area image (σ = 42.3 nm), showing evoked (cyan) and spontaneous (red) transmission events (**d, right**) with yellow lines connecting AZs to their corresponding transmission sites. **e,f**, A map of relative positions of AZs from STORM (**e**) shows a similar pattern to a map of relative positions of transmission sites from QuaSOR (**f**), **g**, Four example boutons from different NMJs at high magnification show close alignment of AZs imaged in STORM (**left**; Brp and Cac) and QuaSOR defined sites of evoked transmission (**right**; *evoked; σ = 42.3 nm). **h,i**, An average of ~85% of QuaSOR transmission sites could be matched to a STORM imaged AZ (**h**) with an average offset correction of 257.0 nm ±- 9.5 nm (**i**) (data are presented as mean ± SEM). Scale bars: 5 µm (**a-f**) and 1 µm (**g**).

Supplementary Figure 7: Diversity in evoked and spontaneous activity patterns across AZs; related to Figure 3.
a, Heterogeneous evoked transmission patterns. Raster for a single Ib NMJ example in **Fig. 3a-d** and **Supplementary Fig. 4** showing all AZs and all 200 stimulus trials. **b**, Low percentage AP participation of Ib AZs. Mean percent of AZs participating in each evoked stimulus trial (n =16 Ib NMJs; data with error bands are presented as mean ± SEM). **c**, Extremely low spontaneous rates throughout the Ib NMJ. Spontaneous event raster for all Ib AZs representing four 30 s movies. **d,** Minimal spontaneous release of Ib AZs. Mean percent of AZs active per second for 120 s total of spontaneous event imaging (n = 16 Ib NMJs; data with error bands are presented as mean ± SEM).

Supplementary Figure 8: QuaSOR matching to 3D-Airyscan AZ maps; related to Figure 3.

a, Maximum intensity projection 3D-Airyscan tiled dataset for Brp (magenta), SynapGCaMP6f (green), and Hrp (gray). Areas outside the tiled region are indicated by the gray background with Ib (cyan border) and Is (orange border) axons indicated. **b**, Live baseline SynapGCaMP6f fluorescence image aligned to the Airyscan data in **a**. **c-f**, Maximum intensity projection 3D-Airyscan tiled dataset for the Ib axon showing only Brp (gray) (**c**) along with single *z*-slice images of the indicated three example *en face* oriented AZs along with a line intensity profile through each (**d-f**). Note that AZ dimensions are similar to those found with STORM in **Fig. 2**. **g**, Evoked (cyan) and spontaneous (red) Airyscan-aligned QuaSOR overlay image (σ = 63.5 nm) for the Ib NMJ in **c**. **h-l,** Local QuaSOR-Airyscan alignment showing the white rectangular ROI from **a,c,g**. Images include the ROI with Brp/SynapGCaMP6f overlay (**h**), the Brp/Hrp overlay (**i**), Brp only (**j**), the Airyscan-aligned spontaneous and evoked QuaSOR coordinates (Evoked, cyan; Spont, red; σ = 42.3 nm) (**k**) and the aligned QuaSOR overlaid on Brp (**l**). Scale bars: 10 µm (**a-c,g**), 400nm (**d-f**) and 1 µm (**h-l**).

Supplementary Figure 9: Relationship between P_r and F_s and their dependence on Brp and Cac; related to Figure 4.

a, P_r dependence on core AZ Cac density. P_r-binned mean core Cac radial localization density (40 nm radius) versus mean binned AP-evoked P_r for *en face*-aligned AZ profile data plotted in **Fig. 3k** (R^2 = 0.999; $y=8*10^6x^{4.7}$ +0.0007). **b,** P_r-binned, mean F_s *versus* mean P_r from pooled Brp/Cac 3D-STORM matched to QuaSOR dataset for WT Ib AZs (all AZ orientations; 200 stim. 2 min spontaneous; R^2=0.958; y=0.043x+0.004; n = 1409 AZs from 9 NMJs). **Inset** shows a zoomed view of the relationship. **c**, P_r-binned, mean F_s *versus* mean Cac normalized total localizations per AZ for WT Ib AZs (QuaSOR-STORM dataset; R^2=0.929; y=0.036x-0.002; n = 1409 AZs from 9 NMJs). **d**, P_r-binned, mean F_s *versus* mean Brp normalized total localizations per AZ for WT Ib AZs (QuaSOR-STORM dataset; R^2=0.963; y=0.026x-0.0002; n = 1409 AZs from 9 NMJs).

Supplementary Figure 10: Synaptic transmission in *rab3^rup* mutant; related to Figure 4.

a,b, Aligned STORM and QuaSOR images from an example *rab3^rup* Ib bouton. **a**, STORM image showing localizations for Brp (magenta) and Cac (green). **b**, QuaSOR image showing localizations of spontaneous (red) and evoked (cyan) transmission events. **c**, Ib P_r distribution for *rab3^rup* (red) is shifted to higher P_r values compared to WT (black, WT data is from **Fig. 3e**; two-sample two-sided Kolmogorov–Smirnov test $p = 9.1*10^{-57}$). **d**, P_r – Cac STORM localization relation for *rab3^rup* is similar to that of WT but extends to higher values along both axes (data are presented as mean ± SEM). **e,** F_s - P_r relation is shallower in *rab3^rup* than in WT (data are presented as mean ± SEM).

Supplementary Figure 11: Cpx detection in Ib and Is boutons; related to Figure 5.

a-f, Detection of Cpx in Airyscan. Example Airyscan maximum intensity *z*-projection images for boutons in Ib (**a-c**) and Is (**d-e**) axons of Control (**a,d**), CpxKD (**b,e**) and CpxOE (**c,f**). showing Brp (magenta;) and Cpx (green). Images acquired and processed with identical settings. **g-i,** STORM imaging of Ib terminal boutons from same Control (**g**), CpxKD (**h**) and CpxOE (**i**) genotypes as (**a-f**), with same antibody stains and color scheme, and adding merged images of Brp (magenta) and Hrp (white) on right. Genotypes were: Control (OK6-Gal4, attP40^{Empty}, SynapGCaMP6f), CpxKD (OK6-Gal4, UAS-Cpx^{RNAi}, SynapGCaMP6f) and CpxOE (OK6-Gal4, UAS-Cpx, SynapGCaMP6f. Scale bars: 2 (**a-f**), 1 µm (**g-i**).

Supplementary Figure 12: Cpx expression in Ib and Is boutons; related to Figure 5.

a,b, Expression levels of Cpx in Ib and Is axons. **a**, Mean background subtracted terminal axon Cpx fluorescence intensity for Control (n = 5 Ib and n = 5 Is NMJs), CpxKD (n = 5 Ib and n = 5 Is NMJs), and CpxOE (n = 5 Ib and n = 3 Is NMJs) animals (data are presented as mean ± SEM; One-way ANOVA $p = 7.9*10^{-10}$ with Tukey-Kramer *post hoc* test Control Ib vs. Is $p = 0.0028$; CpxKD Ib vs. Is $p = 0.90$; CpxOE Ib vs. Is $p = 0.081$; Control Ib vs. CpxKD Ib $p = 0.0015$; Control Ib vs. CpxOE Ib $p = 0.0012$; Control Is vs. CpxKD Is $p = 0.78$; Control Is vs. CpxOE Is $p = 0.00012$). **b**, Type Ib synapses have higher levels of Cpx per AZ than does Is. Mean total Cpx 3D-STORM AZ localization pooled cumulative frequency distributions for WT Ib and Is axons (Ib n = 864 AZs from n = 7 NMJs; Is n = 206 AZs from n = 7 NMJs; two-sample two-sided Kolmogorov–Smirnov test $p = 2.4*10^{-16}$).

Supplementary Figure 13: Relationship between Cpx and Brp associated with differences in P_r; related to Figure 5.

a, Weak relationship between Cpx and F_s. P_r-binned, 3D-filtered (note, only localizations within ~80 nm of the Brp localization volume are counted), mean Cpx NMJ-normalized total localizations versus mean F_s for WT Ib AZs (QuaSOR-STORM dataset; all AZ orientations; 200 stim. 2 min spontaneous; R^2=0.884; y=0.025x-0.0038; data are presented as mean ± SEM). **Inset** shows a zoomed view of the relationship. **b**, Relationship between Brp and Cpx. P_r-binned, 3D-filtered, Brp versus Cpx mean NMJ-normalized total localizations for WT Ib AZs (R^2=0.960; y=0.84x+0.0045; data are presented as mean ± SEM). **c**, P_r dependence on Cpx. P_r-binned, 3D-filtered, mean Cpx NMJ-normalized total localizations per AZ versus mean P_r for WT Ib AZs (R^2=0.997; $y=85x^7+0.0004$; data are presented as mean ± SEM). **d**, Relationship between P_r, core Cpx density and core Brp density. P_r-binned, mean evoked P_r versus mean integrated core radial localization densities (40 nm radius) for Brp (magenta) or Cpx (green) in all *en face*-aligned AZs (data are presented as mean ± SEM).

Supplementary Figure 14: Spontaneous and evoked transmission in the CpxKD; related to Figure 6.
a-f, Comparison of synaptic transmission in Control (OK6-Gal4, attp40^{empty}, SynapGCaMP6f) to CpxKD (OK6-Gal4, UAS-Cpx^{RNAi}, SynapGCaMP6f). Evoked EPSP and spontaneous mEPSPs in Control **(a)** and CpxKD **(b)** measured in single electrode bridge recording. Spontaneous mEPSCs in Control (**c**) and CpxKD (**d**) and evoked EPSCs in Control **(e)** and CpxKD **(f)** measured in two-electrode voltage clamp (low-pass filtered). **g-i**, Muscle properties (resting potential, **g**; input resistance, **h**; leak current, **i**) are unchanged in CpxKD (data are presented as mean ± SEM). **g-i**, EPSP slightly decreased (but not statistically significant) (**j**) and EPSC significantly decreased (**k**) in CpxKD (data are presented as mean ± SEM). Smaller change in EPSP may be due to non-linear summation.

Supplementary Figure 15: CpxKD decreases optical spontaneous transmission event amplitude; related to Figure 6.
a,b, Optical spontaneous transmission event amplitude (ΔF/F) decreased at both Ib (**a**) and Is (**b**) synapses by CpxKD.

Supplementary Figure 16: CpxKD increases spontaneous transmission frequency; related to Figure 6.
a, Example ΔF/F traces from ten 30 s movies for a single AZ from a WT Ib QuaSOR-STORM matched NMJ. Synaptic transmission events elicited at that AZ are indicated by dots marking event peaks (cyan = evoked; red = spontaneous; no dot = event elicited elsewhere). Stimulus timing indicated below traces (yellow). Continuous imaging bouts with 5 stimuli per bout at 0.2 Hz. **b-c**, Mean ΔF/F traces for all AZs and all movies for a single WT Ib (**b**; n = 136 AZs and 20 movies) or Is (**c**; n = 44 AZs and 20 movies) NMJ (data with error bands are presented as mean ± SEM). **d**, Example ΔF/F traces from ten 30 s movies for a single AZ from a CpxKD (OK6-Gal4, UAS-Cpx^{RNAi}, SynapGCaMP6f) Ib QuaSOR-STORM matched NMJ. **e-f**, Mean ΔF/F traces for all AZs and all movies for a single CpxKD Ib (**e**; n = 617 AZs and 20 movies) or Is (**f;** n = 277 AZs and 20 movies) NMJ (data with error bands are presented as mean ± SEM).

Supplementary Figure 17: CpxKD synapses retain diversity in evoked and spontaneous transmission throughout the NMJ; related to Figure 6.
a, Global evoked release heterogeneity in CpxKD Ib NMJs. CpxKD event raster for all evoked events at each AZ within the example NMJ in **Fig. 6**. Gray rows highlight AZs 38 and 176 from **Fig. 6**. **b**, CpxKD mean percent of active AZs for each evoked stimulus trial (100 stimuli; n = 6 NMJs; data with error bands are presented as mean ± SEM). **c**, High and diverse spontaneous release rates in CpxKD Ib NMJs. Raster

for all detected spontaneous events (20, 30 s movies) at every type Ib AZ within the example NMJ. Gray rows highlight AZs 38 and 176 from **Fig. 6**. **d**, High spontaneous participation in CpxKD Ib NMJs. CpxKD mean percent of spontaneously active AZs per second (600 s; n = 6 NMJs; data with error bands are presented as mean ± SEM).

Supplementary Figure 18: CpxKD increases F_s without altering evoked transmission or AZ density; related to Figure 7.

a, CpxKD increases F_s. Mean F_s per NMJ for QuaSOR-Airyscan matched Control Ib (n = 2709 AZs, 7 NMJs; OK6-Gal4, attP40^{Empty}, SynapGCaMP6f), Control Is (n = 1114 AZs, 7 NMJs), CpxKD Ib (n = 1547 AZs, 5 NMJs; OK6-Gal4, UAS-Cpx^{RNAi}, SynapGCaMP6f), and CpxKD Is (n = 697 AZs, 5 NMJs) animals (One-way ANOVA $p = 3.2*10^{-11}$; Tukey-Kramer *post hoc* test: Control Ib vs. Control Is $p = 0.99$; CpxKD Ib vs. CpxKD Is $p = 3.0*10^{-6}$; Control Ib vs. CpxKD Ib $p = 0.00012$; Control Is vs. CpxKD Is $p = 3.9*10^{-9}$). **b**, CpxKD NMJs lack sites with no spontaneous transmission. Percent of AZs with no spontaneous transmission (QuaSOR-Airyscan matched; One-way ANOVA $p = 2.2*10^{-6}$; Tukey-Kramer *post hoc* tests: Control Ib vs. Control Is $p = 0.0064$; CpxKD Ib vs. CpxKD Is $p = 0.999$; Control Ib vs. CpxKD Ib $p = 0.018$; Control Is vs. CpxKD Is $p = 1.1*10^{-5}$). **c**, CpxKD does not increase AP-evoked quantal density. Optical quantal density per NMJ (quantal content per µm^2 SynapGCaMP6f area; QuaSOR-Airyscan matched; One-way ANOVA $p = 0.0086$; Tukey-Kramer *post hoc* tests: Control Ib vs. Control Is $p = 0.094$; CpxKD Ib vs. CpxKD Is $p = 0.031$; Control Ib vs. CpxKD Ib $p = 0.97$; Control Is vs. CpxKD Is $p = 0.94$). **d**, CpxKD does not increase AP-evoked P_r. Mean AP-evoked P_r per NMJ (QuaSOR-Airyscan matched; One-way ANOVA p = 2.2*10-6; Tukey-Kramer *post hoc* tests: Control Ib vs. Control Is $p = 0.00067$; CpxKD Ib vs. CpxKD Is $p = 0.0042$; Control Ib vs. CpxKD Ib $p = 0.999$; Control Is vs. CpxKD Is $p = 0.99$). **e**, CpxKD does not increase AZ density. AZ density per NMJ (AZs per µm^2 NMJ area; QuaSOR-Airyscan matched; One-way ANOVA $p = 0.0041$; Tukey-Kramer *post hoc* tests: Control Ib vs. Control Is $p = 0.017$; CpxKD Ib vs. CpxKD Is $p = 0.061$; Control Ib vs. CpxKD Ib $p = 1$; Control Is vs. CpxKD Is $p = 0.999$). **f-g**, CpxKD Ib AZs similar in size to WT Ib. Pooled *en face*-aligned AZ radial density profiles (**f**) for WT Ib (black; n = 129 AZs, 2 NMJs) and CpxKD (blue; n = 655 AZs, 6 NMJs) and corresponding normalized radial density profiles (**g**). **h**, Reduced Brp in CpxKD Ib. Distributions for mean Airyscan Brp voxel intensities for Control Ib (n = 2333 AZs, 7 NMJs) and CpxKD Ib (n=1482 AZs, 5 NMJs) AZs (two-sample two-sided Kolmogorov–Smirnov test $p = 3.0*10^{-52}$). **i**, Additional evidence for reduced Brp in CpxKD STORM and Airyscan (two-sample two-sided Kolmogorov–Smirnov tests; mean ± SEM).

Supplementary Figure 19: Knockdown of Cpx dramatically steepens the relationship between evoked (P_r) and spontaneous (F_s) transmission and the dependence of P_r and F_s on Brp content; related to Figure 7.

a,b, F_s dependence on Brp is increased in the CpxKD (**b**) compared to WT (**a**). **c**, F_s highly related to P_r in CpxKD NMJs. QuaSOR-Airyscan matched, P_r-binned, mean P_r *versus* mean F_s data for pooled Control Ib AZs (black; OK6-Gal4, attP40^{Empty}, SynapGCaMP6f; n=2709 AZs from 7 NMJs; y=0.048x+0.0044; R^2 = 0.965) and CpxKD Ib AZs (blue; OK6-Gal4, UAS-Cpx^{RNAi}, SynapGCaMP6f; n=1547 AZs 5 NMJs; y=0.96x+0.026; R^2 = 0.989) (data are presented as mean ± SEM). **D**, F_s dependence on Brp is greatly increased in the CpxKD. QuaSOR-Airyscan matched, P_r-binned, mean Brp voxel intensity *versus* mean F_s for pooled Control Ib AZs (black; y=7.63*10^{-6}x-0.0081; R^2 = 0.963) and CpxKD Ib AZs (blue; y=2.51*10^{-4}x-0.027; R^2 = 0.821) (data are presented as mean ± SEM).

Supplementary Figure 20: Minimal differences between Ib synapses onto muscle 4 in segments A3-A5, related to Figures 1-7.

Ib synapses onto muscle 4 are similar in quantal density (**a**; One-way ANOVA p = 0.10 with Tukey-Kramer *post hoc* test A3 vs A4 p = 0.50; A4 vs A5 p = 0.41; A3 vs A5 p = 0.084), average P_r (**b**; One-way ANOVA p = 0.028 with Tukey-Kramer *post hoc* test A3 vs A4 p = 0.96; A4 vs A5 p = 0.10; A3 vs A5 p = 0.042), Brp STORM localizations per AZ (**c**; One-way ANOVA p = 0.45 with Tukey-Kramer *post hoc* test A3 vs A4 p = 0.99; A4 vs A5 p = 0.62; A3 vs A5 p = 0.49) and average normalized Brp localizations per AZ (**d**; One-way ANOVA p = 0.32 with Tukey-Kramer *post hoc* test A3 vs A4 p = 0.41; A4 vs A5 p = 0.33; A3 vs A5 p = 0.99).

Supplementary Figure 21: Uniform STORM excitation across field of view, related to Figures 2, 3, 5 and 6.

(**a**) and Cac (**b**) localizations per AZ as a function of radial distance from the center of the FOV show uniformity (~5% trend in opposite directions for Brp and Cac) in TIRF excitation for STORM imaging over the range of radial distances (linear regressions indicated; **a**, y=32.7x+3220 R^2 = 0.012; **b**, Cac y = -2.87x+466 R^2 =0.0058).

Supplementary Table 1. Summary of experiments. Number of muscle-4 NMJs (one per larva) along with abdominal segment identity (A3-A5) for each experimental set.

Supplementary Figure 1

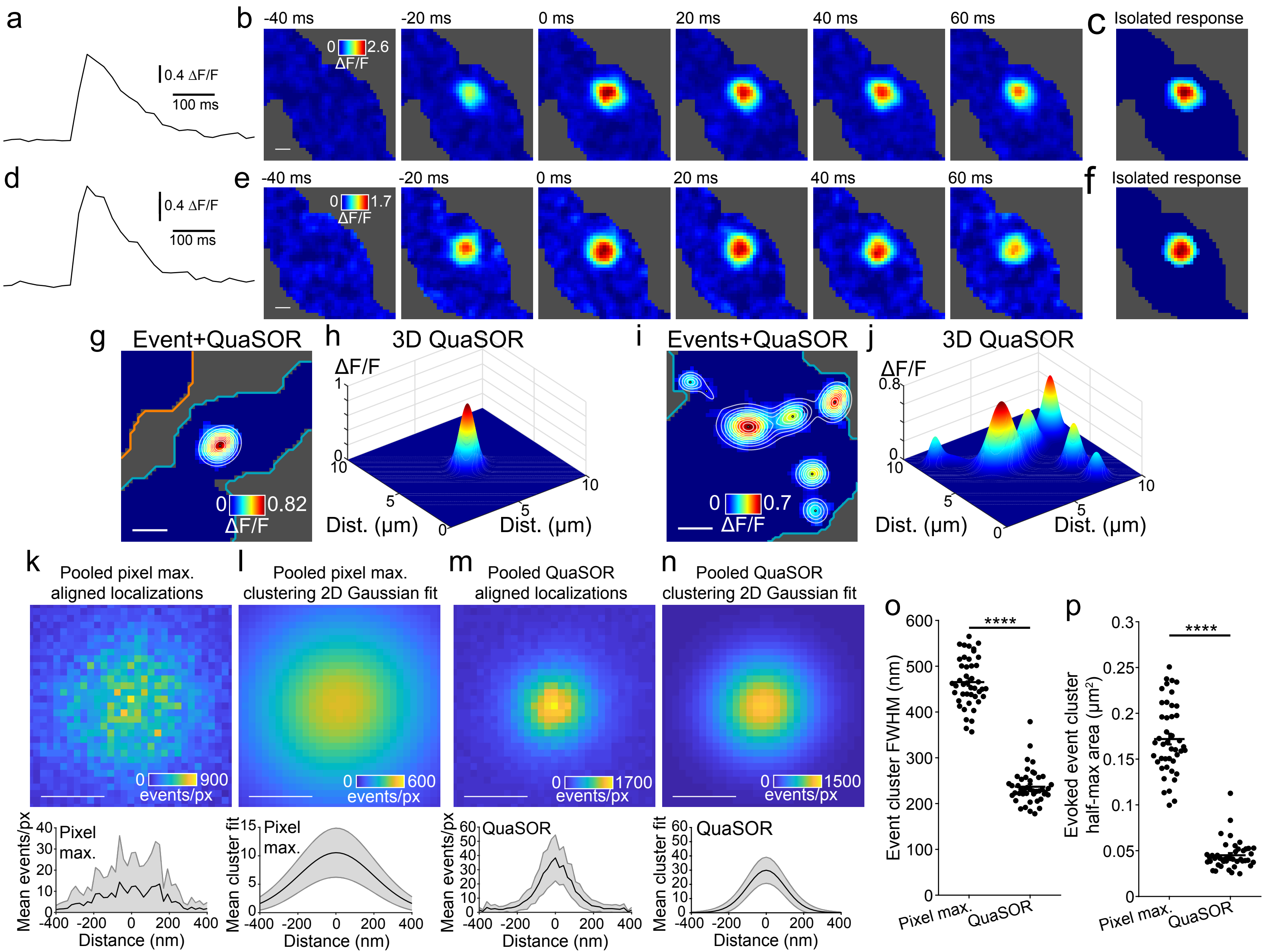

Supplementary Figure 2

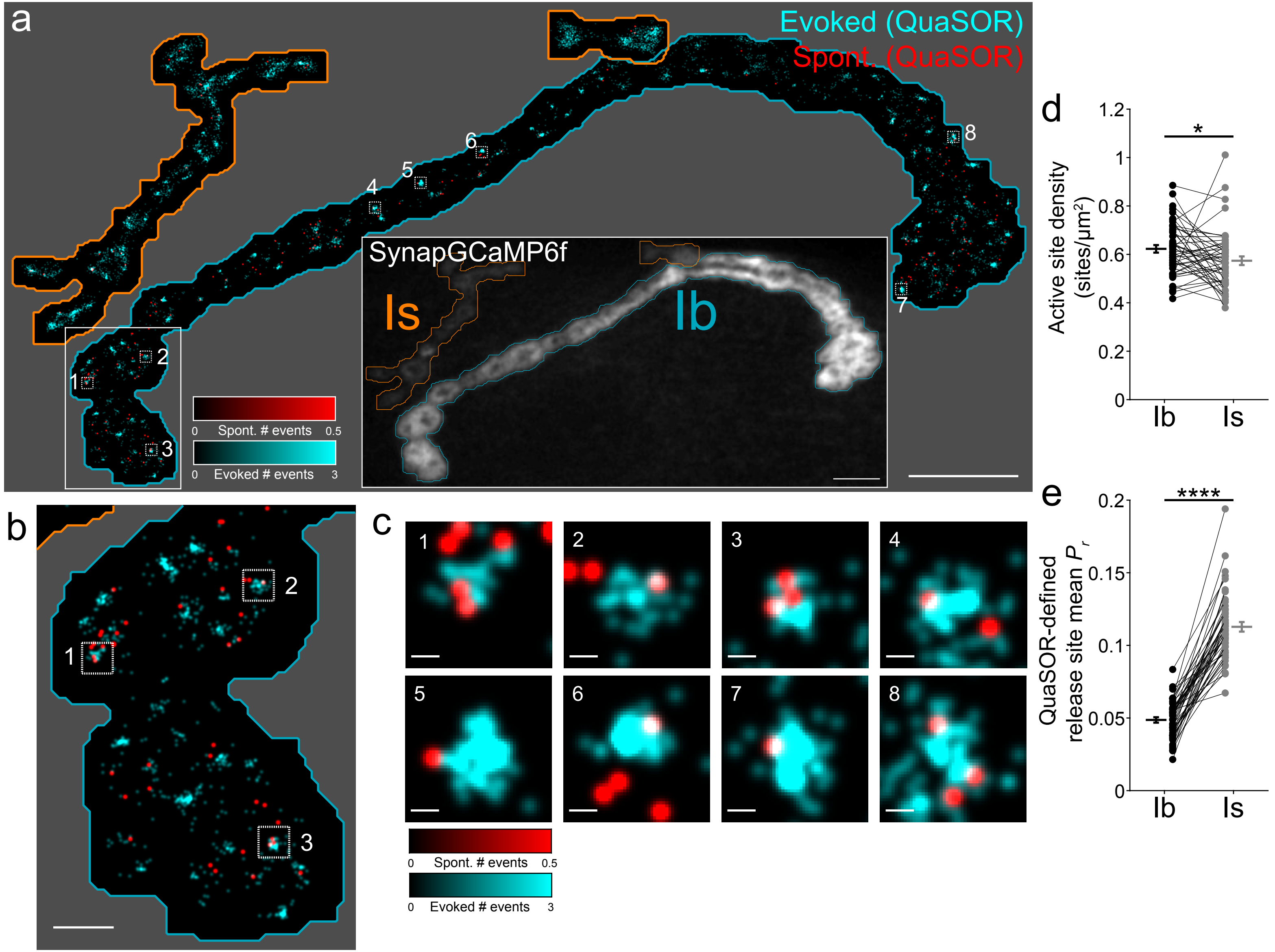

Supplementary Figure 3

a Control Ib NMJ

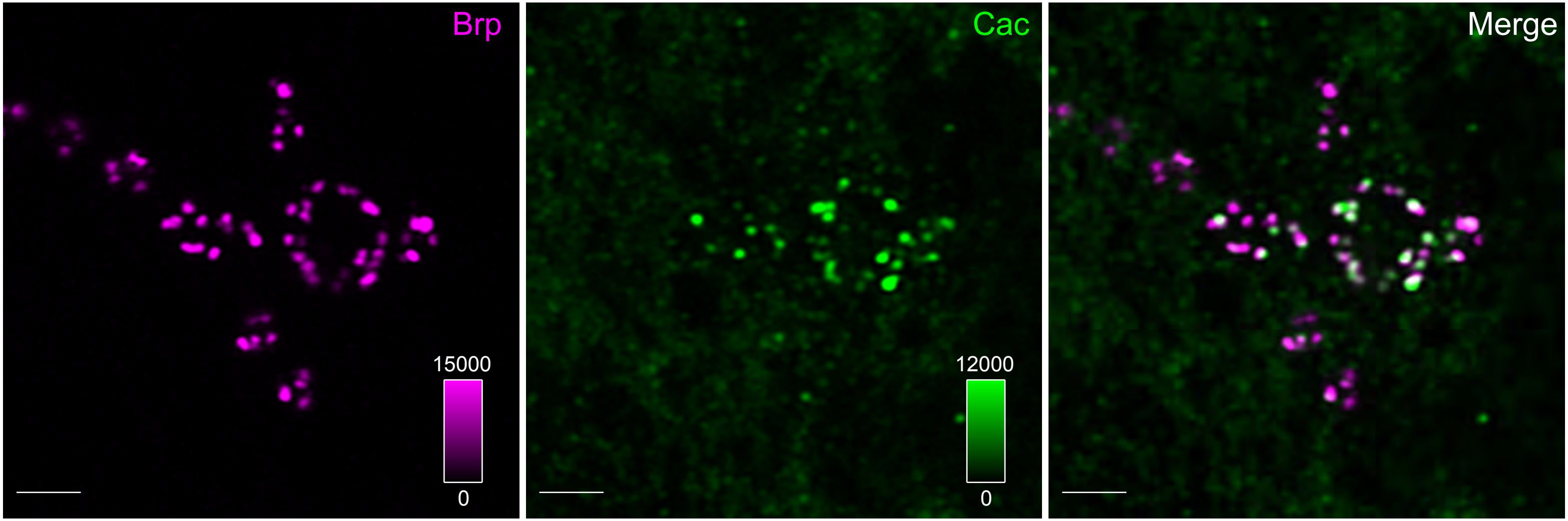

b CacKD Ib NMJ

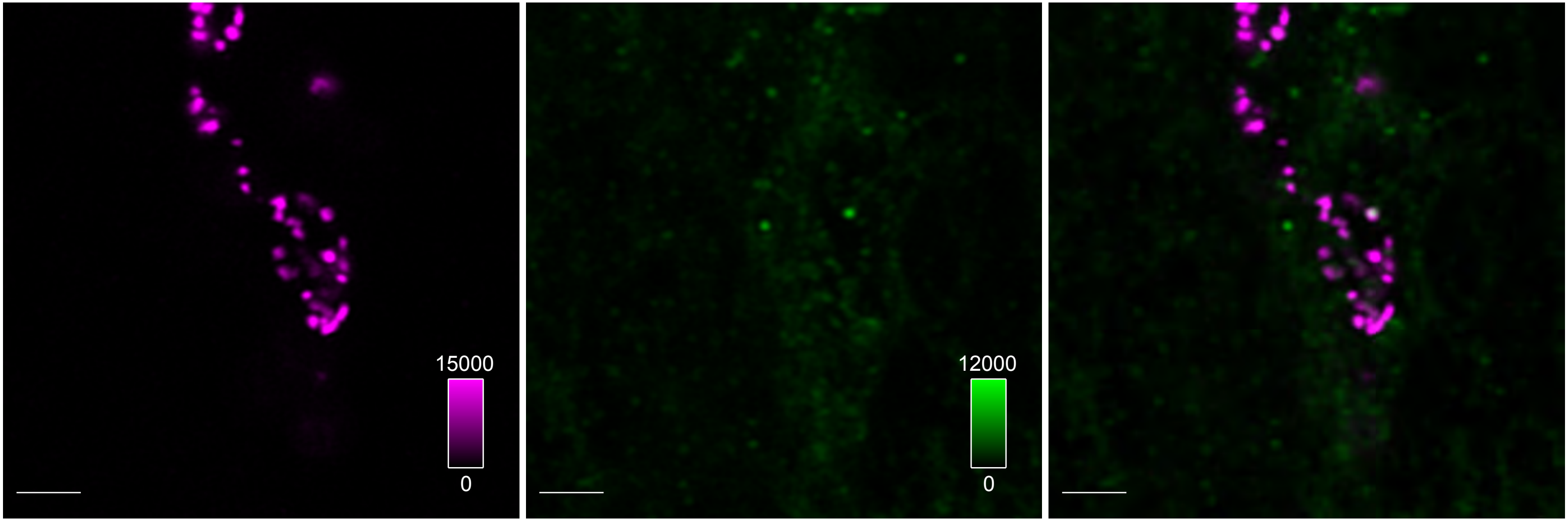

Supplementary Figure 4

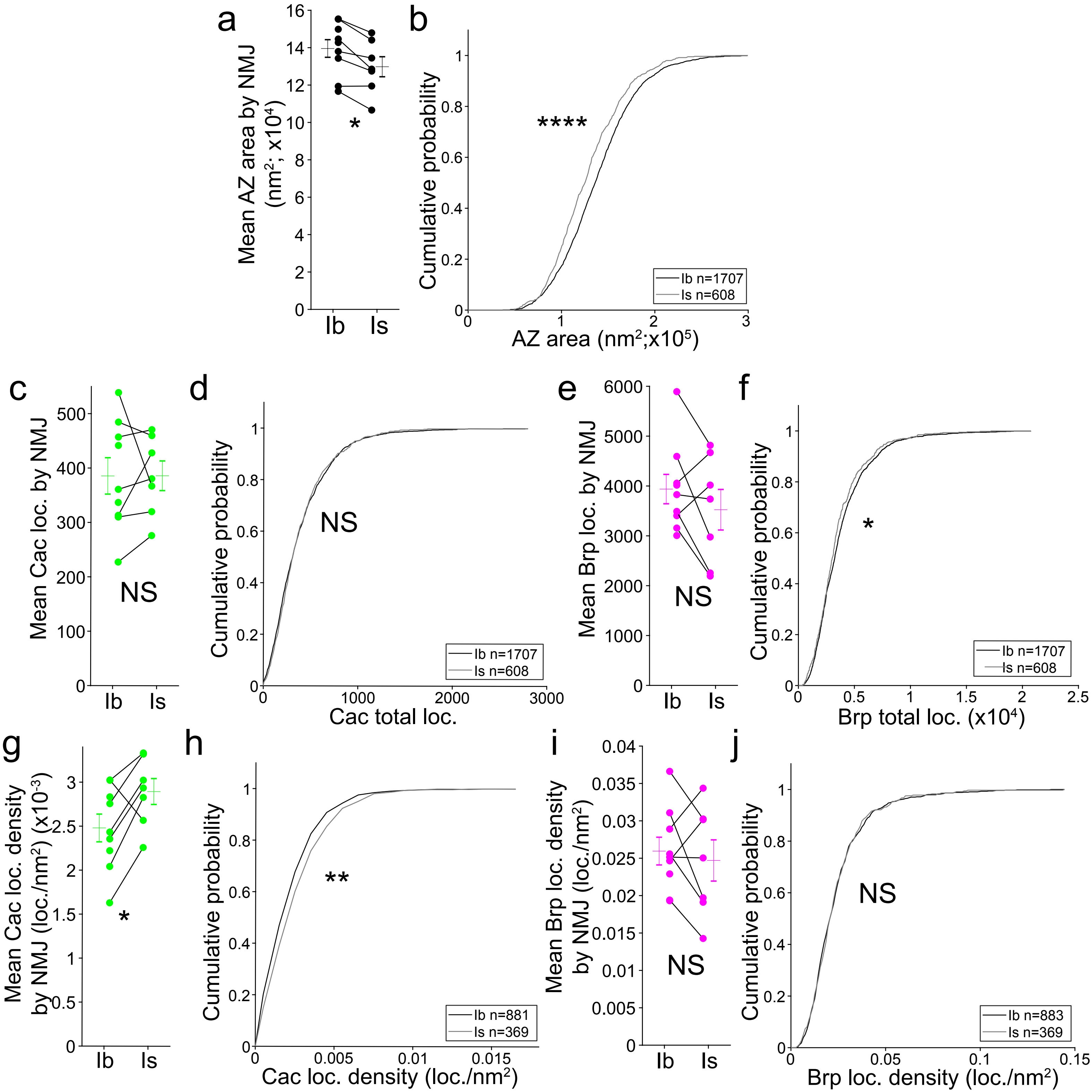

Supplementary Figure 5

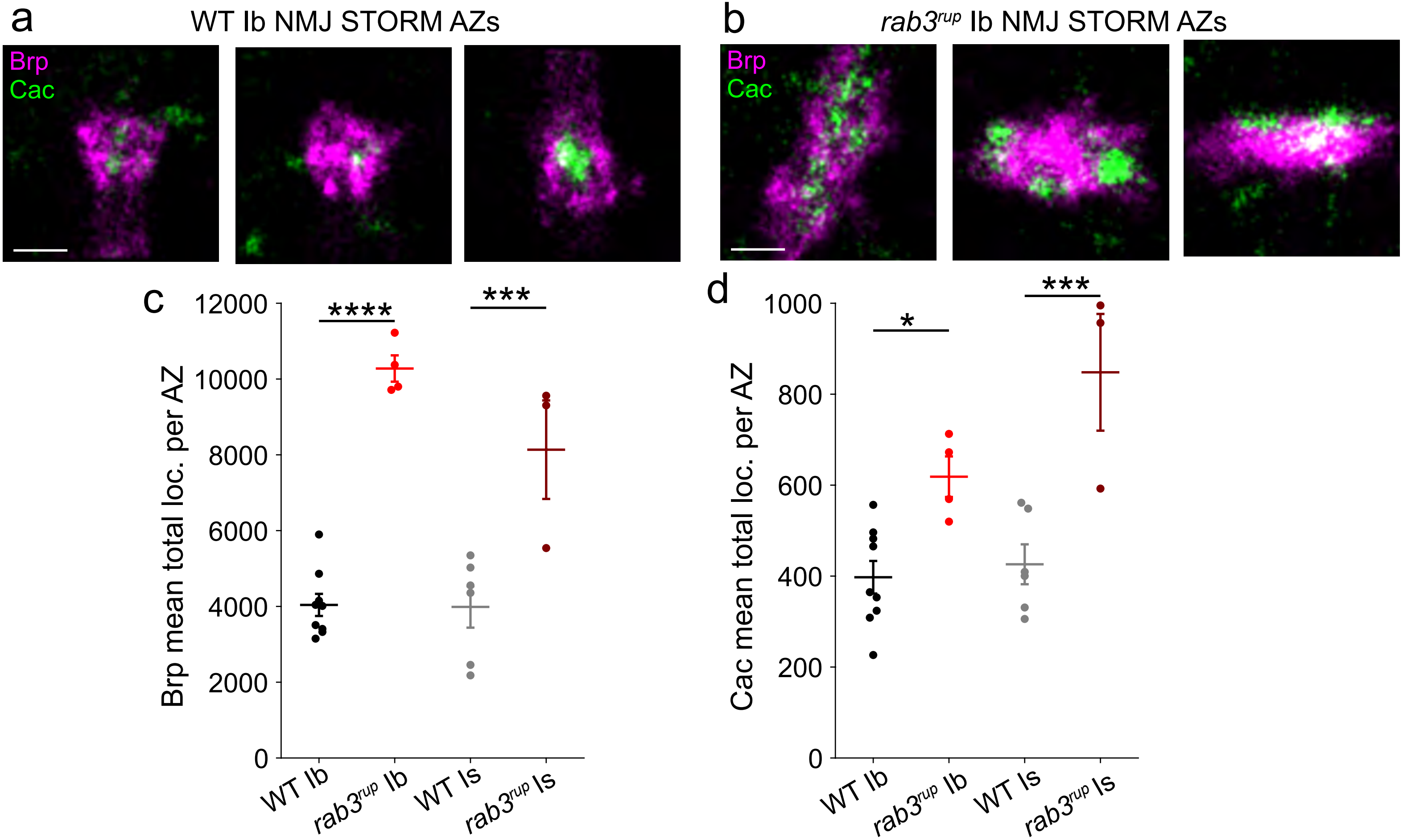

Supplementary Figure 6

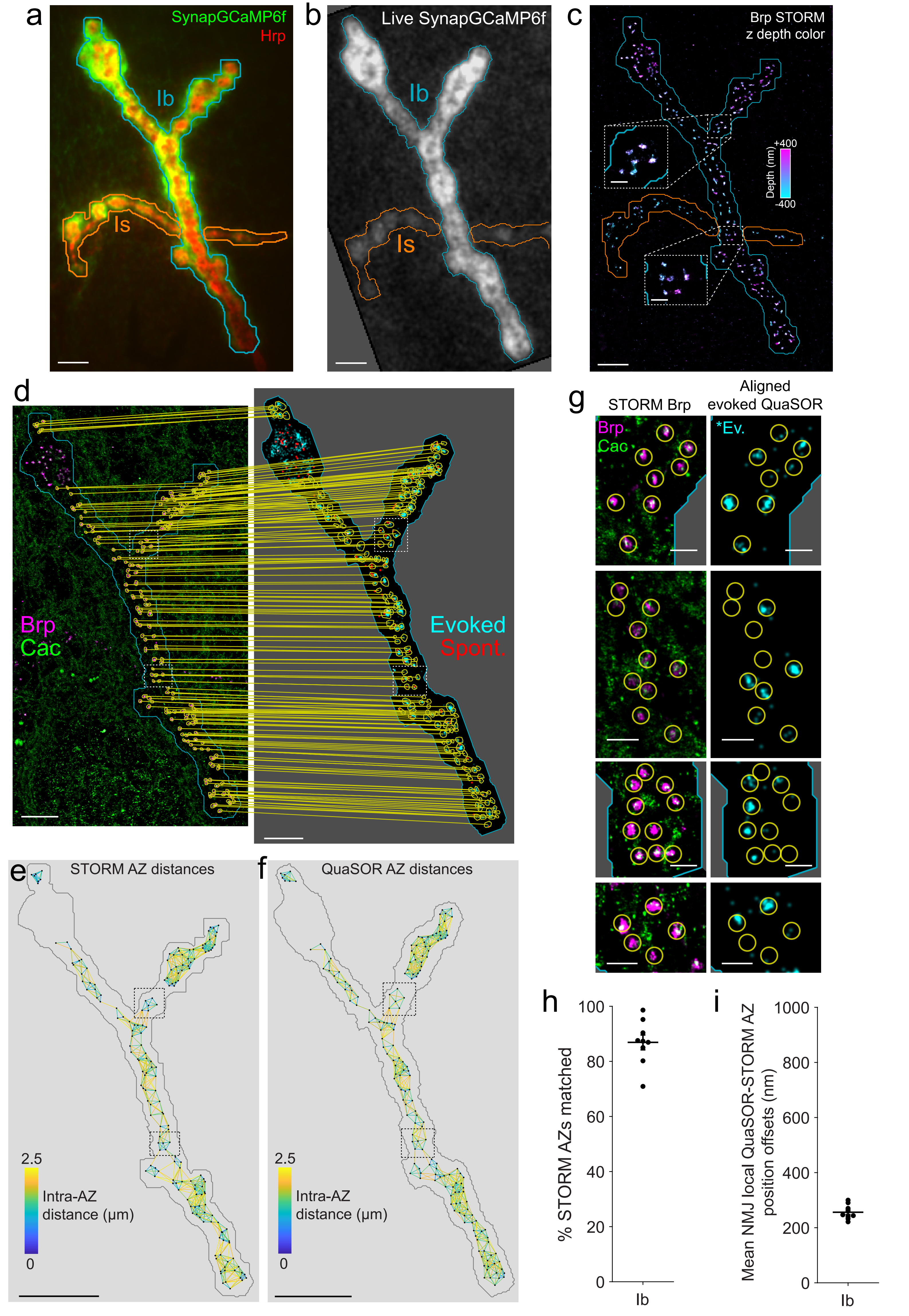

Supplementary Figure 7

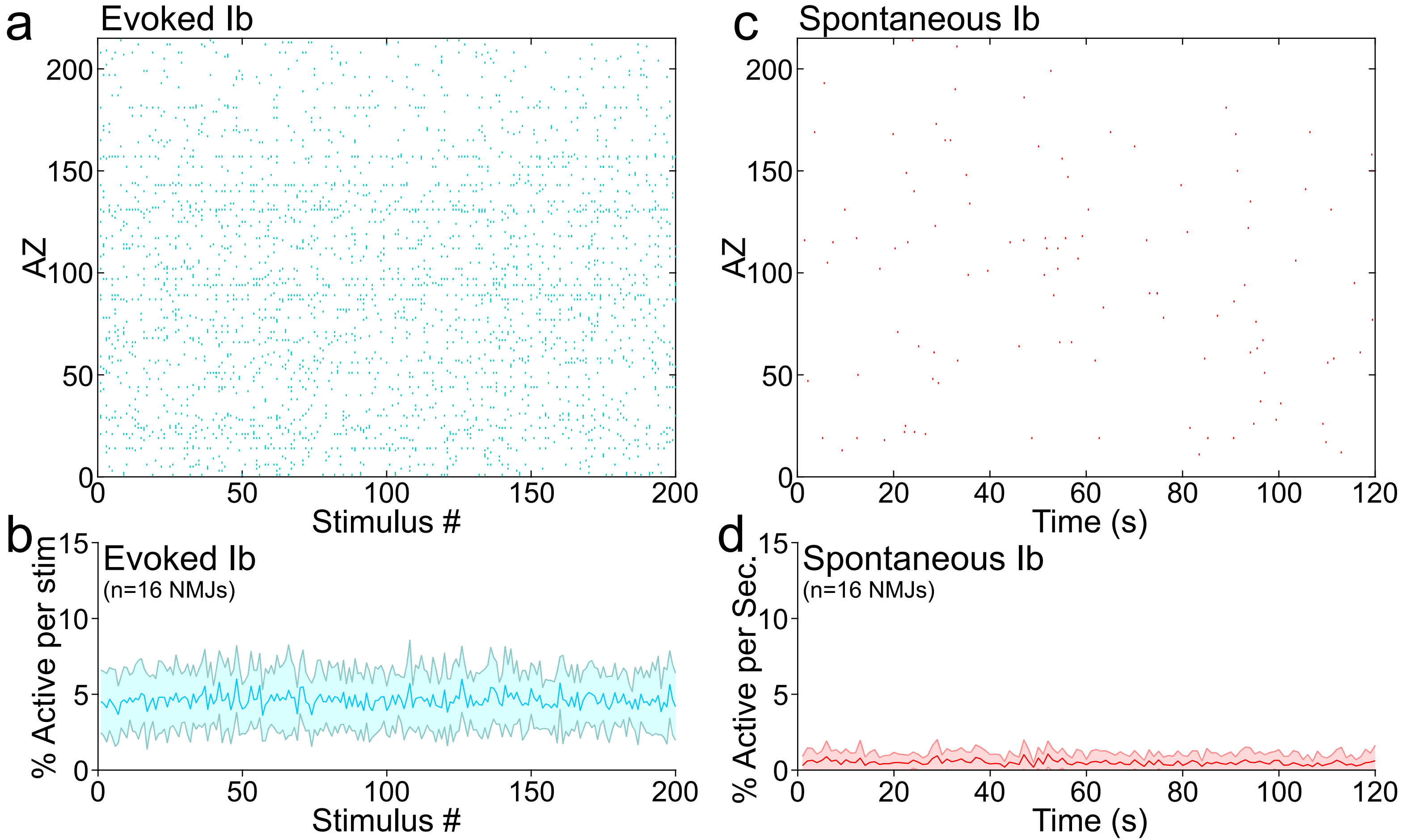

Supplementary Figure 8

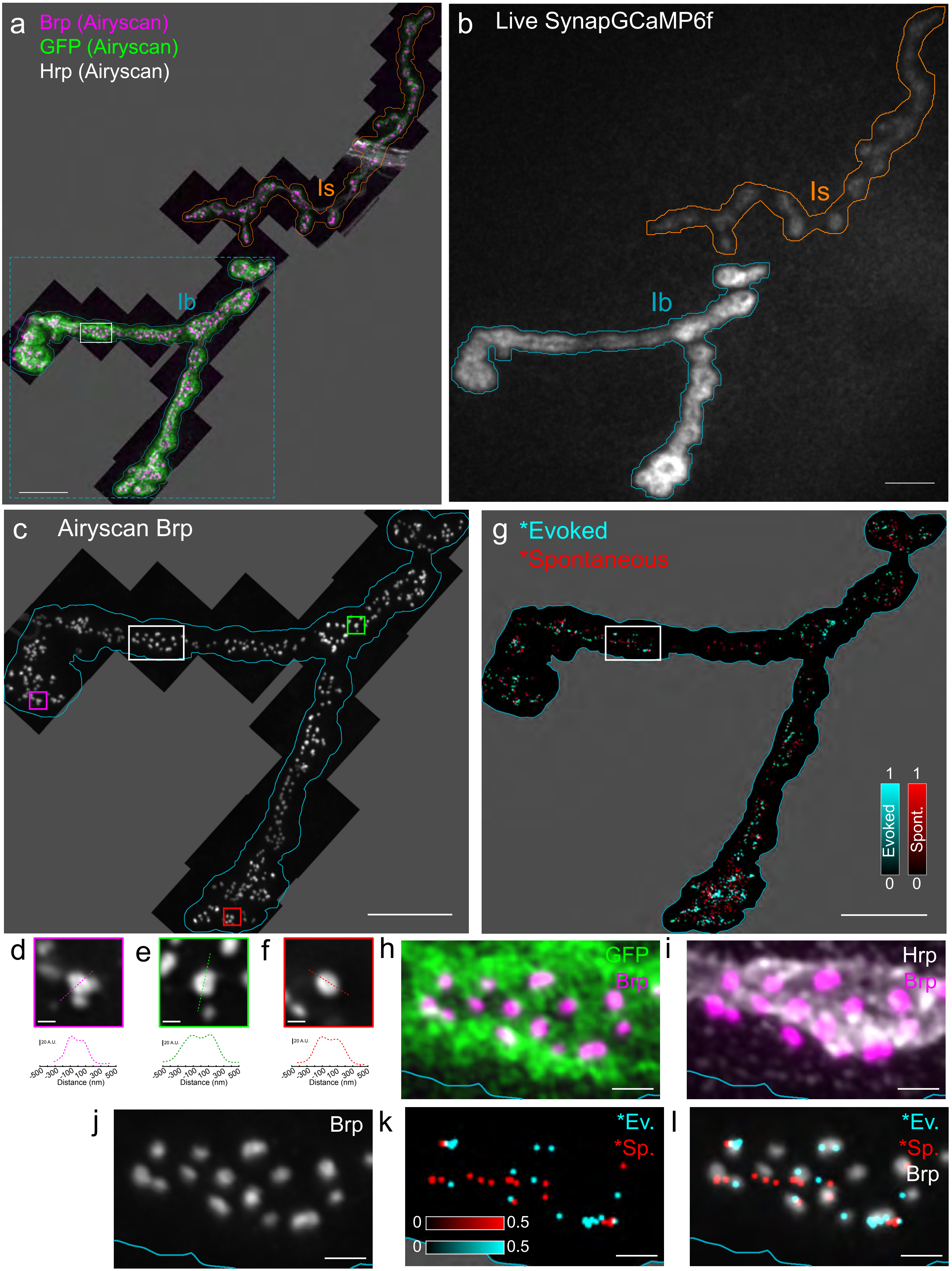

Supplementary Figure 9

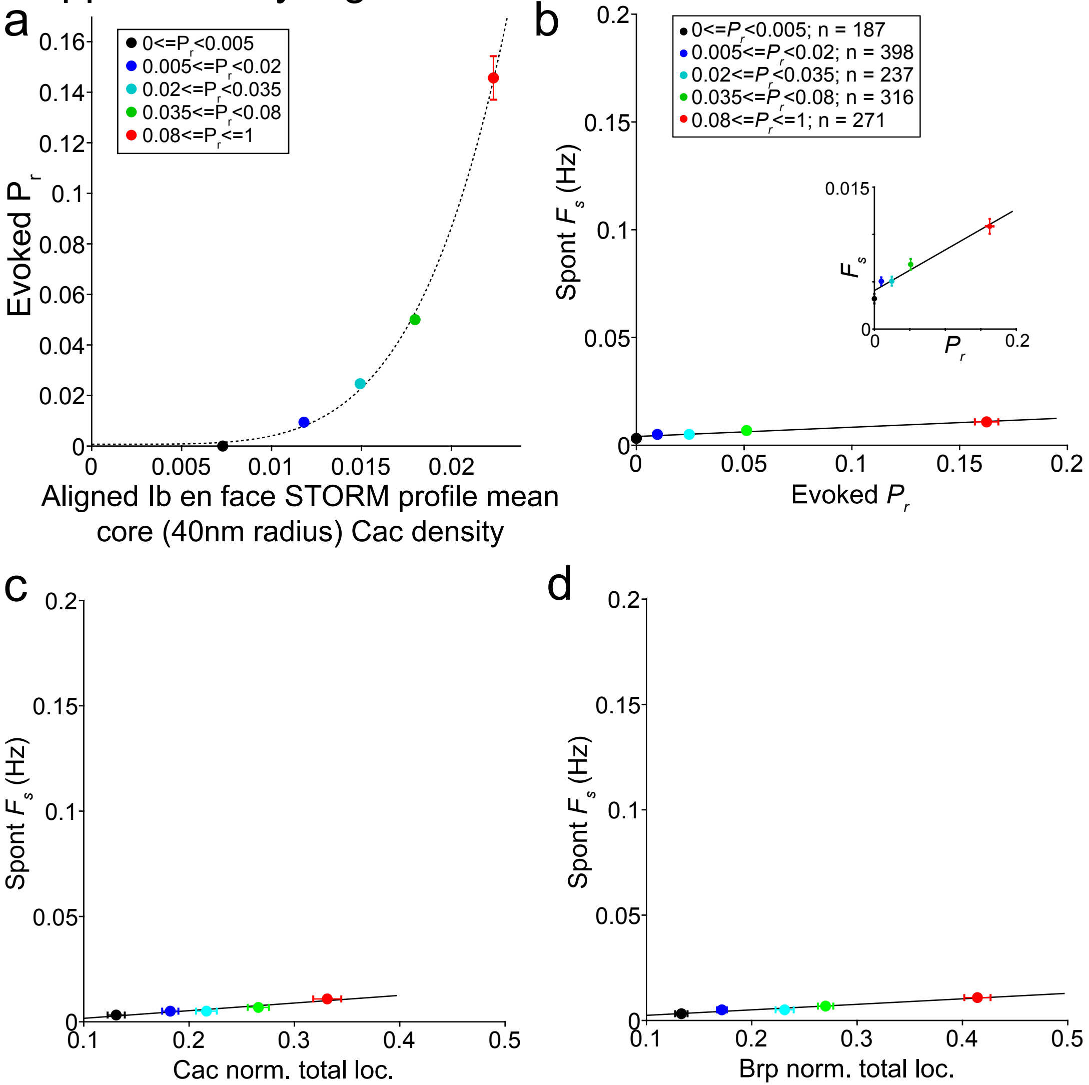

Supplementary Figure 10

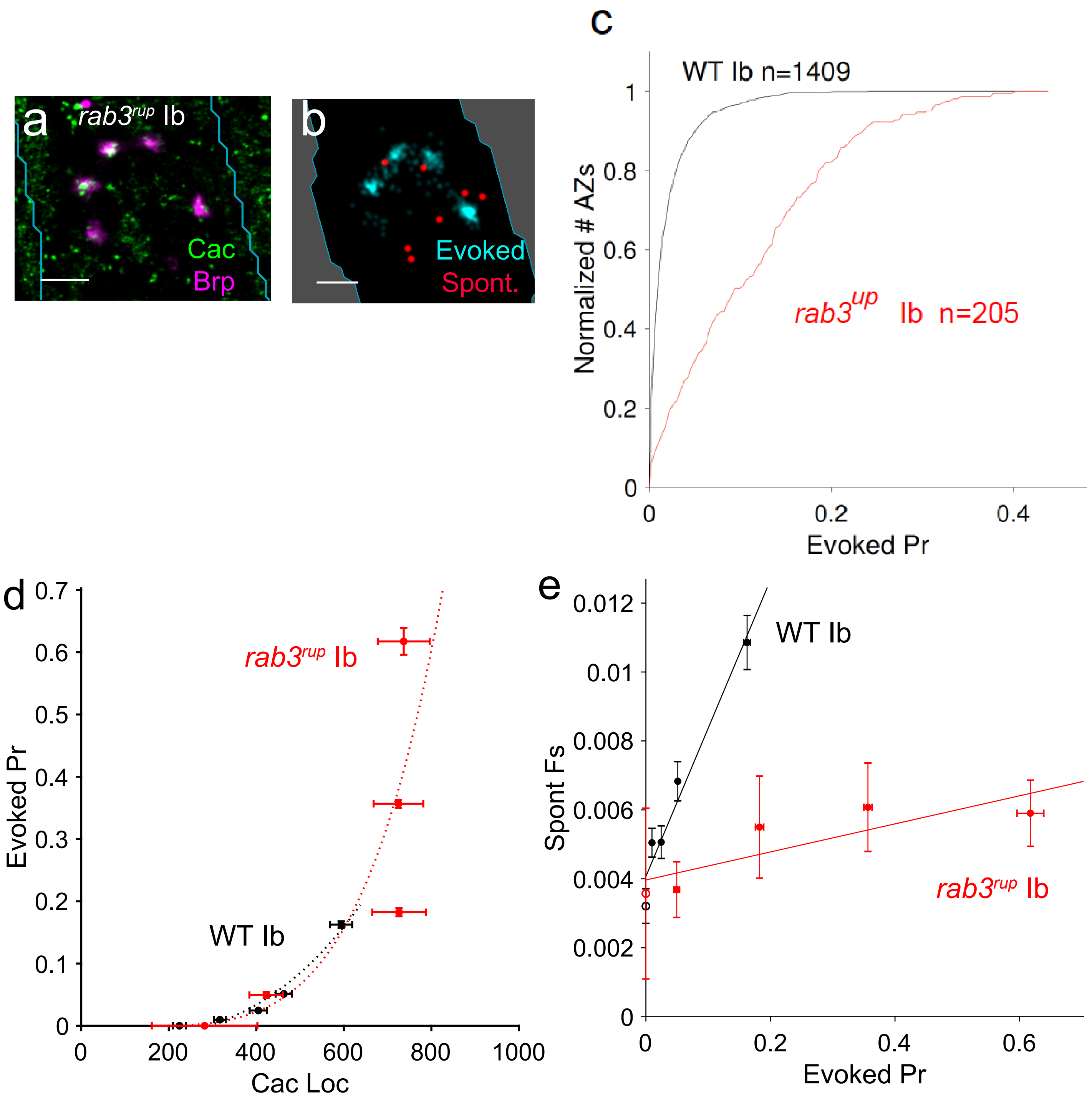

Supplementary Figure 11

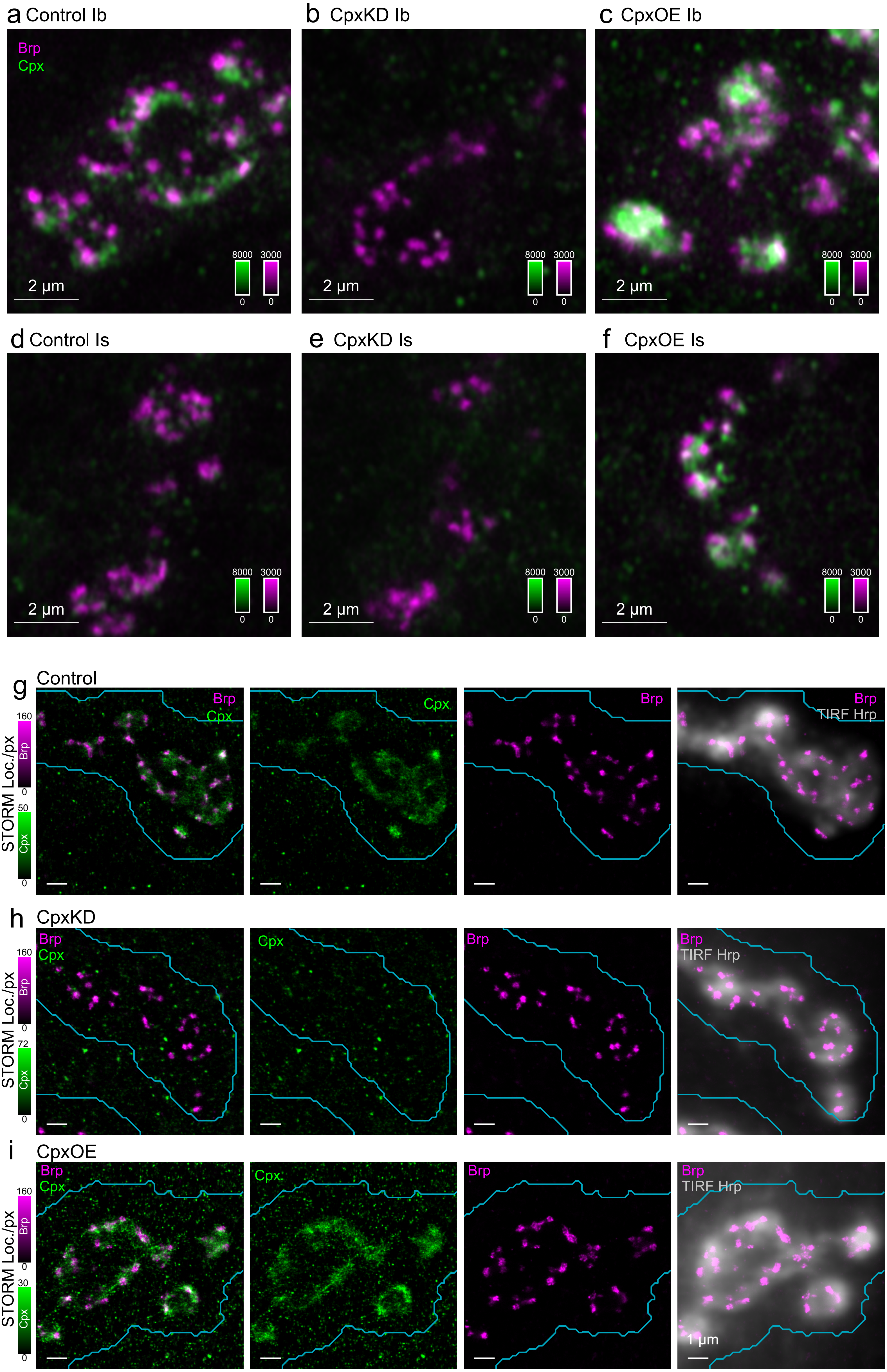

Supplementary Figure 12

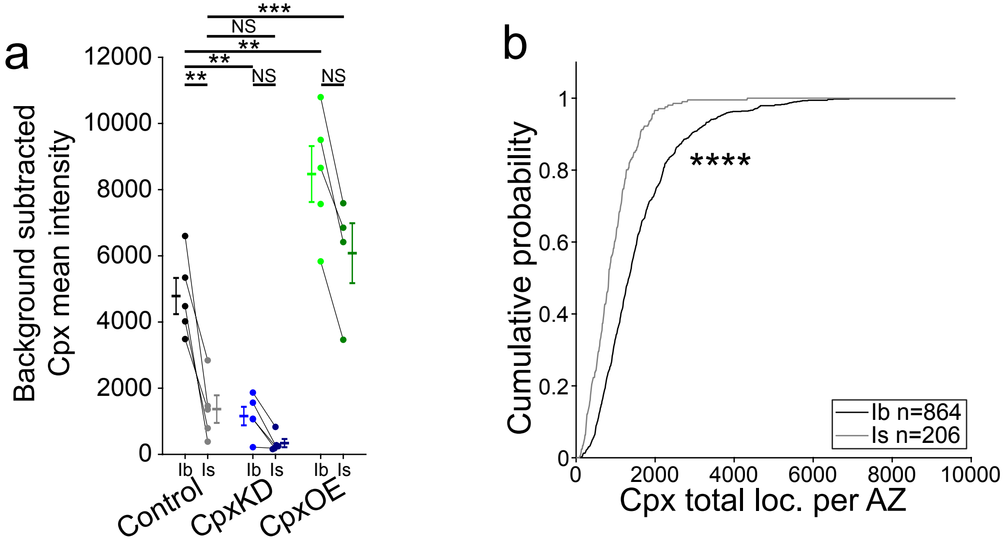

Supplementary Figure 13

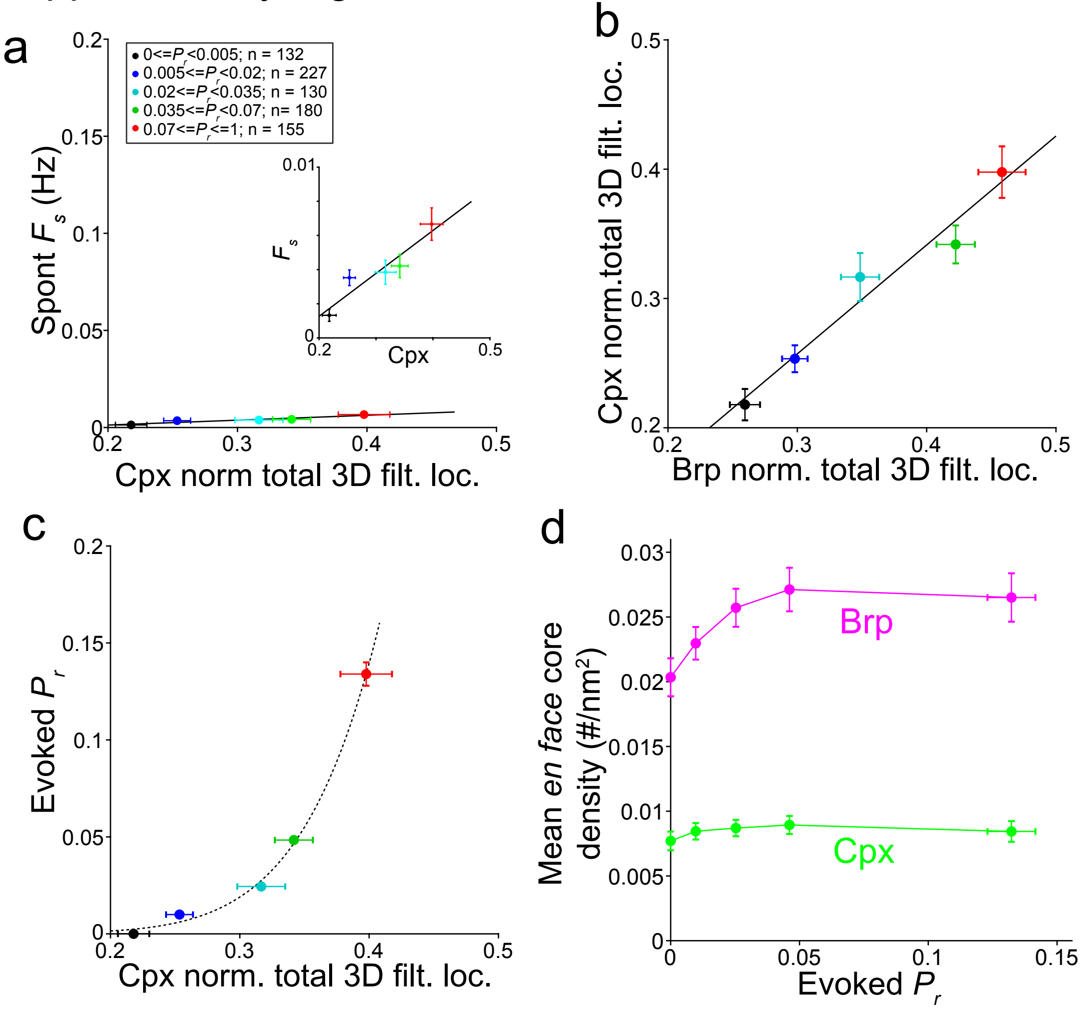

Supplementary Figure 14

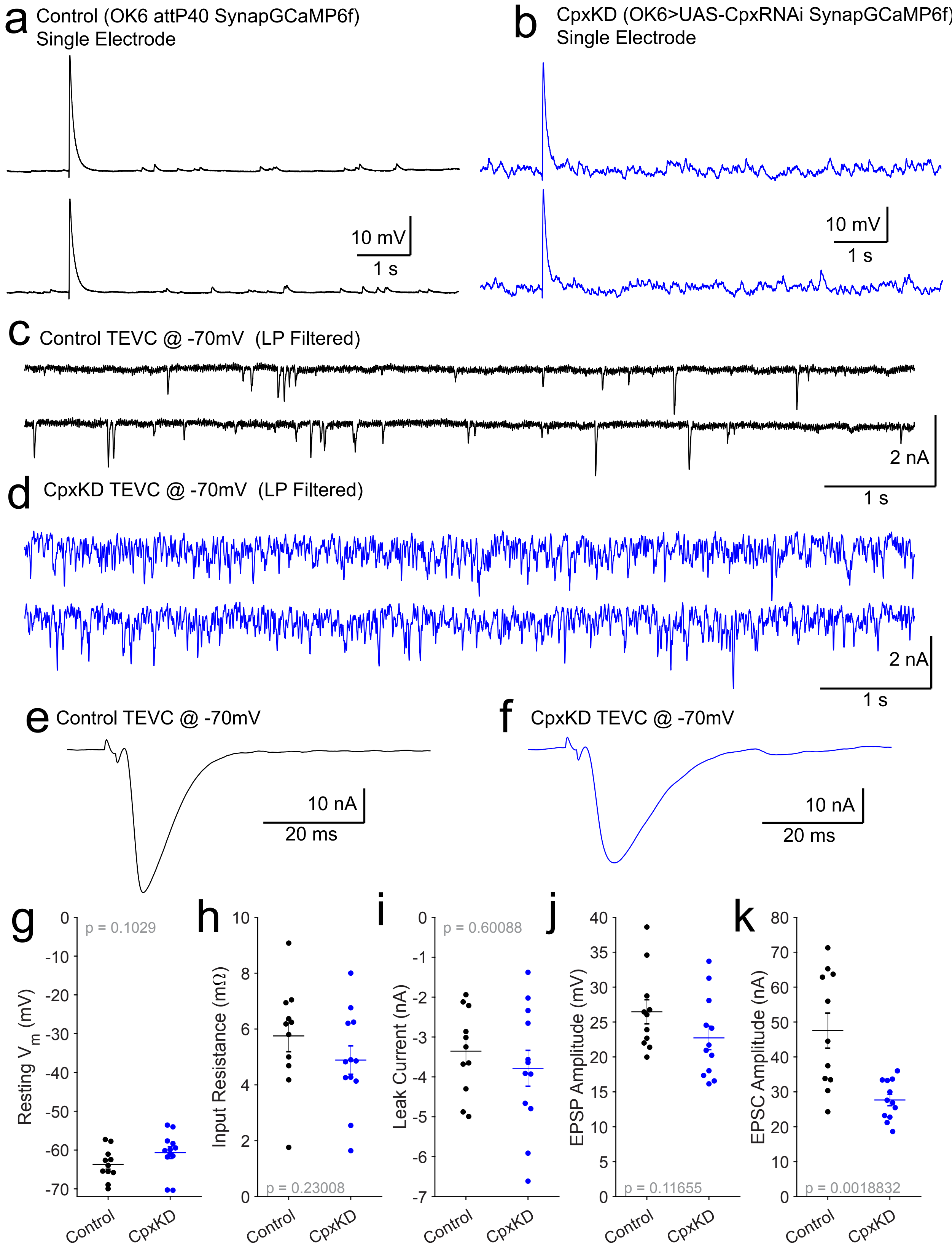

Supplementary Figure 15

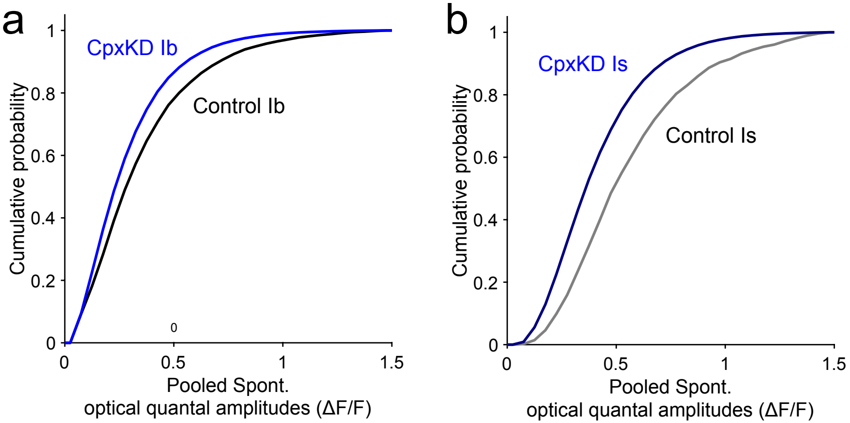

Supplementary Figure 16

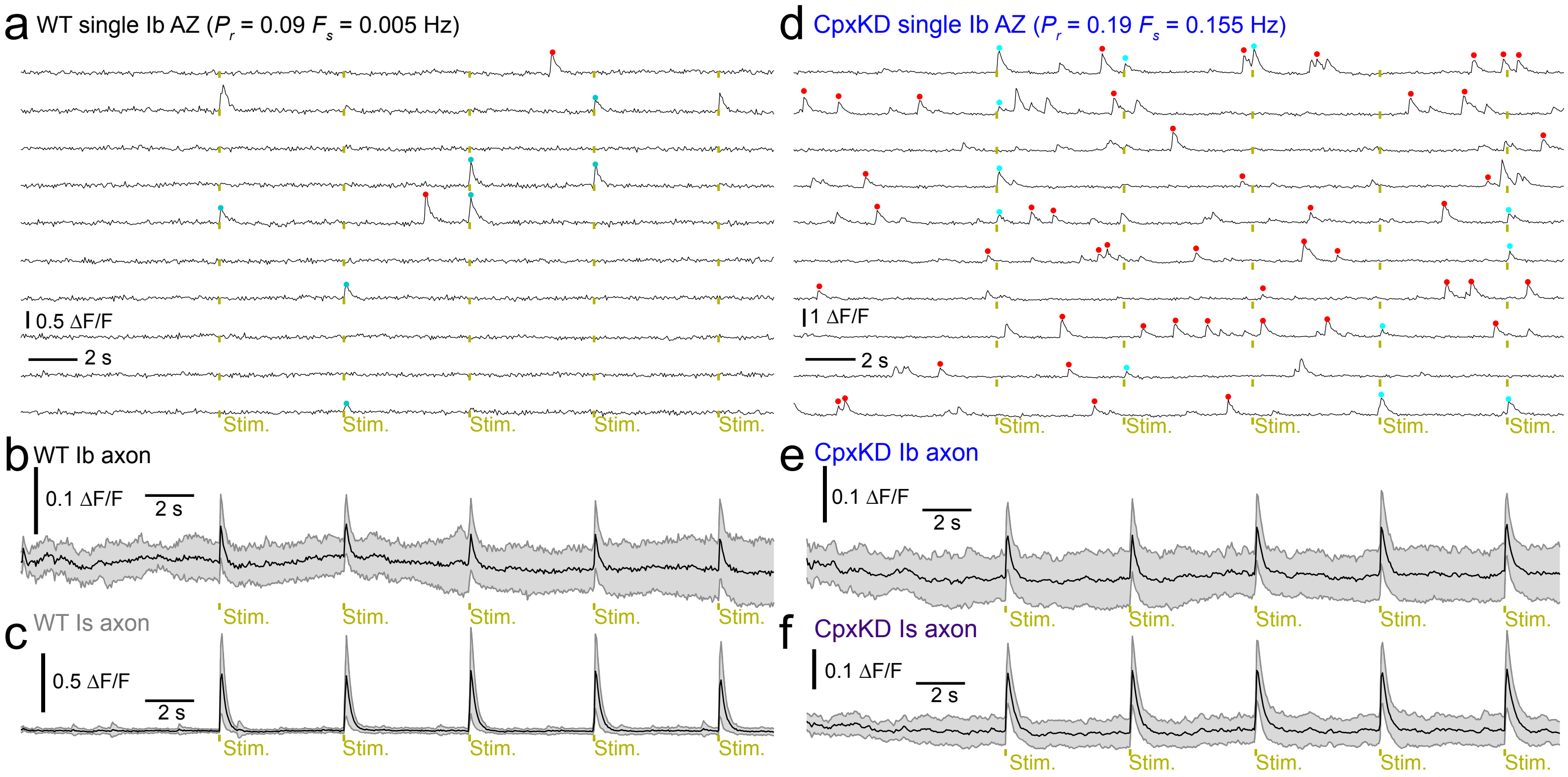

Supplementary Figure 17

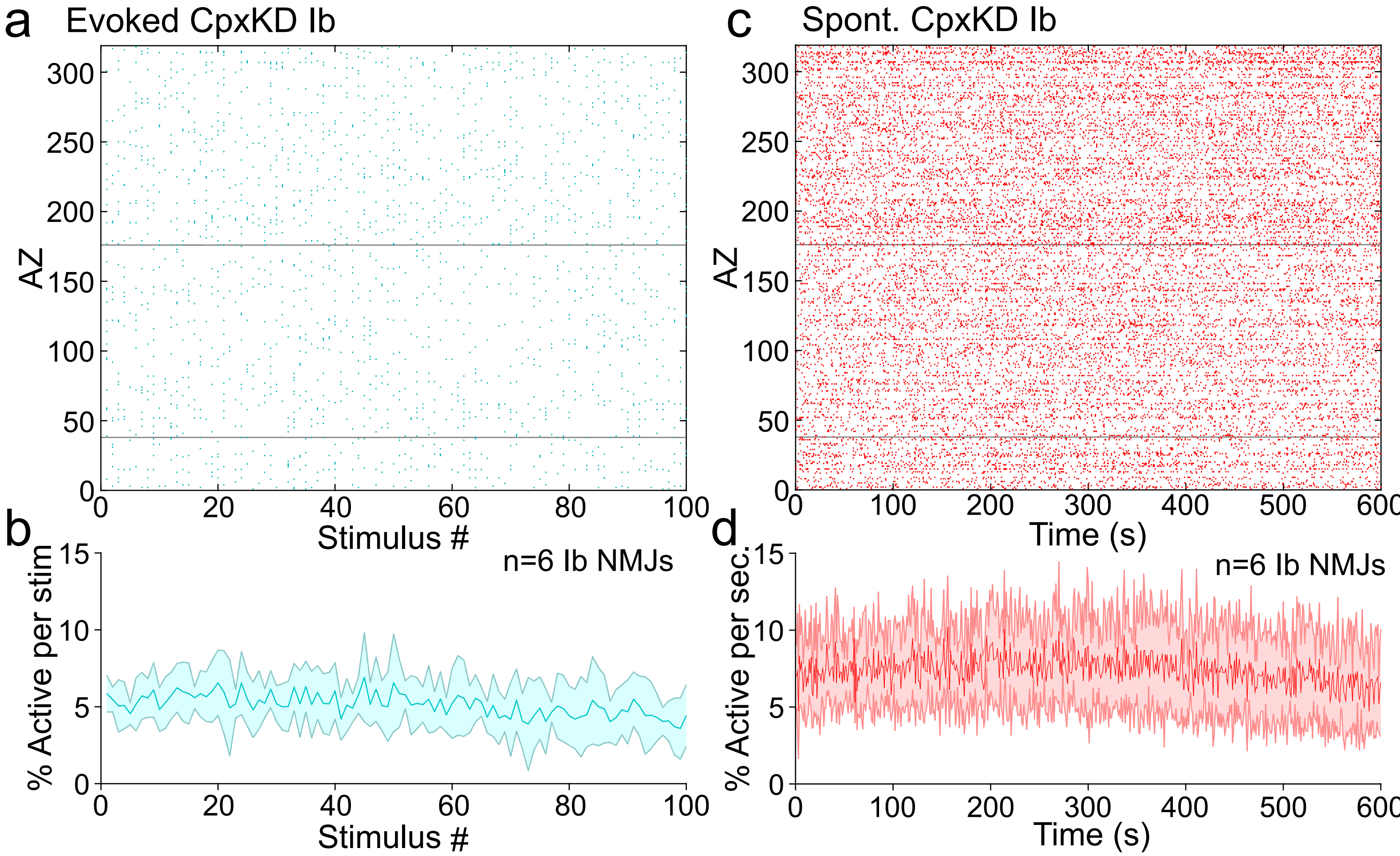

Supplementary Figure 18

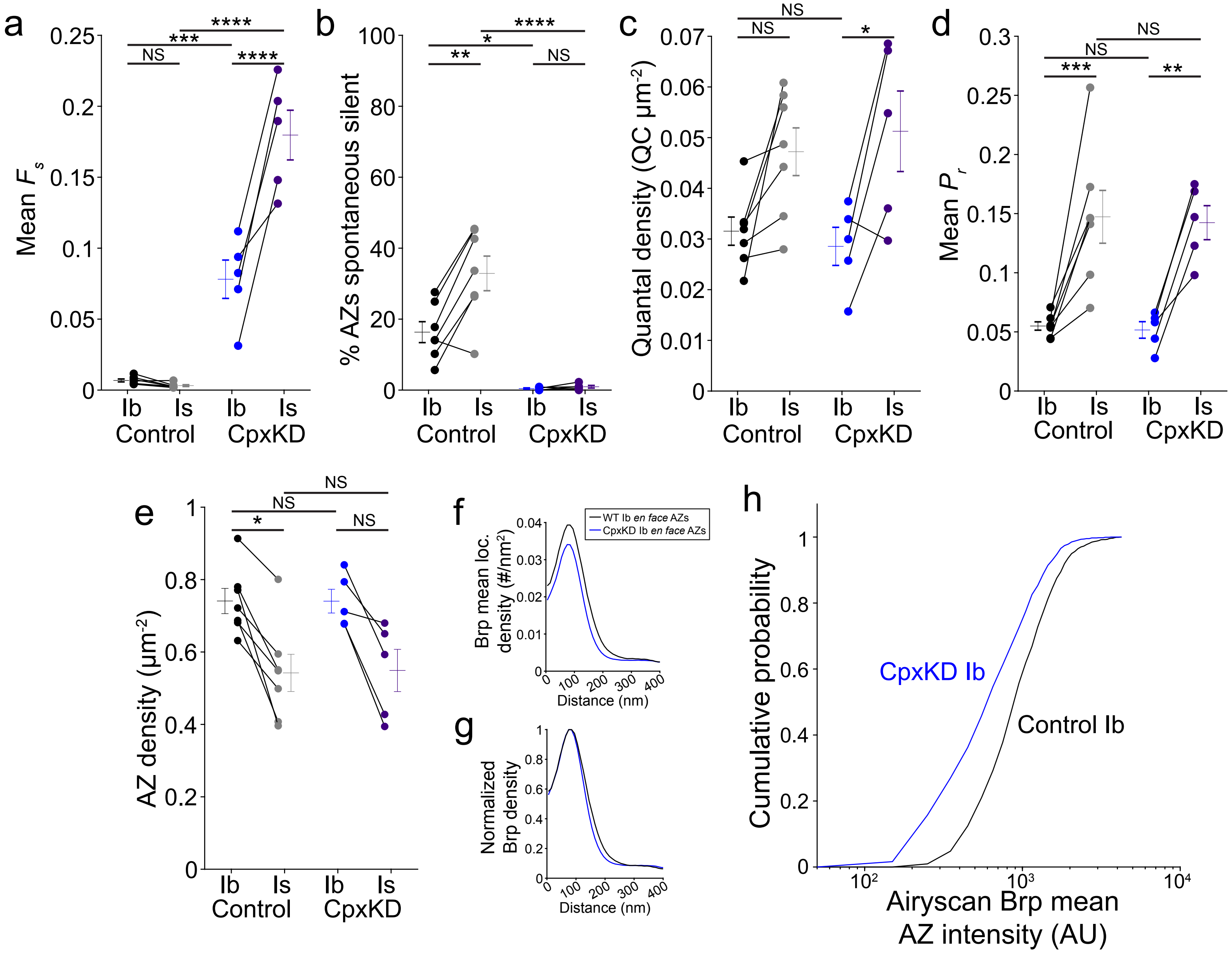

i

	WT Ib NMJs Brp/Alexa647 STORM (n = 407 AZs 2 NMJs)	CpxKD Ib NMJs Brp/Alexa647 STORM (n = 1774 AZs 6 NMJs)
Brp loc. per AZ	2436±62	1904±25****
% Difference		-21.8

	Control Ib NMJs Brp/Alexa405 Airyscan (n = 2333 AZs 7 NMJs)	CpxKD Ib NMJs Brp/Alexa405 Airyscan (n = 1482 AZs 5 NMJs)
Mean Brp intensity[1,2]	1066±12	770±13****
% Difference		-27.7

	Control Ib NMJs Brp/Alexa555 Airyscan (n = 5656 AZs 11 NMJs)	CpxKD Ib NMJs Brp/Alexa555 Airyscan (n = 5010 AZs 10 NMJs)
Mean Brp intensity[3]	3459±18	2660±17****
% Difference		-23.1

**** $p < 0.0001$ Kolmogorov-Smirnov Test

[1] see also Fig. 7O

[2] using automated 3D AZ Mask

[3] using fixed size 3D AZ Mask

Supplementary Figure 19

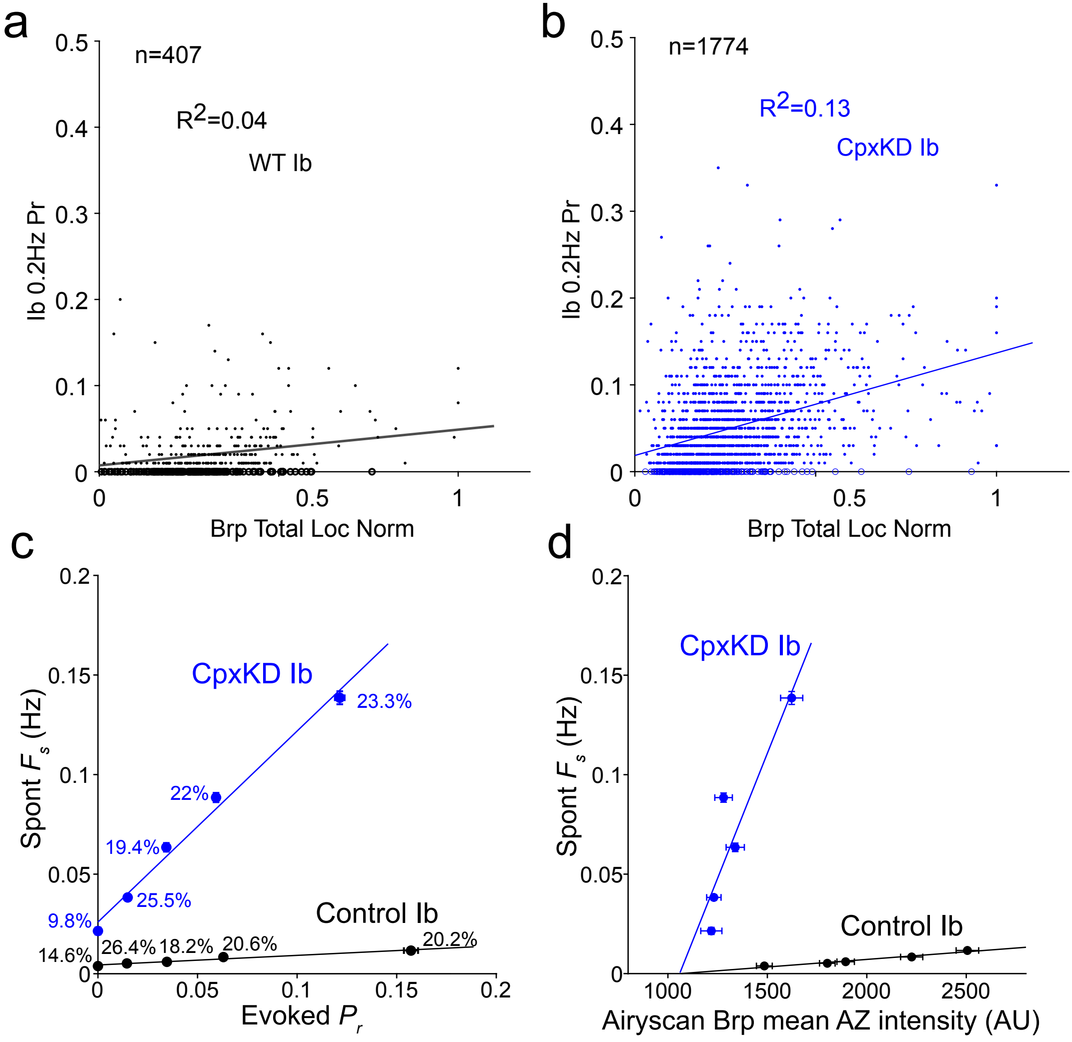

Supplementary Figure 20

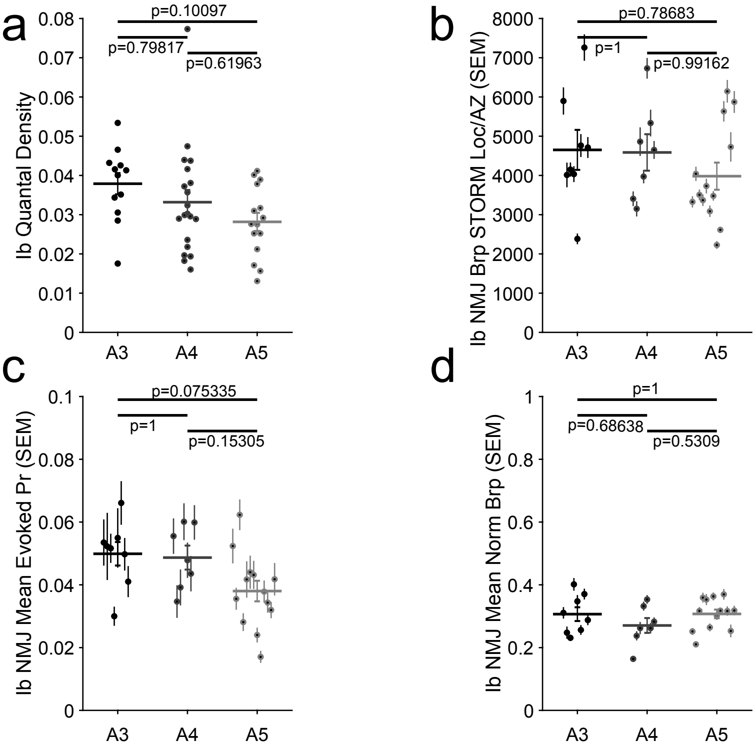

Supplementary Figure 21

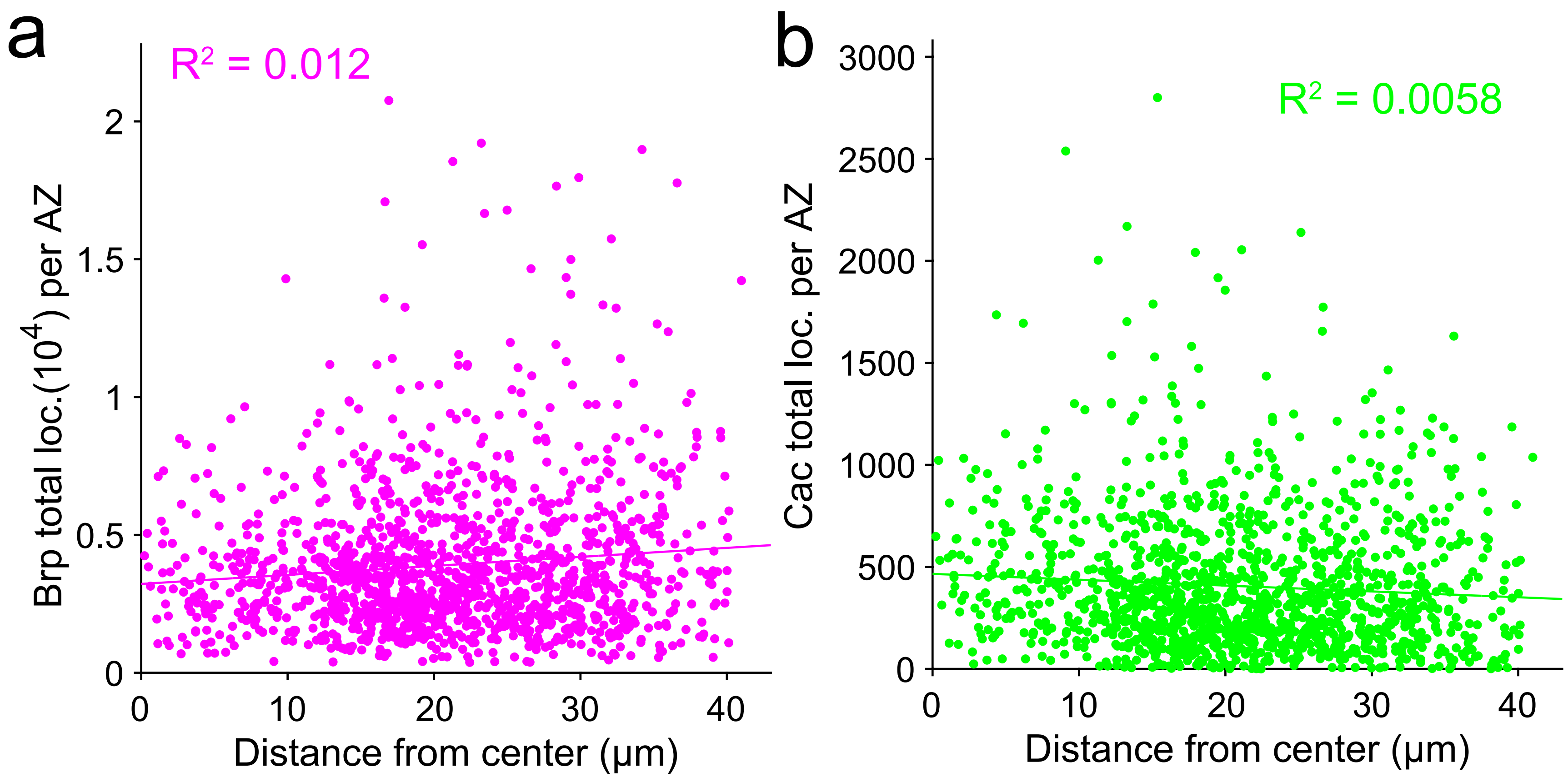

Supplementary Table 1

WT QuaSOR-Structure (combined STORM and Airy) n=33 NMJs
A3: 9
A4: 11
A5: 13

CpxRNAi QuaSOR-Structure (combined STORM and Airy) n=11 NMJs
A3: 5
A4: 3
A5: 3

WT Brp/Cac QuaSOR-STORM n=9 NMJs
A3: 3
A4: 3
A5: 3

WT Brp/Cpx QuaSOR-STORM n=7
A3: 2
A4: 1
A5: 4

WT Brp/Cpx QuaSOR-STORM n=10 NMJs
A3: 3
A4: 3
A5: 4

WT Brp QuaSOR-Airy n=7 NMJs
A3: 1
A4: 4
A5: 2

OK6 attP40 Brp QuaSOR-Airy n=7 NMJs
A3: 6
A4: 1
A5: 0

CpxRNAi Brp QuaSOR-STORM n=6 NMJs
A3: 3
A4: 0
A5: 3

CpxRNAi Brp QuaSOR-Airy n=5 NMJs
A3: 2
A4: 3
A5: 0
