# Supplementary material for: Determinants of synapse diversity revealed by super-resolution quantal transmission and active zone imaging
Source: Nat Commun. 2022 Jan 11;13:229. doi: 10.1038/s41467-021-27815-2 (PMC8752601; doi:10.1038/s41467-021-27815-2)
Supplement: Supplementary file 2 — Description of Additional Supplementary Files [file 41467_2021_27815_MOESM2_ESM.docx]

**Description of Additional Supplementary Files:**

**Supplementary Video 1:** Brp and Cac 3D-STORM provides detailed reconstructions of AZs. An example set of Ib and Is axons imaged with 3D-STORM of Brp and Cac antibodies. Video shows conventional images of SynapGCaMP6f (α-GFP) and the two axons (α-Hrp), followed by 3D-STORM zprojection overlays at increasing resolutions (channels and localization rendering σ values indicated at each resolution). This is followed by identification of all AZs (open white circles), local rendering of each AZ, zooming into two example en face AZs from each axon type and slices through the z depth of each AZ.

**Supplementary Video 2:** STORM spatial averaging provides average en face AZ nanoarchitecture measurements. Video shows the generation of the spatial average images for pooled Brp (magenta) and Cac (green) localizations from STORM-defined en face-oriented WT Ib AZs. Top panel shows the individual AZs aligned to the center region indicated by the circle. Individual AZ localization images were rendered within 800 nm by 800 nm ROIs and then translated to align to the common center (gray borders indicated translated AZs. AZ-specific Pr and Fs values are also indicated along with the NMJ number. Middle panel shows the progression of the average density image. Bottom panel shows the progression of the average radial density profile for the coordinate distributions in the middle panel.

**Supplementary Video 3:** QuaSOR processing workflow for CpxKD provides detailed mapping of spontaneous and evoked transmission events. Video shows the QuaSOR processing pipeline for the CpxKD example in Fig. 6e-j. Data were collected for both Ib and Is axons using the simultaneous spontaneous and evoked imaging protocol where five stimuli were provided for each 30 s movie. In the first half of the video all processing steps are shown with the raw data (top left), the motion and bleach corrected ∆F data (top middle) along with event identifications (magenta circles), the isolated event ∆F/F (top right), the QuaSOR ∆F/F 2D Gaussian models for each event (bottom right), event positions defined by QuaSOR (bottom middle), and the cumulative QuaSOR map for both spontaneous (red) and evoked (cyan) event coordinates. Evoked stimulus timings indicated by *. In the second half of the movie only the cumulative QuaSOR map (left) and the QuaSOR event locations (right) are shown for the remainder of the data collection for this NMJ. 10

**Supplementary Video 4:** QuaSOR-STORM matching and alignment for CpxKD provides robust reconstruction of AZ positions. Video shows the QuaSOR-STORM matching and alignment processing pipeline for the CpxKD example in Fig. 6 and Supplementary Video 3. Video starts by showing the baseline fluorescence image for the live QuaSOR imaging (left) alongside the conventional image of SynapGCaMP6f (α-GFP; green) and the axon membranes (α-Hrp; red) for both Ib and Is axons collected prior to 3D-STORM imaging (right). The QuaSOR region that matches the 3D-STORM data is then indicated and aligned to the 3D-STORM region. The movie then shows a higher QuaSOR map rendering resolution (left), followed by the 3D-STORM zprojection image for Brp and a 3D position colored z-projection image. This is followed by identification of all AZ pairs for QuaSOR ROIs (magenta; left) and STORM ROIs (green; right). These positions are then used to calculate a vector map between all AZ pairs within both axons (left), that is then used to transform the QuaSOR coordinates onto the 3D-STORM pixel space (left) in order to align to the STORM AZ positions. A smaller ROI is then shown for both the aligned QuaSOR (*) map (left) and the Brp 3D-STORM z-projection (right), along with an overlay of the two (right) and then example ∆F/F traces (right) for individual AZs indicated by circles on the QuaSOR map (left). Dots above events indicate that the event originated from that AZ.
