# Supplementary material for: Determinants of synapse diversity revealed by super-resolution quantal transmission and active zone imaging
Source: Nat Commun. 2022 Jan 11;13:229. doi: 10.1038/s41467-021-27815-2 (PMC8752601; doi:10.1038/s41467-021-27815-2)
Supplement: Supplementary file 7 — Reporting Summary [file 41467_2021_27815_MOESM7_ESM.pdf]

nature portfolio

Corresponding author(s): Ehud Isacoff

Last updated by author(s): Jul 21, 2021

Reporting Summary

Nature Portfolio wishes to improve the reproducibility of the work that we publish. This form provides structure for consistency and transparency in reporting. For further information on Nature Portfolio policies, see our Editorial Policies and the Editorial Policy Checklist.

Statistics

For all statistical analyses, confirm that the following items are present in the figure legend, table legend, main text, or Methods section.

n/a	Confirmed	
☐	☒	The exact sample size (*n*) for each experimental group/condition, given as a discrete number and unit of measurement
☐	☒	A statement on whether measurements were taken from distinct samples or whether the same sample was measured repeatedly
☐	☒	The statistical test(s) used AND whether they are one- or two-sided<br>*Only common tests should be described solely by name; describe more complex techniques in the Methods section.*
☐	☒	A description of all covariates tested
☐	☒	A description of any assumptions or corrections, such as tests of normality and adjustment for multiple comparisons
☐	☒	A full description of the statistical parameters including central tendency (e.g. means) or other basic estimates (e.g. regression coefficient) AND variation (e.g. standard deviation) or associated estimates of uncertainty (e.g. confidence intervals)
☐	☒	For null hypothesis testing, the test statistic (e.g. *F*, *t*, *r*) with confidence intervals, effect sizes, degrees of freedom and *P* value noted<br>*Give P values as exact values whenever suitable.*
☒	☐	For Bayesian analysis, information on the choice of priors and Markov chain Monte Carlo settings
☒	☐	For hierarchical and complex designs, identification of the appropriate level for tests and full reporting of outcomes
☐	☒	Estimates of effect sizes (e.g. Cohen's *d*, Pearson's *r*), indicating how they were calculated

Our web collection on statistics for biologists contains articles on many of the points above.

Software and code

Policy information about availability of computer code

Data collection	Data were collected using Clampex 10 (Axon instruments), Slidebook (v6.0.16, 3i), and Zen Black 3.0 (Zeiss).
Data analysis	Data analyzed using custom code in MATLAB R2020a (MathWorks) (GitHub repository: ###_Newman_QuaSOR_2021.m (https://github.com/newmanza/Newman_QuaSOR_2021<br>Airyscan analysis in Fiji (NIH ImageJ Version 2.0.0-rc-43/1.52n)

For manuscripts utilizing custom algorithms or software that are central to the research but not yet described in published literature, software must be made available to editors and reviewers. We strongly encourage code deposition in a community repository (e.g. GitHub). See the Nature Portfolio guidelines for submitting code & software for further information.

Data

Policy information about availability of data

All manuscripts must include a data availability statement. This statement should provide the following information, where applicable:

- Accession codes, unique identifiers, or web links for publicly available datasets
- A description of any restrictions on data availability
- For clinical datasets or third party data, please ensure that the statement adheres to our policy

The data that support the findings of this study are available in the Source data file and in the Figshare repository under the filename Newman QuaSOR 2021 Source Data File.xlsx. “

Field-specific reporting

nature portfolio | reporting summary

Life sciences study design

All studies must disclose on these points even when the disclosure is negative.

Sample size	Sample sizes were determined based on our previous work or similar work in the literature. n = 5 was the minimum replicate number for the entire study. In cases of n=<10, multiple experiments were run (see Supplemental Table 1). These samples sizes are consistent with our previous work (Newman et al., 2017).
Data exclusions	There were no data exclusions for regions where distance between synapses was 350 nm or greater (sufficient to be resolved by QuaSOR) and where global / uniform adjustments brought QuaSOR and STORM into register for assignment.
Replication	In cases of n=<10, multiple experiments were run, as summarized in Suppl. Table 1.
Randomization	test genotypes were always compared to their genetic controls. There was no selection of animals for different treatments, so no randomization of such a process. DBSCAN randomization is no longer relevant because cluster analysis is not included in the final paper.
Blinding	Matches between QuaSOR and STORM were performed by 2-3 investigators who were blind to genotype and to the results by the other investigators. In the case of the STORM matching, Brp STORM-defined AZ areas were matched to clusters of QuaSOR event localizations for 100-200 synapses per NMJ. In the Airyscan-matched data we sorted all QuaSOR event coordinates to their nearest QuaSOR AZ centroid location. The methods produced very similar results. To further ensure unbiased matching, 2 or more people validated the site pairings prior to quantification. All pairs had to minimize the local alignment variance within each bouton as determined by the alignment vector between sites. The quality of the AZ matching was further confirmed by using the paired coordinate positions to generate vectors to transform the QuaSOR coordinates into the matching STORM or Airyscan pixel-space. This was done by first converting the relative QuaSOR coordinates into a matched pixel-space image and then using a 2D, locally weighted, mean transformation method93, with groupings of 8-14 AZs being used as control points in the local weighting. Successful site pairing generated accurate remapping of QuaSOR events onto the appropriate AZ for either structural imaging technique. In all figures these transformed images are denoted by "*Evoked" or "*Spontaneous". However, all quantifications were performed on un-translated data in order to eliminate artifacts of transformations. All other analyses were automated, and utilized identical processing parameters and detection thresholds wherever possible to avoid investigator bias.

Reporting for specific materials, systems and methods

We require information from authors about some types of materials, experimental systems and methods used in many studies. Here, indicate whether each material, system or method listed is relevant to your study. If you are not sure if a list item applies to your research, read the appropriate section before selecting a response.

Materials & experimental systems

n/a	Involved in the study	
☐	☒	Antibodies
☒	☐	Eukaryotic cell lines
☒	☐	Palaeontology and archaeology
☐	☒	Animals and other organisms
☒	☐	Human research participants
☒	☐	Clinical data
☒	☐	Dual use research of concern

Methods

n/a	Involved in the study	
☒	☐	ChIP-seq
☒	☐	Flow cytometry
☒	☐	MRI-based neuroimaging

Antibodies

Antibodies used	The following primary antibodies were used: Mouse anti-Brp (nc82; Developmental Studies Hybridoma Bank, Iowa City, IA); Rabbit anti-Cpx antibody; Chicken anti-GFP (Thermo Fisher A10262; Thermo Fisher Scientific Waltham, MA); Rabbit anti-Cac antibody; goat anti-Hrp (Jackson 123-165-021; Jackson ImmunoResearch Laboratories, West Grove, PA); Alexa Fluor 405 goat anti-mouse (Thermo Fisher A31553), Alexa Fluor 488 goat anti-chicken (Thermo Fisher A11039), Alexa Fluor 488 goat anti-rabbit (Thermo Fisher A11008), Alexa Fluor 555 goat anti-mouse (Thermo Fisher A32727), Alexa Fluor 568 goat anti-rabbit (Thermo Fisher A11036), Alexa Fluor 647 goat anti-rabbit (Thermo Fisher A32733), and Alexa Fluor 647 goat anti-mouse (Thermo Fisher A21235) secondary antibodies; goat, anti-rabbit Biotin F(ab')2 (Jackson 111-066-144).
Validation	For anti-Cac and anti-Cpx antibodies, RNAi KDs showed reduced labeling (Cac: Suppl Fig. 3; Cpx: Suppl. Fig 11). Rabbit anti-Cac (57) (1:1000) was used with goat, anti-rabbit Biotin F(ab')2 (Jackson 111-066-144) at 1:1000, followed by Streptavidin-647 (Thermo Fisher S32357) at 1:500. Rabbit anti-Cpx (47) was used at 1:2000 with Alexa Fluor 488 goat anti-rabbit (Thermo Fisher A11008).

Animals and other organisms

Laboratory animals	Several flies were obtained from the Bloomington Drosophila Stock Center (BDSC) including; attP40Empty (BDSC Line 36304), UAS-CpxRNAi (pVALIUM20 vector; inserted into attP40; BDSC Line 42017), UAS-Cpx (BDSC Line 39743), UAS-CacRNAi (pVALIUM10 vector; inserted into attP2; BDSC Line 27244) and UAS-Dcr2 (BDSC Line 24648). OK6-Gal4 86 and SynapGCaMP6f (3rd chromosome MHC-CD8-GCaMP6f-Sh). The following genotypes were used: WT (w1118; +/+; SynapGCaMP6f/+), Control (w1118; OK6-Gal4/attP40Empty;

SynapGCaMP6f/+), CpxKD (w1118; OK6-Gal4/UAS-CpxRNAi; SynapGCaMP6f/+), CpxOE (w1118; OK6-Gal4/+; UAS-Cpx/ SynapGCaMP6f) and CacKD (UAS-Dcr2/w1118; OK6-Gal4/+; UAS-CacRNAi/SynapGCaMP6f), rab3rup (w1118; rab3rup/DF(2R)ED2076; SynapGCaMP6f/+). Female wandering 3rd instar larvae were used for all experiments.

Wild animals	
Field-collected samples	The study did not include samples collected from the field.
Ethics oversight	All procedures complied with the animal care standards set forth by the National Institutes of Health and were approved by University of California Berkeley's Administrative Panel on Laboratory Animal Care.

Note that full information on the approval of the study protocol must also be provided in the manuscript.
